# Supplementary material for: Three-component contour dynamics model to simulate and analyze amoeboid cell motility in two dimensions
Source: PLoS One. 2024 Jan 26;19(1):e0297511. doi: 10.1371/journal.pone.0297511 (PMC10817190; doi:10.1371/journal.pone.0297511)
Supplement: S1 Text — (PDF) [file pone.0297511.s001.pdf]

Supporting Information S1

Daniel Schindler Ted Moldenhawer Carsten Beta
Wilhelm Huisinga Matthias Holschneider

December 1, 2023

Contents

1 Geometric contour flows **1**

2 Hawkes process: Expected number of offspring **5**

3 Estimation of model weights **6**

4 Numerical implementation of contour dynamics models **7**

5 Model equations with relative weights **9**

6 Ornstein-Uhlenbeck process as protrusion process **11**

1 Geometric contour flows

In this section, we provide additional information on the curve-shortening flow (CSF), the area-preserving curve-shortening flow (APCSF) and the gradient flow of the area functional. For more details about the APCSF, see [1, 2].

Curve-shortening flow. The arc length of a contour Φ is given by the following functional

$$L(\Phi) = \int_0^{2\pi} \left\| \frac{\partial \Phi(\theta)}{\partial \theta} \right\|_2 \, d\theta.$$

In order to derive the corresponding gradient flow, we first deduce the following equality

$$\frac{\partial}{\partial t} L(\Phi) = \int_0^{2\pi} \frac{\frac{\partial \Phi}{\partial \theta}}{\left\| \frac{\partial \Phi}{\partial \theta} \right\|_2} \cdot \frac{\partial^2 \Phi}{\partial \theta \partial t} \, d\theta$$

$$
\begin{aligned}
&= \left[\frac{\frac{\partial \Phi}{\partial \theta}}{\left\| \frac{\partial \Phi}{\partial \theta} \right\|_2} \cdot \frac{\partial \Phi}{\partial t} \right]_0^{2\pi} - \int_0^{2\pi} \frac{\partial \Phi}{\partial t} \cdot \frac{\partial}{\partial \theta} \left(\frac{\frac{\partial \Phi}{\partial \theta}}{\left\| \frac{\partial \Phi}{\partial \theta} \right\|_2} \right) d\theta \\
&= 0 - \int_0^{2\pi} \frac{\partial \Phi}{\partial t} \cdot \vec{t}' \, d\theta \\
&= - \int_0^{L(\Phi)} \frac{\partial \Phi}{\partial t} \cdot \frac{\vec{t}'}{\left\| \frac{\partial \Phi}{\partial \theta} \right\|_2} \, ds \\
&= - \int_0^{L(\Phi)} \frac{\partial \Phi}{\partial t} \cdot \kappa \, \vec{n}_{\text{inw}} \, ds
\end{aligned}
$$

Hence, the most rapid decreasing of $L(\Phi)$ is given by

$$
\frac{\partial \Phi}{\partial t} = \kappa \, \vec{n}_{\text{inw}}
$$

which is known as the curve-shortening flow. The rate of the CSF is then given by

$$
\begin{aligned}
\frac{\partial}{\partial t} L(\Phi) &= - \int_0^{L(\Phi)} \kappa^2 \, \|\vec{n}_{\text{inw}}\|_2^2 \, ds \\
&= - \int_0^{L(\Phi)} \kappa^2 \, ds,
\end{aligned}
$$

where $\int_0^{L(t)} \kappa^2 \, ds$ is called the total squared curvature.

Gradient flow of the area functional. As an alternative to the area-adjustment flow (AAF) from the main article, we propose the gradient flow of the area functional. First, we remind that the area of a contour Φ is given by the following functional

$$
A(\Phi) = \int_0^{2\pi} \Phi^{(x)} \, d\Phi^{(y)} = \int_0^{2\pi} \Phi^{(x)} \frac{\partial \Phi^{(y)}}{\partial \theta} \, d\theta.
$$

Then, the first variation of $A(\Phi)$ is given by

$$
\begin{aligned}
\delta A(\Phi, h) &= \frac{d}{d\varepsilon} A(\Phi + \varepsilon h) \Big|_{\varepsilon = 0} \\
&= \frac{d}{d\varepsilon} \int_0^{2\pi} (\Phi_x + \varepsilon h_x) \frac{\partial \left(\Phi_y + \varepsilon h_y \right)}{\partial \theta} \, d\theta \Big|_{\varepsilon = 0} \\
&= \frac{d}{d\varepsilon} \int_0^{2\pi} (\Phi_x + \varepsilon h_x) \left(\frac{\partial \Phi_y}{\partial \theta} + \varepsilon \frac{\partial h_y}{\partial \theta} \right) d\theta \Big|_{\varepsilon = 0} \\
&= \frac{d}{d\varepsilon} \int_0^{2\pi} \Phi_x \frac{\partial \Phi_y}{\partial \theta} + \varepsilon \Phi_x \frac{\partial h_y}{\partial \theta} + \varepsilon h_x \frac{\partial \Phi_y}{\partial \theta} + \varepsilon^2 h_x \frac{\partial h_y}{\partial \theta} \, d\theta \Big|_{\varepsilon = 0} \\
&= \int_0^{2\pi} \Phi_x \frac{\partial h_y}{\partial \theta} + h_x \frac{\partial \Phi_y}{\partial \theta} \, d\theta
\end{aligned}
$$

$$
\begin{aligned}
&= [\Phi_x h_y]_0^{2\pi} - \int_0^{2\pi} h_y \frac{\partial \Phi_x}{\partial \theta}\, d\theta + \int_0^{2\pi} h_x \frac{\partial \Phi_y}{\partial \theta}\, d\theta \\
&= \int_0^{2\pi} h_x \frac{\partial \Phi_y}{\partial \theta} - h_y \frac{\partial \Phi_x}{\partial \theta}\, d\theta \\
&= \int_0^{2\pi} \langle h, \begin{pmatrix} \frac{\partial \Phi_y}{\partial \theta} \\ -\frac{\partial \Phi_x}{\partial \theta} \end{pmatrix} \rangle\, d\theta = \int_0^{2\pi} \langle h, \begin{pmatrix} \frac{\partial \Phi_x}{\partial \theta} \\ \frac{\partial \Phi_y}{\partial \theta} \end{pmatrix}^{\perp} \rangle\, d\theta \\
&= \int_0^{L(\Phi)} \langle h, \frac{\left(\frac{\partial \Phi}{\partial \theta}\right)^{\perp}}{\|\frac{\partial \Phi}{\partial \theta}\|} \rangle\, ds = \int_0^{L(\Phi)} \langle h, \vec{t}^{\perp} \rangle\, ds \\
&= \int_0^{L(\Phi)} \langle h, \vec{n} \rangle\, ds
\end{aligned}
$$

and therefore

$$
\delta A(\Phi, h) = -\int_0^{L(\Phi)} \langle h, \vec{n}_{\text{inw}} \rangle\, ds \tag{1.1}
$$

Now, for the direction $h = \frac{\partial \Phi}{\partial t} = \vec{n}_{\text{inw}}$, the area functional $A(\Phi)$ decreases most rapidly with the following rate:

$$
\frac{\partial A(\Phi)}{\partial t} = -\int_0^{L(\Phi)} \|\vec{n}_{\text{inw}}\|_2^2\, ds = -L(\Phi)
$$

Similarly, we can compute the gradient flow of the following "area adjustment functional":

$$
F(\Phi) \coloneqq (A(\Phi) - A_{\text{ref}})^2 \to \min,
$$

where $A_{\text{ref}} \in \mathbb{R}^+$ denotes the desired mean area. By using Eq (1.1), we compute the first variation

$$
\delta F(\Phi, h) = -2\,(A(\Phi) - A_{\text{ref}}) \int_0^{L(\Phi)} \langle h, \vec{n}_{\text{inw}} \rangle\, ds,
$$

which decreases most rapidly for $h = \frac{\partial \Phi}{\partial t} = -2\,(A(\Phi) - A_{\text{ref}})\,\vec{n}_{\text{inw}}$ with rate

$$
\frac{\partial F}{\partial t} = -2(A(\Phi) - A_{\text{ref}})\; L(\Phi).
$$

Noteworthy, this gradient flow affects the contour in normal direction only. However, we recommend the use of the AAF from the main article because of its shape-preserving property.

Area-preserving curve-shortening flow. We remind that the area-preserving curve-shortening flow (APCSF) of a contour Φ is given by

$$
\frac{\partial \Phi}{\partial t} = \left(\kappa - \frac{2\pi}{L(\Phi)}\right) \vec{n}_{\text{inw}}.
$$

By inserting $h = \frac{\partial \Phi}{\partial h}$ into Eq (1.1), we can show that the APCSF is indeed area-preserving:

$$\begin{aligned}
\frac{\partial A(\Phi)}{\partial t} &= -\int_0^{L(\Phi)} \langle h, \vec{n}_{\text{inw}} \rangle \, ds \\
&= -\int_0^{L(\Phi)} \left(\kappa - \frac{2\pi}{L(\Phi)} \right) \|\vec{n}_{\text{inw}}\|_2^2 \, ds \\
&= -\int_0^{L(\Phi)} \left(\kappa - \frac{2\pi}{L(\Phi)} \right) \, ds \\
&= -\int_0^{L(\Phi)} \kappa \, ds + \int_0^{L(\Phi)} \frac{2\pi}{L(\Phi)} \, ds \\
&= -2\pi + L(\Phi) \frac{2\pi}{L(\Phi)} = 0.
\end{aligned}$$

Furthermore, we can compute the decay of $L(\Phi)$ under the APCSF via:

$$\begin{aligned}
\frac{\partial L(\Phi)}{\partial t} &= -\int_0^{L(\Phi)} \frac{\partial \Phi}{\partial t} \cdot \kappa \, \vec{n}_{\text{inw}} \, ds \\
&= -\int_0^{L(\Phi)} \left(\kappa - \frac{2\pi}{L(\Phi)} \right) \vec{n}_{\text{inw}} \cdot \kappa \, \vec{n}_{\text{inw}} \, ds \\
&= -\int_0^{L(\Phi)} \left(\kappa^2 - \kappa \frac{2\pi}{L(\Phi)} \right) \|\vec{n}_{\text{inw}}\|_2^2 \, ds \\
&= -\int_0^{L(\Phi)} \kappa^2 \, ds + \frac{2\pi}{L(\Phi)} \int_0^{L(\Phi)} \kappa \, ds \\
&= -\int_0^{L(\Phi)} \kappa^2 \, ds + \frac{4\pi^2}{L(\Phi)}
\end{aligned}$$

Furthermore, the following equality holds:

$$\begin{aligned}
-\int_0^{L(\Phi)} \left(\kappa - \frac{2\pi}{L(\Phi)} \right)^2 \, ds &= -\int_0^{L(\Phi)} \kappa^2 - \frac{4\pi}{L(\Phi)} \kappa + \frac{4\pi^2}{L(\Phi)^2} \, ds \\
&= -\int_0^{L(\Phi)} \kappa^2 \, ds + \frac{8\pi^2}{L(\Phi)} - \frac{4\pi^2}{L(\Phi)} \\
&= -\int_0^{L(\Phi)} \kappa^2 \, ds + \frac{4\pi^2}{L(\Phi)}
\end{aligned}$$

Then, we can show that the APCSF is indeed an arc length shortening flow:

$$\frac{\partial L(\Phi)}{\partial t} = -\int_0^{L(\Phi)} \left(\kappa - \frac{2\pi}{L(\Phi)} \right)^2 \, ds \leq 0,$$

with $\frac{\partial L(\Phi)}{\partial t} = 0$ if and only if Φ is a circle with radius $r = \frac{1}{\kappa} = \frac{L}{2\pi}$.

2 Hawkes process: Expected number of offspring

As described in the main paper, the underlying kernel of the Hawkes process is given by $g(t, \theta) = g_1(t) \cdot g_2(\theta)$, separated into a temporal component $g_1(t)$ and a spatial component $g_2(\theta)$. In this context, the temporal kernel is given by

$$g_1(t) = \alpha\beta\, t\, e^{-\beta t},$$

with arrival intensity $\alpha > 0$ and exponential decay rate $\beta > 0$. Furthermore, the spatial kernel is defined as the von Mises distribution

$$g_2(\theta) = \frac{e^{\kappa_M \cos(\theta)}}{2\pi I_0(\kappa_M)},$$

with $\kappa_M > 0$ as concentration parameter and $I_0(\kappa_M)$ denoting the modified Bessel function of order 0.

The expected number of offspring under this Hawkes process is then given by the following integral

$$m = \int_0^{2\pi} \int_0^{\infty} g(t, \theta)\; dt\; d\theta.$$

Since g_1 and g_2 are continuous and, therefore, g as well, we can apply Fubini's theorem. Furthermore, due to g_2 being a probability density function, we obtain

$$\begin{aligned} m &= \int_0^{2\pi} g_2(\theta)\; d\theta \cdot \int_0^{\infty} g_1(t)\; dt \\ &= 1 \cdot \int_0^{\infty} \alpha\beta\, t\, e^{-\beta t}\; dt \\ &= \left[-\frac{\alpha e^{-\beta t}\, (\beta t + 1))}{\beta} \right]_0^{\infty} \\ &= \frac{\alpha}{\beta} \end{aligned}$$

For the parameters $\alpha = 0.5s^{-1}$ and $\beta = 0.4s^{-1}$ used in our paper, we obtain the expected number of offspring $m = 0.8$. Given a background intensity $\lambda_0 = 1s^{-1}$ and a time span $T = 500s$, we can compute the expected number of events

$$\lambda_0 \cdot T \cdot \sum_{k=0}^{\infty} m^k = 2500.$$

Therefore, to obtain the same amount of events with a Poisson process, i.e., with one generation of events only, the background intensity must be set to $\lambda_0 = 5s^{-1}$.

3 Estimation of model weights

In this section, we describe our approach how to estimate the model weights w_{prot}, w_{APCSF}, and w_{AAF} when replicating experimental cell tracks with our model.

In this context, we will make use of the local motion kymograph derived from the experimental contour dynamics; see [3] for more details. More precisely, we tune f_{APCSF} and f_{AAF} such that negative regions (i.e. retractions) of the local motion are mainly captured by the above components. For this reason, we introduce the residuals $r_{k,i}$ defined by

$$\begin{aligned} r_{k,i} &= \text{LM}_{k,i} - f_{\text{APCSF}}\left(t_k, \theta_i\right) - f_{\text{AAF}}\left(t_k, \theta_i\right) \\ &\approx f_{\text{prot}}(t_k, \theta_i) \end{aligned}$$

with local motion $\text{LM}_{k,i}$ at time t_k and virtual marker θ_i.

First, we propose the following two sums of squared residuals

$$\begin{aligned} S &:= \sum_{k,i\colon \text{LM}_{k,i}<0} r_{k,i}^2, \\ S^+ &:= \sum_{k,i\colon \text{LM}_{k,i}<0} \left(\min\left(r_{k,i}, 0\right)\right)^2. \end{aligned}$$

We are now interested in the pair of retraction weights $w_{\text{retr}} = (w_{\text{APCSF}}, w_{\text{AAF}}) \in \mathbb{R}^2$ which minimizes one of the above sums

$$\min_{w_{\text{retr}}\in\mathbb{R}^2} S \quad \text{or} \quad \min_{w_{\text{retr}}\in\mathbb{R}^2} S^+.$$

While the first case enforces ideally small corrections $r_{\text{k,i}}$, the second case favors a positive protrusion component f_{prot}.

Subsequently, after estimating w_{APCSF} and w_{AAF}, we can determine w_{prot} such that the underlying protrusion process

$$X_{\text{prot}}(t_k, \theta_i) = L(t_k)\, \frac{f_{\text{prot}}(t_k, \theta_i)}{w_{\text{prot}}}$$

fulfills $\text{Var}(X_{\text{prot}}) = 1$ with $\text{Var}(\cdot)$ denoting the sample variance. This can be achieved by choosing

$$w_{\text{prot}} = \sqrt{\text{Var}(\tilde{X}_{\text{prot}})},$$

with

$$\tilde{X}_{\text{prot}}(t_k, \theta_i) := L(t_k)\, f_{\text{prot}}(t_k, \theta_i).$$

4 Numerical implementation of contour dynamics models

Here, we describe how a contour at time t_k can be evolved for a short time period of $[t_k, t_{k+1}]$. For an initial contour Γ_k, we start with a (preferably) equidistant set of $M \in \mathbb{N}$ contour grid points.

Further, we introduce the following vectorized notation for the spatial coordinates of these markers:

$$\begin{aligned}
\boldsymbol{\Phi}_k^{(x)} &: [t_k, t_{k+1}] \to \mathbb{R}^M, \qquad t \mapsto \boldsymbol{\Phi}_k^{(x)}(t),\\
\boldsymbol{\Phi}_k^{(y)} &: [t_k, t_{k+1}] \to \mathbb{R}^M, \qquad t \mapsto \boldsymbol{\Phi}_k^{(y)}(t),\\
\mathbf{z}_k &: [t_k, t_{k+1}] \to \mathbb{R}^{2M}, \qquad t \mapsto \mathbf{z}_k(t) := \begin{bmatrix} \boldsymbol{\Phi}_k^{(x)}(t) \\ \boldsymbol{\Phi}_k^{(y)}(t) \end{bmatrix},
\end{aligned}$$

with corresponding center of mass trajectories $\Phi_{\mathrm{CM}}^{(x)}(t), \Phi_{\mathrm{CM}}^{(y)}(t) \in \mathbb{R}$. Moreover, we introduce the following vectorized notations for

- the protrusion process $\mathbf{X}_k^{\mathrm{prot}} \in \mathbb{R}^M$ being constant for the entire time period $[t_k, t_{k+1}]$,
- the contour curvature $\boldsymbol{\kappa}(t) \in \mathbb{R}^M$,
- and the unit normal vector components $\vec{\mathbf{n}}^{(x)}(t), \vec{\mathbf{n}}^{(y)}(t) \in \mathbb{R}^M$.

Then, the vectorized model components $\mathbf{f}, \mathbf{f}_{\mathrm{prot}}, \mathbf{f}_{\mathrm{APCSF}}, \mathbf{f}_{\mathrm{AAF}} : \mathbb{R}^+ \times \mathbb{R}^{2M} \to \mathbb{R}^M$ are given by

$$\begin{aligned}
\mathbf{f}_{\mathrm{prot}}(t, \mathbf{z}_k) &= \frac{w_{\mathrm{prot}}}{L(t)} \mathbf{X}_k^{\mathrm{prot}},\\
\mathbf{f}_{\mathrm{APCSF}}(t, \mathbf{z}_k) &= -\, w_{\mathrm{APCSF}} \left(\boldsymbol{\kappa}(t) - \frac{2\pi}{L(t)} \mathbb{1}_M \right),\\
\mathbf{f}_{\mathrm{AAF}}(t, \mathbf{z}_k) &= -\, w_{\mathrm{AAF}} \frac{A(t) - A_{\mathrm{ref}}}{A_{\mathrm{ref}} \cdot L(t)} \Big(\left(\boldsymbol{\Phi}^{(x)}(t) - \Phi_{\mathrm{CM}}^{(x)}(t) \mathbb{1}_M \right) \circ \vec{\mathbf{n}}^{(x)}(t)\\
&\qquad + \left(\boldsymbol{\Phi}^{(y)}(t) - \Phi_{\mathrm{CM}}^{(y)}(t) \mathbb{1}_M \right) \circ \vec{\mathbf{n}}^{(y)}(t) \Big).
\end{aligned}$$

In this context, $\circ$ denotes the element-wise (so-called Hadamard) product and $\mathbb{1}_M$ the all-ones vector of dimension M. Now, we can rewrite Eqs. (14) and (15) of the main manuscript for a set of M contour grid points

$$\begin{aligned}
\frac{\partial \mathbf{z}_k(t)}{\partial t} &= \begin{bmatrix} \mathbf{f}(t, \mathbf{z}_k(t)) \circ \vec{\mathbf{n}}^{(x)}(t) \\ \mathbf{f}(t, \mathbf{z}_k(t)) \circ \vec{\mathbf{n}}^{(y)}(t) \end{bmatrix} \qquad (4.1)\\
\mathbf{f}(t, \mathbf{z}_k(t)) &= \mathbf{f}_{\mathrm{prot}}(t, \mathbf{z}_k(t)) + \mathbf{f}_{\mathrm{APCSF}}(t, \mathbf{z}_k(t)) + \mathbf{f}_{\mathrm{AAF}}(t, \mathbf{z}_k(t)).
\end{aligned}$$

Given an initial contour Γ_k with equidistant contour grid points with corresponding coordinates $\mathbf{z}_k(t_k) = \left[\mathbf{\Phi}_k^{(x)}(t_k), \mathbf{\Phi}_k^{(y)}(t_k)\right] \in \mathbb{R}^{2M}$, we compute the contour dynamics for the time interval $[t_k, t_{k+1}]$ by solving the initial value problem in Eq (4.1). For this purpose, we use the built-in `LSODA` solver in the Python package `SciPy`. This solver is based on backward differentiation formulas (BDF) – an implicit method developed to solve especially stiff ordinary differential equations, see [4, 5] for more details.

The BDF method of order 1 is better known as implicit Euler method – in our notation given by the formula:

$$\mathbf{z}_k(\tau_{i+1}) = \mathbf{z}_k(\tau_i) + h \cdot \mathbf{f}_k(\tau_{i+1}, \mathbf{z}_k(\tau_{i+1})),$$

with initial time $\tau_0 = t_k$, evaluation times $\tau_i = \tau_0 + ih$ and $\tau_N = t_{k+1}$, step size $h = \frac{\delta t}{N}$, and number of steps $N \in \mathbb{N}$. Whereas, $\mathbf{X}_k^{\text{prot}}$ is set to be constant for the above time integration, the geometric quantities $L(\tau)$, $A(\tau)$, $\boldsymbol{\kappa}(\tau)$, $\vec{\mathbf{n}}(\tau)$, and $\Phi_{\text{CM}}(\tau)$ can be updated for each iteration step $t_k \leq \tau \leq t_{k+1}$ until the coordinates $\mathbf{z}_k(\tau_N) = \mathbf{z}_k(t_{k+1})$ of the consecutive contour Γ_{k+1} are derived.

Finally, we conclude the contour evolution with a contour mapping step, i.e., we compute trajectories of all virtual markers between Γ_k and Γ_{k+1} based on a regularized flow, see Fig 1 and S1 Fig for more details. Afterwards, we proceed with the contour evolution for the next time period $[t_{k+1}, t_{k+2}]$, starting again with a preferably equidistant set of contour grid points.

Regularized flows to counteract VM thinning and clustering. Since the model components from Eq (4.1) are mostly acting in normal direction, thinning and clustering effects of virtual markers are inevitable for longer time periods at the front and rear, respectively. For this reason, we propose to propagate an equidistant set of contour grid points with respect to Eq (4.1) for a short time interval $[t_k, t_{k+1}]$ only. Afterwards, we map the initial contour at time t_k to the next one at time $t_{k+1} = t_k + \delta t$ based on a regularized flow as described in [3]. The extent of the regularization of ϕ_k is controlled by a single parameter $\lambda_{\text{reg}} \geq 0$.

In S1 Fig, we illustrate the two different kinds of marker trajectories: (1) the contour propagation based (green dashed lines) and (2) the contour mapping (blue dashed lines) under which X_{prot} is transported. By comparing both trajectories, we nicely see that a regularization, under which X_{prot} is transported, is required to avoid thinning/clustering effects. Moreover, we use this contour mapping for the underlying spatio-temporal coordinate system of the kymograph descriptions displayed later on. More precisely, horizontal lines of any later shown kymograph describe the corresponding quantity along regularized VM trajectories.

In 1, we summarized the above algorithm in form of a pseudocode.

Algorithm 1: ContourDynamicsModel

Input: $\Gamma_0,\ \delta t > 0,\ \lambda_{\text{reg}} \gg 0,\ \vartheta_{\text{model}} \in \mathbb{R}^4,\ \vartheta_{\text{prot}} \in \mathbb{R}^{n_{\text{prot}}}$
Output: $\Gamma_0, \dots, \Gamma_{K-1}$

/* Generate protrusion process for the entire time span */
$X_0^{\text{prot}}, \dots, X_{K-2}^{\text{prot}} =$ `StochasticProcess`$(\vartheta_{\text{prot}})$;

for $k = 0$ **to** $K-2$ **do**

/* Compute virtual marker distance rate */
VMDR = `VMDistanceRate`(Γ_k);

/* Adjust protrusion process w.r.t VMDR */
$X_k^{\text{prot}} = X_k^{\text{prot}}/\text{VMDR}$;

/* Compute coordinates of equidistant contour grid points */
$p_{\text{init}} =$ `GaussianProcessRegression`(Γ_k);

/* Propagate contour grid points based on model functions */
$p_{\text{end}} =$ `SolveIVP`$(p_{\text{init}}, \delta t, X_k^{\text{prot}}, \vartheta_{\text{model}})$;

/* Compute contour mapping based on regularized flow */
$\Gamma_{k+1} =$ `RegularizedFlow`$(\Gamma_k,\ p_{\text{end}}, \lambda_{\text{reg}})$;

end

return $\Gamma_0, \dots, \Gamma_{K-1}$

Figure 1. Algorithm to perform contour dynamics model. The algorithm input consists of an initial contour Γ_0, a step width $\delta t > 0$, a regularization parameter λ_{reg}, the model parameters $\vartheta_{\text{model}} = \{w_{\text{prot}}, w_{\text{APCSF}}, w_{\text{AAF}}, A_{\text{ref}}\}$, and a set of n_{prot} parameters regarding the stochastic protrusion process denoted with $\vartheta_{\text{prot}} \in \mathbb{R}^{n_{\text{prot}}}$. The output contains the artificial cell track based on consecutive contours $\Gamma_0, \dots, \Gamma_K - 1$.

5 Model equations with relative weights

In Eq (16) of the main article, our model is notated with absolute weights $w_{\text{prot}}, w_{\text{APCSF}}, w_{\text{AAF}} > 0$. Alternatively, with an overall velocity parameter

$$w_f = w_{\text{prot}} + w_{\text{APCSF}} + w_{\text{AAF}} \cdot 1\mu m$$

our model can be formulated with respect to relative weights:

$$\begin{aligned} w_{\text{prot}}^{\text{rel}} &= \frac{w_{\text{prot}}}{w_f}, \\ w_{\text{APCSF}}^{\text{rel}} &= \frac{w_{\text{APCSF}}}{w_f}, \\ w_{\text{AAF}}^{\text{rel}} &= \frac{w_{\text{AAF}} \cdot 1\mu m}{w_f} = (1 - w_{\text{prot}}^{\text{rel}} - w_{\text{APCSF}}^{\text{rel}}). \end{aligned} \tag{5.1}$$

Then, we can rewrite Eqs (15) and (16) of the main article as

$$f = w_f \cdot (f_{\text{prot}} + f_{\text{APCSF}} + f_{\text{AAF}}),$$

with the following three components:

$$I: \qquad f_{\text{prot}}(t,\theta) = w^{\text{rel}}_{\text{prot}} \frac{X_{\text{prot}}(t,\theta)}{L(t)},$$

$$II: \qquad f_{\text{APCSF}}(t,\theta) = w^{\text{rel}}_{\text{APCSF}} \left(\frac{2\pi}{L(t)} - \kappa(t,\theta) \right),$$

$$III: \qquad f_{\text{AAF}}(t,\theta) = \left(1 - w^{\text{rel}}_{\text{prot}} - w^{\text{rel}}_{\text{APCSF}}\right) \frac{A(t) - A_{\text{ref}}}{A_{\text{ref}} \cdot L(t)} \left\langle \Phi(t,\theta) - \Phi_{\text{CM}}(t), \vec{n}(t,\theta) \right\rangle.$$

Impact of *a priori* parameter choices on the analysis of experimental data. The F-actin density near the membrane is often interpreted as a marker of protrusive activity. Thus, we would expect some correlation between the F-actin density and the protrusion component in our model. Since the latter is influenced by the choice of the model weights of the two other model components AAF and APCSF, the question arises to what extent the correlation depends on the *a priori* choices of w_{AAF} and w_{APCSF}. In this context, we will use the model equations based on the relative weights described above.

In S13 Fig, we display protrusion component kymographs for varying parameters $w^{\text{rel}}_{\text{prot}} \in \{0.05, 0.5, 0.8\}$, $w^{\text{rel}}_{\text{APCSF}} \in \{0.01, 0.05, 0.1\}$, and $w_f \in \{1, 5, 10, 20\}$ of the cell track from Fig 7 of the main article and the corresponding Pearson correlation coefficient between these kymographs and the fluorescence intensity/F-actin density. For a relatively strong APCSF, i.e. $w^{\text{rel}}_{\text{APCSF}} > 0.1$, we observed distinct positive and negative horizontal patches induced by the contour curvature. On the other hand, we obtained predominantly positive values in the kymograph for a strong AAF, e.g., the top left kymograph for $w_f = 20\mu m/s$. Since the AAF affects/shrinks every part of the contour, the counteracting protrusion component is increased to the same amount for the entire contour in order to replicate the given contour dynamics. In the case of weak AAF, we observed distinct negative (blue) regions in the protrusion component kymograph, indicating that additional retractive forces are necessary to replicate the cell track.

By comparing these protrusion component kymographs with the local motion and the fluorescence intensity displayed in panels (C) and (D) from Fig 7 of the main article, we observed the following similarities and differences. In this context, we focus on the two examples highlighted as black and white dashed boxes in each kymograph. The first example ($300s < t < 400s$) clearly indicates a retraction as shown in the local motion kymograph. However, a significant remaining density of F-actin is seen in the fluorescence intensity kymograph. This pattern is also displayed for most of the protrusion

component kymographs in S13 Fig, e.g., the top left kymograph on the second page ($w_f = 5\mu m/s$, $w_{\text{prot}}^{\text{rel}} = 0.05$, $w_{\text{APCSF}}^{\text{rel}} = 0.01$). This means that our model predicts a significant amount of f_{prot} necessary to slow down the retraction such that the modeled contour dynamics resemble the experimental data. Without this contribution of the protrusion component, the APCSF and AAF would enforce an even stronger retraction. A similar relation can be observed in the second example ($t > 400s$) but to a lesser extent as in the first example. For all cases, the resulting Pearson correlation coefficients between the protrusion component and F-actin density fell within a range of $[0.32, 0.49]$. The best fit with a correlation of $\rho = 0.49$ was obtained for two parameter choices of $\{w_f, w_{\text{prot}}^{\text{rel}}, w_{\text{APCSF}}^{\text{rel}}\}$ with distinctly different values, e.g., $\{10, 0.5, 0.01\}$, and $\{20, 0.8, 0.01\}$. In the case of estimated (relative) model weights used in Fig 7 of the main article, given by $\{10.2, 0.65, 0.01\}$, we achieved a comparable correlation coefficient of $\rho = 0.47$. Since the Pearson correlation coefficient is invariant under changes in location and scale, we conclude that most of the protrusion kymographs displayed in S13 Fig differ in magnitude primarily. However, if the APCSF is chosen too strong ($w_{\text{APCSF}}^{\text{rel}} \geq 0.1$), the resulting protrusion kymographs show substantial differences and a decreasing correlation coefficient. For this reason, the APCSF weight should be chosen relatively low $w_{\text{APCSF}}^{\text{rel}} \leq 0.05$.

In general, we observed that the protrusion component inferred by the model is correlated to the underlying F-actin density. While the protrusion component is affected significantly by the parameter choice, the impact on the above correlation coefficient is minor.

6 Ornstein-Uhlenbeck process as protrusion process

In this section, we define the protrusion process in our model as diffusion process, more precisely as an Ornstein-Uhlenbeck process which is denoted by

$$X : [0, T] \times [0, 2\pi) \to \mathbb{R}, \quad (t, \theta) \mapsto X(t, \theta).$$

Further, we introduce the notation for discrete time steps

$$X(k\delta t, \theta) = X(t_k, \theta) = X_k(\theta),$$

with $X(0, \theta) = X_0(\theta) = 0$ for all $\theta \in [0, 2\pi)$.

In the following, we introduce several functions underlying our Ornstein-Uhlenbeck process. Afterwards, we present the exact definition of X.

Scaling functions. An Ornstein-Uhlenbeck process consists of a mean reversion term, preventing the process from getting too small or too large, and a diffusion term, resulting in process changes due to stochastic innovations. For both terms, we introduce the scaling functions respectively:

- mean reversion rate function:

$$a : [0,T] \times [0,2\pi) \to \mathbb{R}^+ \text{ with } a_k(\theta) \coloneqq a(t_k,\theta), \tag{6.1}$$

- diffusion rate function:

$$b : [0,T] \times [0,2\pi) \to \mathbb{R}^+ \text{ with } b_k(\theta) \coloneqq b(t_k,\theta). \tag{6.2}$$

Innovation function. As underlying correlation function, from which we draw innovations, we use the normalized Poisson kernel:

$$\tilde{k}_r(\theta,\theta') = \frac{k_r(\theta,\theta')}{\sqrt{k_r(\theta,\theta)k_r(\theta',\theta')}} = \frac{(1-r)^2}{1-2r\ \cos(\theta-\theta')+r^2} \tag{6.3}$$

with $\theta,\theta' \in [0,2\pi),\ r \in [0,1)$. It holds the following properties: $\left(\frac{1-r}{1+r}\right)^2 \leq \tilde{k}_r(\theta,\theta') < 1$ for all $\theta \neq \theta'$ and $\tilde{k}_r(\theta,\theta') = 1$ if and only if $\theta = \theta'$. In S2 Fig, the Poisson kernel function from Eq (1) and its normalized version from Eq (6.3) are shown for different parameters $r \in [0,1)$.

Then, the innovations of the Ornstein-Uhlenbeck process are denoted by η and are realized by the following Gaussian process:

$$\begin{aligned}\eta : [0,T] \times [0,2\pi) &\to \mathbb{R},\\ (t,\theta) &\mapsto \eta_t(\theta),\\ (\eta)_t &\sim \mathcal{GP}(0,\tilde{k}_{r_{\text{inn}}}(\cdot,\cdot)),\end{aligned}$$

with zero mean and covariance function $\tilde{k}_{r_{\text{inn}}}(\cdot,\cdot)$ from Eq (6.3) and innovation bandwidth parameter $r_{\text{inn}} \in (0,1)$. For the discrete-time case, we define similarly

$$\eta_k(\theta) \coloneqq \eta(t_k,\theta). \tag{6.4}$$

By choosing a normalized Poisson kernel as covariance function, we obtain spatially correlated noise, while not affecting the overall variance of X_k. In this context, we use the property that $\tilde{k}_{\text{inn}}(\theta,\theta) = 1$ for all $\theta \in [0,2\pi)$.

Discrete formulation. Now, we present a time-discrete formula in order to generate an Ornstein-Uhlenbeck process containing the functions from Eqs (6.1), (6.2), and (6.4). Again, we choose a Lagrangian reference frame to describe the process X_k. For a virtual marker labeled by an initial (normalized) arc length coordinate $\theta \in [0,2\pi)$, the evolution of the protrusion process along this virtual marker is given by $X_k(\theta)$ and starts at $X_0(\theta) = 0$. More precisely, X_k is defined iteratively by the following Ornstein-Uhlenbeck process:

$$X_{k+1}(\theta) = X_k(\theta) + \delta t \cdot \Delta X_k(\theta), \tag{6.5}$$

$$\Delta X_k(\theta) = -a_k(\theta) \cdot X_k(\theta) + \sqrt{2a_k(\theta)} \cdot b_k(\theta) \cdot \eta_k(\theta).$$

In the following, we will use a simplified version with constant rates $a_k(\theta) = a \in \mathbb{R}^+$ and $b_k(\theta) = b \in \mathbb{R}^+$:

$$\Delta X_k(\theta) = -a \cdot X_k(\theta) + \sqrt{2a} \cdot b \cdot \eta_k(\theta). \tag{6.6}$$

Since the magnitude of X_k can be also adjusted by the protrusion weight w_{prot} later on, we recommend to set $b = 1$. This way, and by choosing the normalized Poisson kernel as covariance function of η_k, we obtain $X_k \sim \mathcal{N}(0, 1)$.

Polarization function. A cell polarization can be easily added to the model. This polarization can be induced, e.g., by an extracellular nutrient gradient or by a "leading process" defining the general tendency of the cell to move persistently in one direction. We assume that the polarization of the cell is time-dependent and defined by a singular location on the cell contour representing the mid of the cell's front. From these assumptions, we introduce the following function

$$\theta_{\text{pol}} : [0, T] \to [0, 2\pi), \quad t \mapsto \theta_{\text{pol}}(t).$$

A simple test case for a constant polarization would be to choose $\theta_{\text{pol}}(\cdot) = \pi$ as we did for Hawkes processes in the main manuscript.

Further, we define the polarization function $p(t, \theta)$ as the following mapping:

$$\begin{aligned} p : [0, T] \times [0, 2\pi) \to \mathbb{R}^+ \text{ with} \\ (t, \theta) \mapsto \tilde{k}_{r_{\text{pol}}}(\theta, \theta_{\text{pol}}(t)), \end{aligned}$$

with normalized Poisson kernel $\tilde{k}_{r_{\text{pol}}}$ defined as in Eq (6.3) with corresponding polarization parameter $r_{\text{pol}} \in [0, 1)$. The polarization function fulfills the property $\left(\frac{1-r_{\text{pol}}}{1+r_{\text{pol}}}\right)^2 \leq p_k(\theta) \leq 1$ and reaches its maximum $p_k(\theta) = 1$ if and only if $\theta = \theta_{\text{pol}}(t_k)$, for more details see S2 Fig.

For the case $r_{\text{pol}} = 0$ and any function $\theta_{\text{pol}} : [0, T] \to [0, 2\pi)$, the polarization function simplifies to

$$p(\cdot, \cdot) = \tilde{k}_0(\cdot, \theta_{\text{pol}}(\cdot)) = 1,$$

i.e., no polarization effect takes place. For the case $r_{\text{pol}} \to 1$, the polarization functions simplifies to

$$p(t, \theta) = \begin{cases} 1, & \theta = \theta_{\text{pol}}(t) \\ 0, & \theta \neq \theta_{\text{pol}}(t) \end{cases}$$

which is equal to the indicator function $\mathbb{1}_{\{\theta_{\text{pol}}(t)\}}(\theta)$. Finally, we introduce a discrete notation:

$$p_k(\theta) := p(t_k, \theta) = \tilde{k}_{r_{\text{pol}}}(\theta, \theta_{\text{pol}}(t_k)). \tag{6.7}$$

Protrusion component. Motivated from the biological insight that protrusions and retractrions are triggered by the underlying actin and myosin concentration, respectively, we designed our model to separate the formation of protrusions from the formation of retractions. However, in order to obtain a separation of protrusions, resulting from the protrusion component, and retractions, resulting from the APCSF and AAF, the process $X(t, \theta)$ should only take positive values. For this reason, we propose the following modifications of the Ornstein-Uhlenbeck process from Eq (6.5):

$$\begin{aligned}
X^{+}_{\text{prot}}(t_k, \theta) &:= \frac{p_k(\theta)}{\text{VMDR}(t_k, \theta)} \cdot X_k(\theta)^{+}, \qquad (6.8)\\
X^{2}_{\text{prot}}(t_k, \theta) &:= \frac{p_k(\theta)}{\text{VMDR}(t_k, \theta)} \cdot X_k(\theta)^{2},\\
X^{\exp}_{\text{prot}}(t_k, \theta) &:= \frac{p_k(\theta)}{\text{VMDR}(t_k, \theta)} \cdot \exp\left(X_k(\theta)\right),\\
X^{\text{lin}}_{\text{prot}}(t_k, \theta) &:= \frac{p_k(\theta)}{\text{VMDR}(t_k, \theta)} \cdot X_k(\theta) + c_{\text{lin}}\\
X^{\log}_{\text{prot}}(t_k, \theta) &:= \frac{p_k(\theta)}{\text{VMDR}(t_k, \theta)} \cdot \frac{1}{1 + \exp\left(-\beta_{\log} X_k(\theta)\right)},
\end{aligned}$$

with $c_{\text{lin}} > 0, \beta_{\log} > 0$, a polarization function p_k defined as in Eq (6.7), and virtual marker distance ratio defined as in Eq (4) in the main article. For the linear shift modification, $c_{\text{lin}} > 0$ needs to be sufficiently large in order to obtain mostly positive values for $X^{\text{lin}}_{\text{prot}}$.

Simulating amoeboid motility by an Ornstein-Uhlenbeck process

In the main manuscript, we have shown that amoeboid cell motility can be well simulated with a self-exciting Poisson point process, as so-called Hawkes process. In this section, we simulate contour dynamics driven by an Ornstein-Uhlenbeck process (OUP) defined as in Eq (6.6). The OUP is commonly applied in other cell motility models where it is often used to model changes of two-dimensional bio marker concentrations. For this reason, we investigated if the OUP is also suitable within our model. In the above section, we introduced multiple modifications of the OUP, necessary to separate the formation of protrusions from the formation of retractions. We therefore studied the influence of each modification on the overall contour dynamics.

First, we generated cell tracks based on the OUP in Eq (6.6) for all modifications mentioned above. The mean reversion rate and diffusion rate were set to be constant: $a = 0.05$ and $b = 1$. Furthermore, we assumed no polarization, i.e., $r_{\text{pol}} = 0$. Since the modifications from Eq (6.8) affect the magnitude of the protrusion process in different ways, the protrusion

weight w_{prot} needed to be adjusted for each modification. For this reason, we have chosen $w_{\text{prot}} = 15; 5; 5; 7.5; 20\mu m/s$ for X^{+}_{prot}, X^{2}_{prot}, $X^{\exp}_{\text{prot}}$, $X^{\text{lin}}_{\text{prot}}$, and $X^{\log}_{\text{prot}}$, respectively. In general, the reference area of A_{ref} acts as lower bound of the area in our model. From experimental recordings, we can say that $A_{\text{ref}} = 80\mu m^2$ is plausible for an exemplary cell track. The exact choice of all parameters can be found in Table 1. The first group of parameters regarding the contour parametrization and the flow between contours is described in a more detailed way in [3].

Table 1. Choice of parameters and meaning.

Parameter	Value	Unit	Meaning
**Contour parametrization**			
r_{cont}	0.6	–	GPR smoothing
σ_{noise}	0.05	–	GPR noise
λ_{reg}	10	$\frac{\mu m^2}{s^2}$	Flow regularization
A_{ref}	80	μm^2	Reference area
**Ornstein-Uhlenbeck process**			
a	0.05	s^{-1}	Mean reversion rate
b	1	$s^{-\frac{1}{2}}$	Diffusion rate
r_{inn}	0.5	–	Corr. length of innovations
r_{pol}	0	–	Corr. length of polarization
**Model weights**			
w_{prot}	5–20	$\frac{\mu m}{s}$	Protrusion weight
w_{APCSF}	0.1	$\frac{\mu m}{s}$	APCSF weight
w_{AAF}	1	$\frac{\mu m}{s}$	AAF weight

List of parameters for simulated contour dynamics based on an Ornstein-Uhlenbeck process.

In the top row of Fig 2, cell tracks based on the different modifications from Eq (6.8) are shown. For each modification, the same realization of the Ornstein-Uhlenbeck process X_{prot} is underlying. The non-polarized behavior of the cell tracks can be clearly seen. From the covered trace (grey area) of each cell track and its corresponding center of mass trajectory (colored line), we observe different degrees of motility for $X^{\exp}_{\text{prot}}$, X^{+}_{prot}, X^{2}_{prot}, $X^{\text{lin}}_{\text{prot}}$, and $X^{\log}_{\text{prot}}$ (descending order).

Furthermore, the kymographs of the overall local motion f and its three components $f_{\text{prot}}, f_{\text{APCSF}}, f_{\text{AAF}}$ are displayed for each of these cell tracks. For

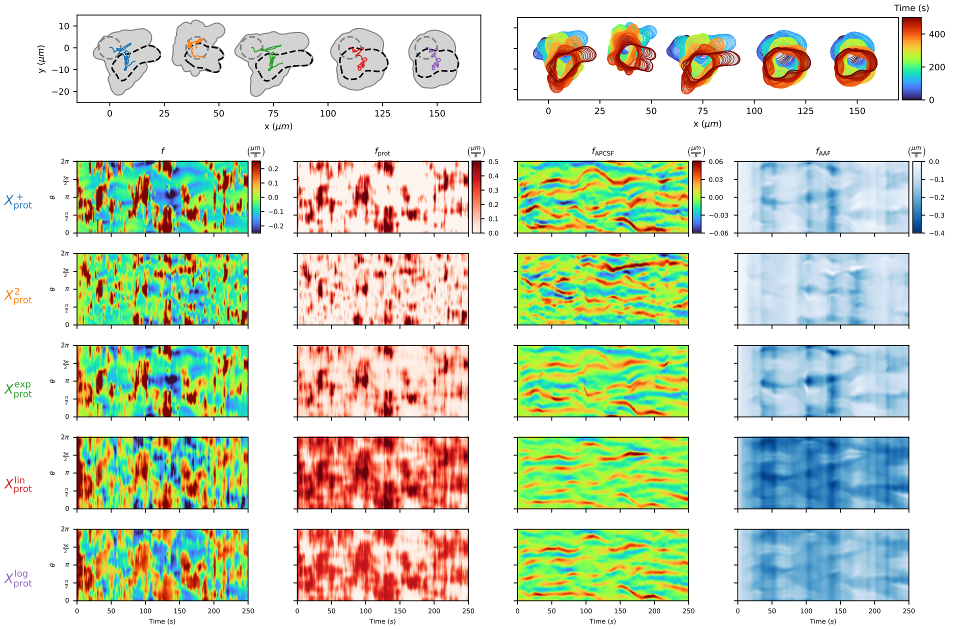

Figure 2. Comparison of cell tracks obtained from different modifications of the underlying Ornstein-Uhlenbeck process. In the first row, the entire area (trace) covered by each cell track is displayed (left, gray area) as well as their center of mass trajectories (left, colored lines) and the evolution of the contour dynamics over a time span of $T = 500s$ (right, colored contours). Below, for each modification, the corresponding kymographs of the following quantities are displayed for the first half of each cell track: the local motion f and its three components f_{prot}, f_{APCSF}, and f_{AAF} (from left to right).

X^+_{prot}, the expanding regions (red, first two columns) are more distinct and with sharp edges directly resulting from the non-smooth behavior of X^+_{prot}. In contrast, the expanding regions of X^2_{prot} are smoother. Furthermore, by taking also the negative parts of X_{prot} into account, the number of expanding regions approximately doubles. For the third case $X^{\text{exp}}_{\text{prot}}$, we observe kymographs similar to X^+_{prot} but with smoother transitions between expansions and retractions. In the fourth case, we have chosen $c_{\text{lin}} = 1.96$ such that $\mathbb{P}(X^{\text{lin}}_{\text{prot}} > 0) = 0.975$. As a consequence of this mean shift, the corresponding protrusion kymograph shows larger expanding regions. Due to a larger protrusion component, the size of the cell contour is significantly increased. Since f_{APCSF} is proportional to the contour curvature, which decreases due to a larger contour size, the corresponding kymograph (3rd column) shows less pronounced curvature patterns. An increased contour area can be also inferred from the f_{AAF} kymograph (4th column), indicated

by a dark blue color, leading to a stronger area adjustment. For the logistic modification $X_{\text{prot}}^{\log}$ with $\beta_{\log} = 2$, a similar effect of an increased contour size can be observed.

In S3 Vid, all five cell tracks are displayed at tenfold speed. In accordance to the kymographs from Fig 2, we observed the non-smooth behavior for X_{prot}^{+} with abrupt changes of the cell contour. For X_{prot}^{2}, we observed more protrusions evolving more smoothly compared to X_{prot}^{+}. However, by creating too many protrusions, canceling each other out, the overall cell motility can be reduced. This effect was also shown in [6]. Since X_{prot}^{2} depends also on the negative regions of the initial Ornstein-Uhlenbeck process, the resulting cell track differs significantly from the other four cell tracks, which are synchronous most of the time. From the exponential modification $X_{\text{prot}}^{\exp}$, a cell track with rapidly generating protrusions was observed. Finally, the cell tracks obtained from $X_{\text{prot}}^{\text{lin}}$ and $X_{\text{prot}}^{\log}$ were almost stationary with an increased cell shape and lesser curvature which coincides with the above observations.

In general, we recommend using the exponential modification $X_{\text{prot}}^{\exp}$ resulting in smooth contour changes driven by fast and more explorative protrusions. However, some protrusions look artificial as well as additional artifacts such as a pulsating membrane and a partially swimming type of locomotion. While this method provides a simple way of generating cell tracks, they can be easily distinguished from experimental cell tracks. Based on the findings of the main article, where we have chosen a Hawkes process as underlying protrusion component, much more realistic contour dynamics were generated. Due to its self-exciting property, the Hawkes process is capable of producing cascades of multiple protrusions with accompanying reorientation phases of the cell. Compared to the OUP, the Hawkes process is therefore the better choice for modeling amoeboid cell motility.

References

[1] Mäder-Baumdicker E. The area preserving curve shortening flow with Neumann free boundary conditions. Geometric Flows. 2015;1(1):1–57. doi:10.1515/geofl-2015-0004.

[2] Mäder-Baumdicker E. Singularities of the area preserving curve shortening flow with a free boundary condition. Mathematische Annalen. 2018;371(3-4):1429–1448. doi:10.1007/s00208-017-1637-9.

[3] Schindler D, Moldenhawer T, Stange M, Lepro V, Beta C, Holschneider M, et al. Analysis of protrusion dynamics in amoeboid cell motility by means of regularized contour flows. PLOS Computational Biology. 2021;17(8):e1009268. doi:10.1371/journal.pcbi.1009268.

[4] Hindmarsh AC. ODEPACK, a systematized collection of ODE solvers. Scientific Computing: Applications of Mathematics and Computing to the Physical Sciences. 1983;1(IMACS Transactions on Scientific Computation):55–64.

[5] Petzold L. Automatic Selection of Methods for Solving Stiff and Nonstiff Systems of Ordinary Differential Equations. SIAM Journal on Scientific and Statistical Computing. 1983;4(1):136–148. doi:10.1137/0904010.

[6] Heck T, Vargas DA, Smeets B, Ramon H, van Liedekerke P, van Oosterwyck H. The role of actin protrusion dynamics in cell migration through a degradable viscoelastic extracellular matrix: Insights from a computational model. PLoS Computational Biology. 2020;16(1):1–34. doi:10.1371/journal.pcbi.1007250.
