# Supplementary material for: Three-component contour dynamics model to simulate and analyze amoeboid cell motility in two dimensions
Source: PLoS One. 2024 Jan 26;19(1):e0297511. doi: 10.1371/journal.pone.0297511 (PMC10817190; doi:10.1371/journal.pone.0297511)
Supplement: S1 Fig — (PDF) [file pone.0297511.s002.pdf]

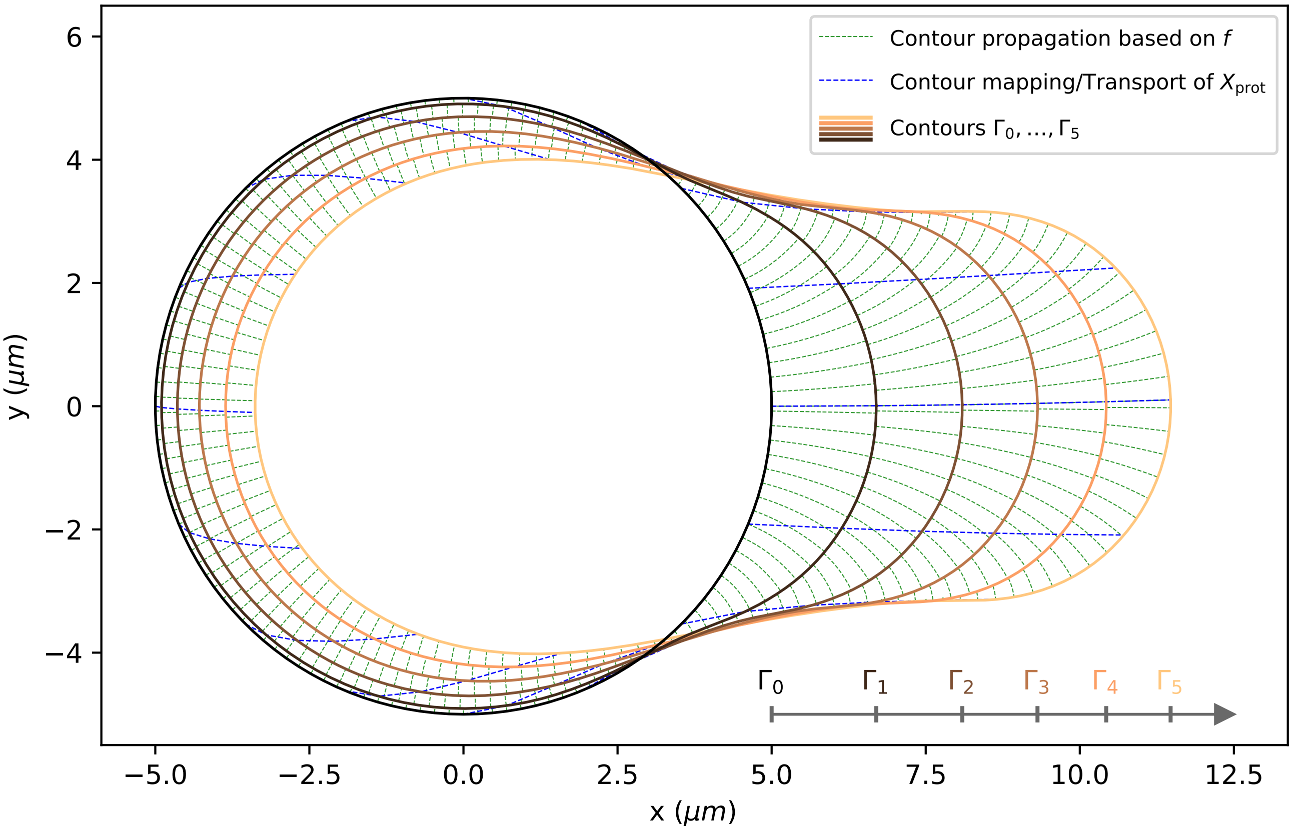

Fig S1. Artificial contour dynamics based on our model with a comparison of the underlying contour propagation and contour mapping. First, the contour is propagated by the model function *f* evaluated for an equidistant set of grid points on the contour (green dashed trajectories). Secondly, the protrusion component X_{prot} is propagated by a strongly regularized flow to avoid thinning and clustering effects of virtual markers (blue dashed trajectories). This contour mapping can then be used as an underlying coordinate system in kymograph representations.
