# Supplementary material for: Three-component contour dynamics model to simulate and analyze amoeboid cell motility in two dimensions
Source: PLoS One. 2024 Jan 26;19(1):e0297511. doi: 10.1371/journal.pone.0297511 (PMC10817190; doi:10.1371/journal.pone.0297511)
Supplement: S2 Fig — (PDF) [file pone.0297511.s003.pdf]

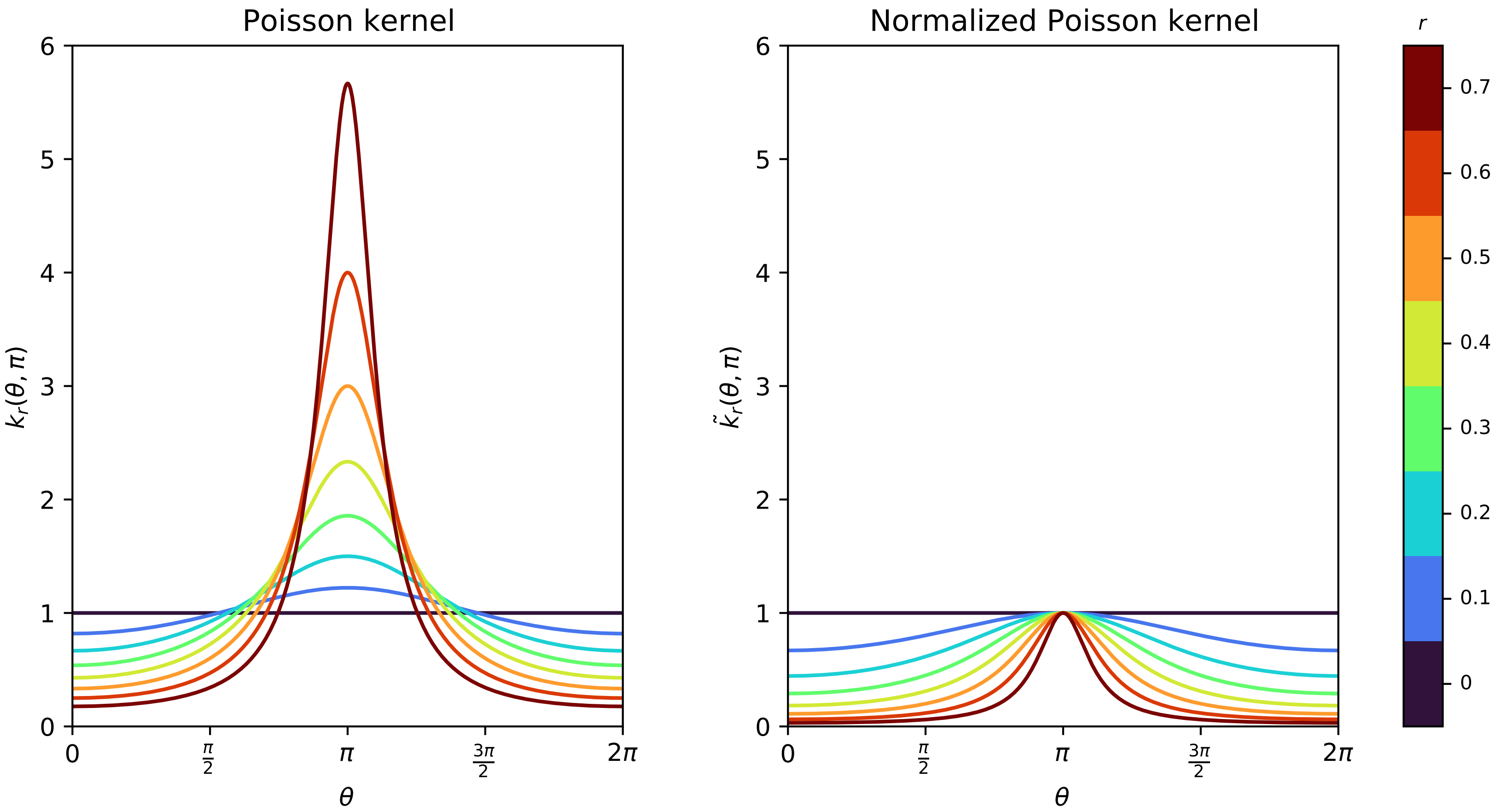

Fig S2. Poisson kernel function k_r (left) and normalized Poisson kernel function $\tilde{k}_r$ (right) for varying radius parameter $r \in \{0, 0.1, \ldots, 0.7\}$.
