# Supplementary material for: Three-component contour dynamics model to simulate and analyze amoeboid cell motility in two dimensions
Source: PLoS One. 2024 Jan 26;19(1):e0297511. doi: 10.1371/journal.pone.0297511 (PMC10817190; doi:10.1371/journal.pone.0297511)
Supplement: S3 Fig — (PDF) [file pone.0297511.s004.pdf]

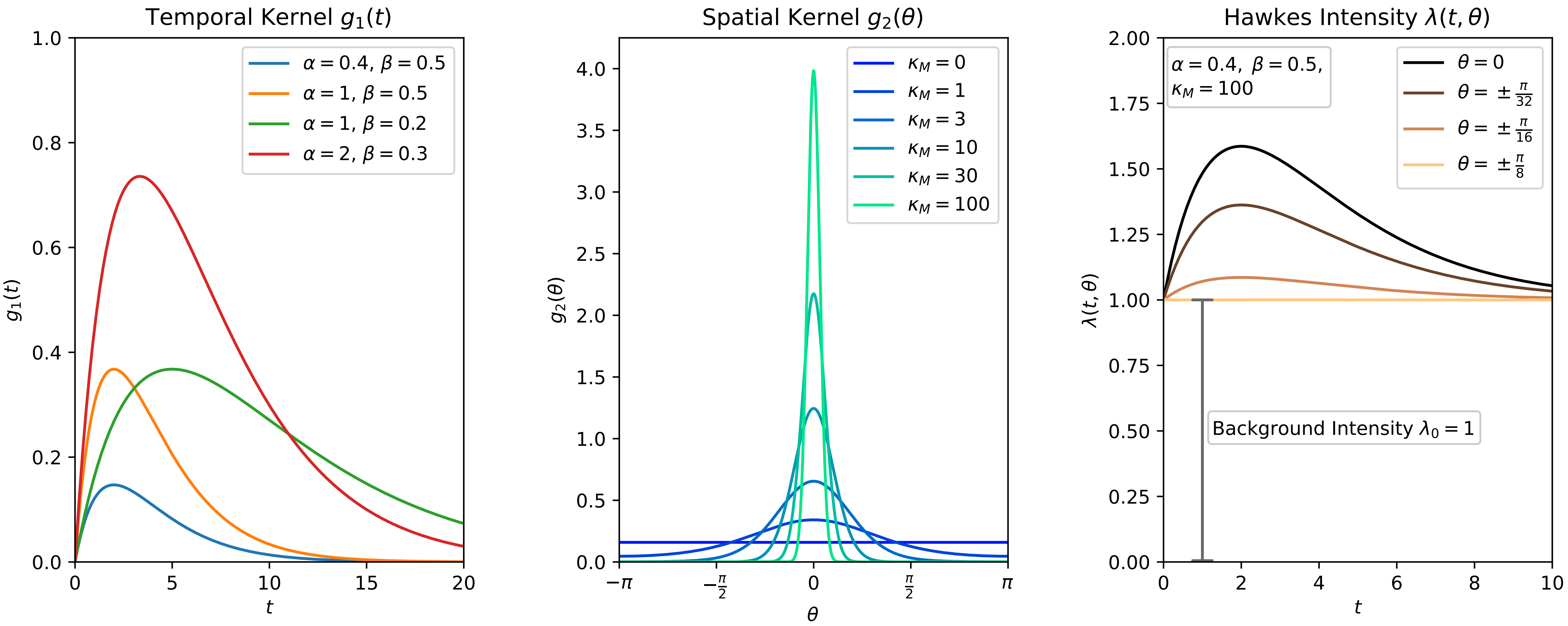

Fig S3. Illustration of kernel functions used to generate artificial cell tracks driven by a Hawkes process. **(Left)** Temporal kernel $g_1(t)$ with varying arrival intensity $\alpha > 0$ and exponential decay rate $\beta > 0$. **(Middle)** Spatial Kernel $g_2(\theta)$ with varying concentration parameter $\kappa_M \geq 0$. **(Right)** Hawkes Intensity $\lambda(t, \theta)$ with background intensity $\lambda_0 = 1$ for varying θ along the cell contour and the following choice of parameters: $\alpha = 0.4$, $\beta = 0.5$, and $\kappa_M = 100$ as used in our model.
