# Supplementary figures and images for: Three-component contour dynamics model to simulate and analyze amoeboid cell motility in two dimensions
Source: PLoS One. 2024 Jan 26;19(1):e0297511. doi: 10.1371/journal.pone.0297511 (PMC10817190; doi:10.1371/journal.pone.0297511)

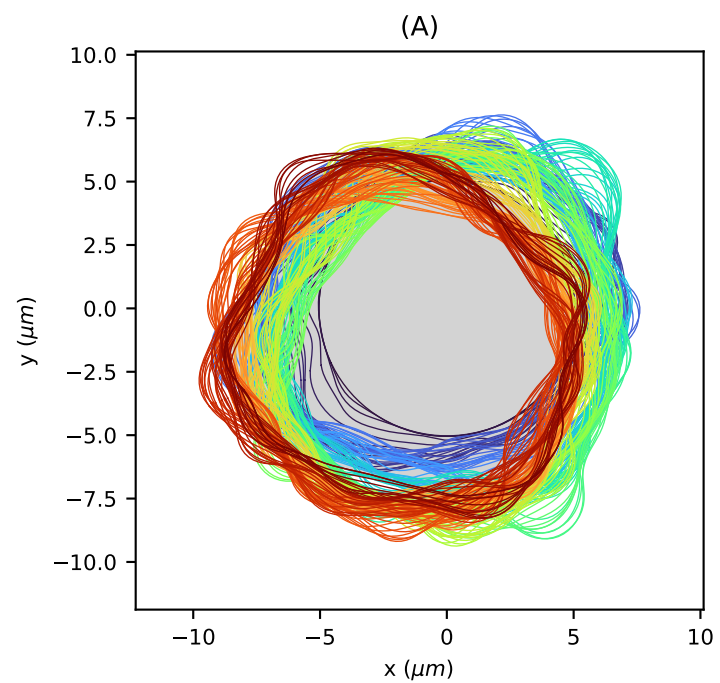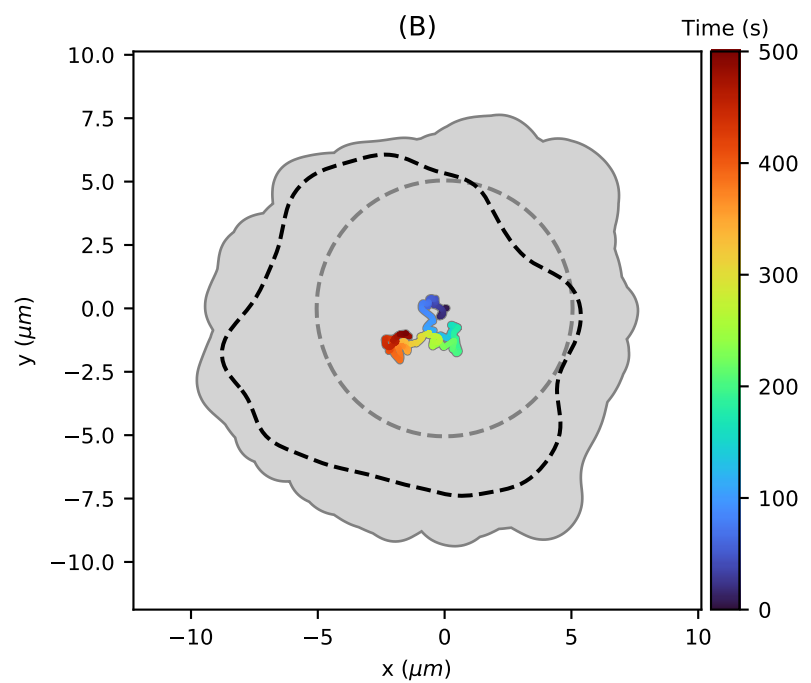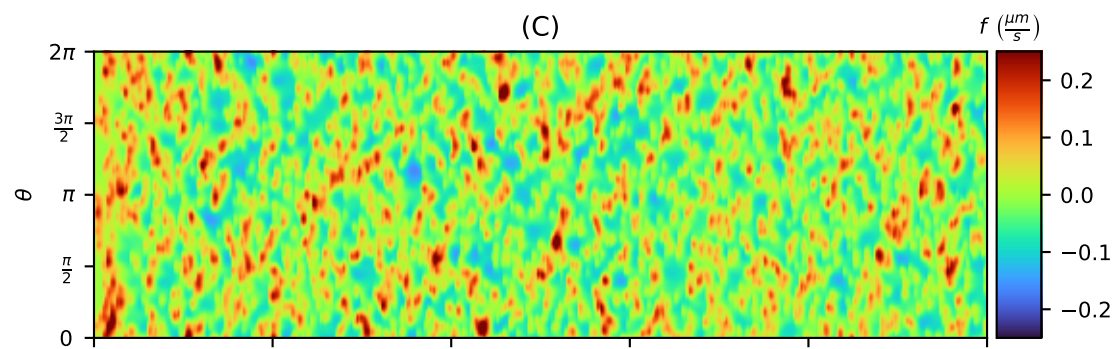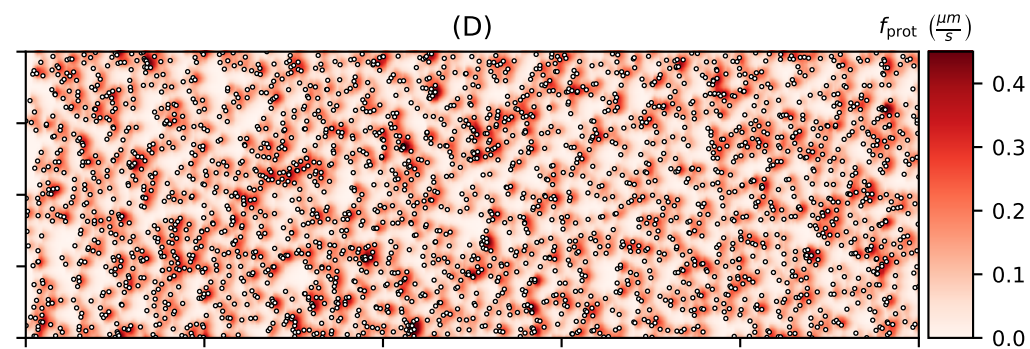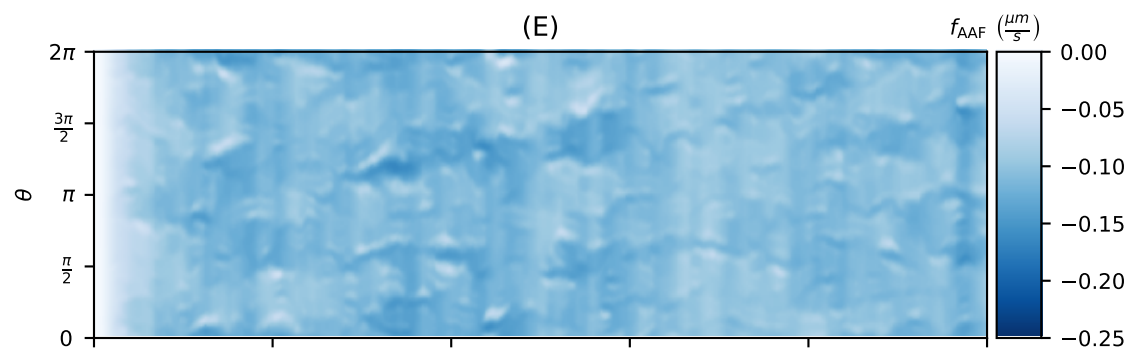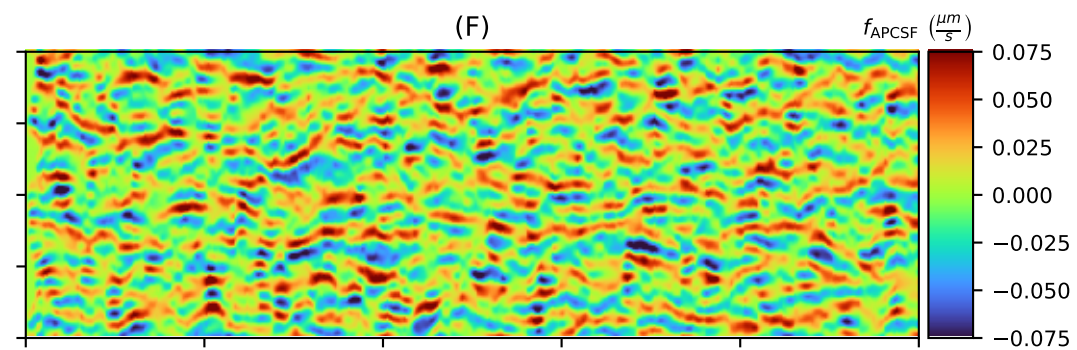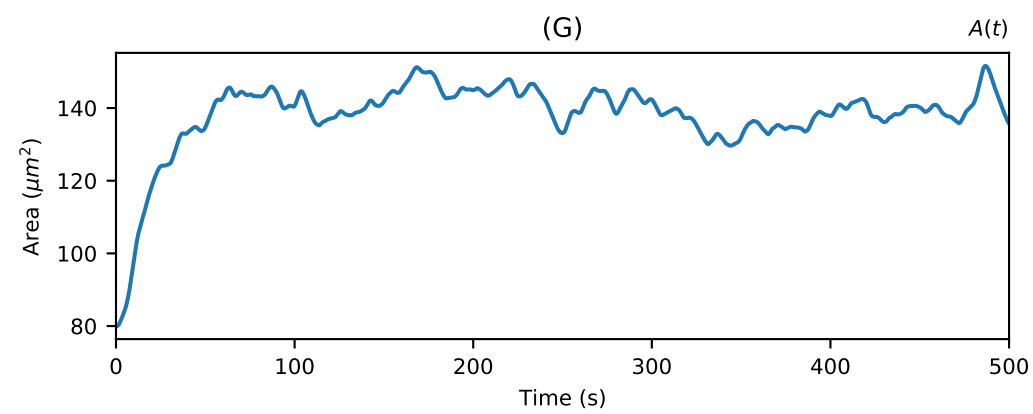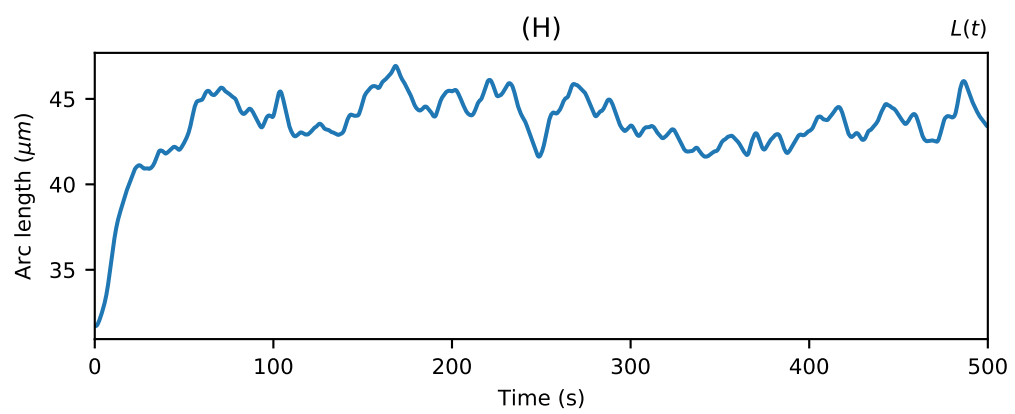

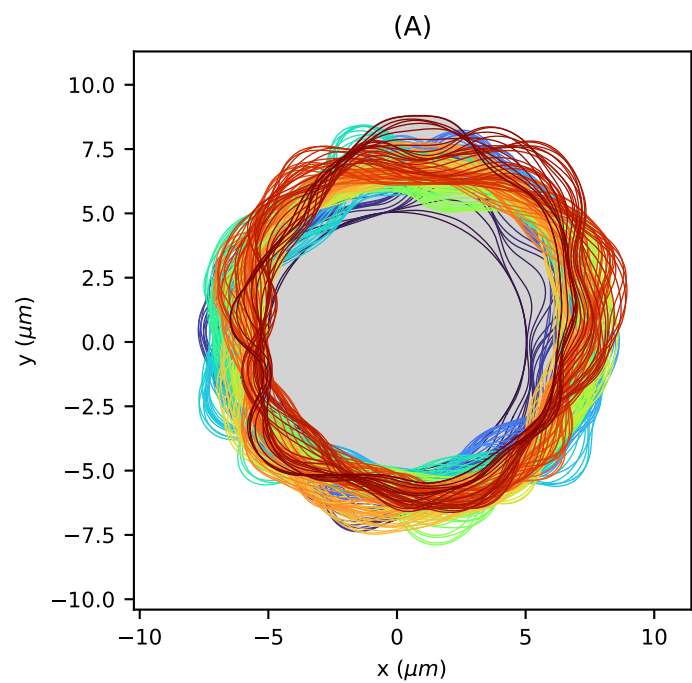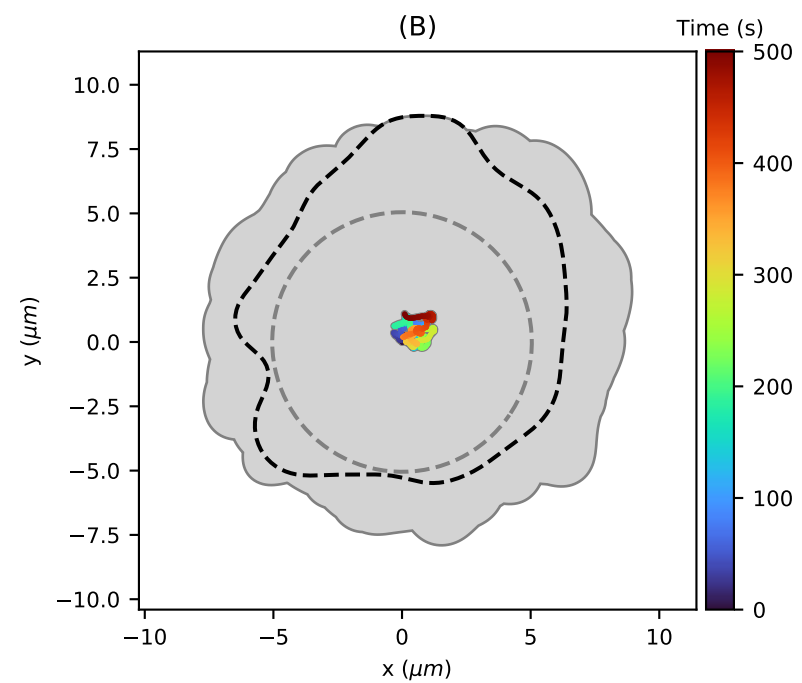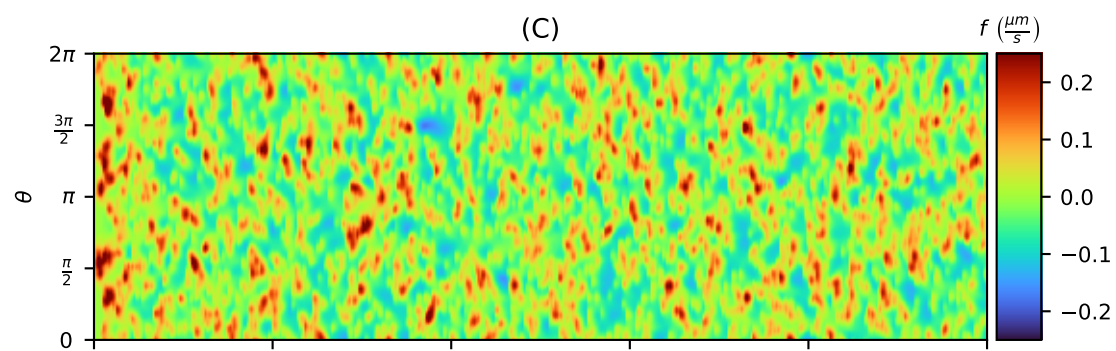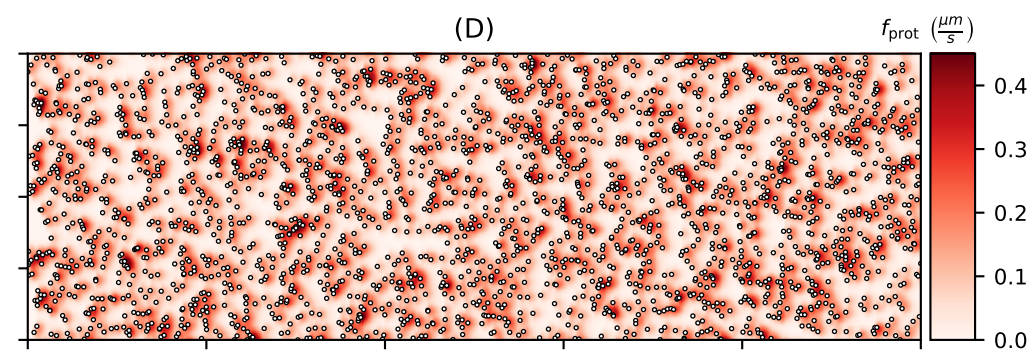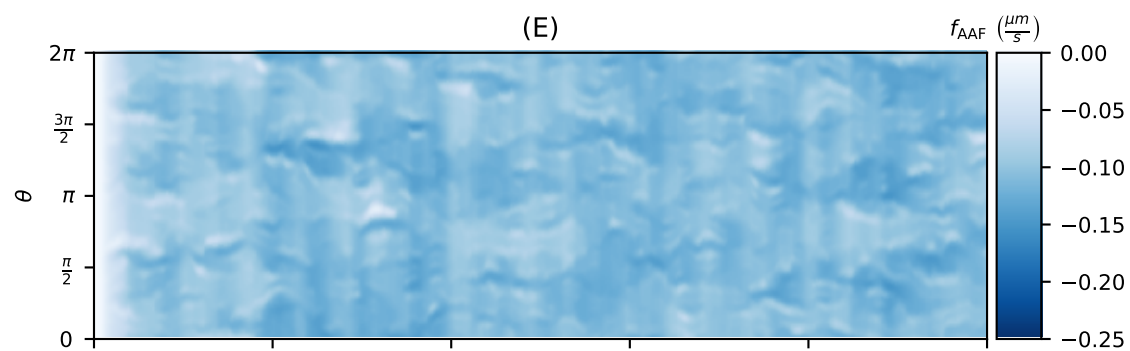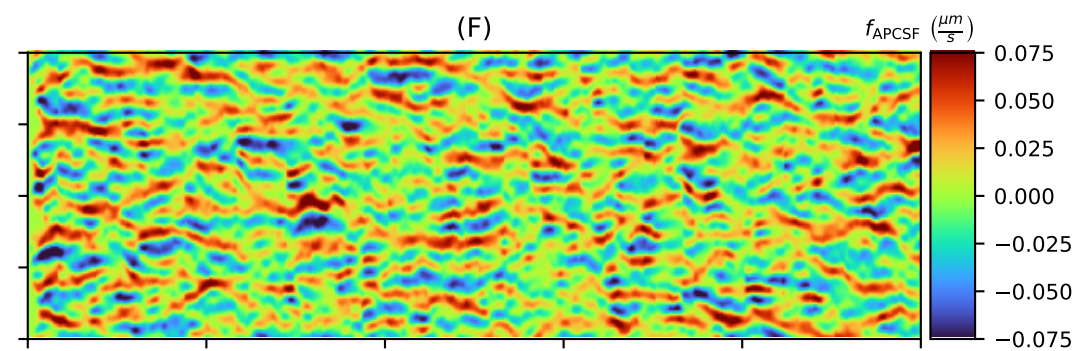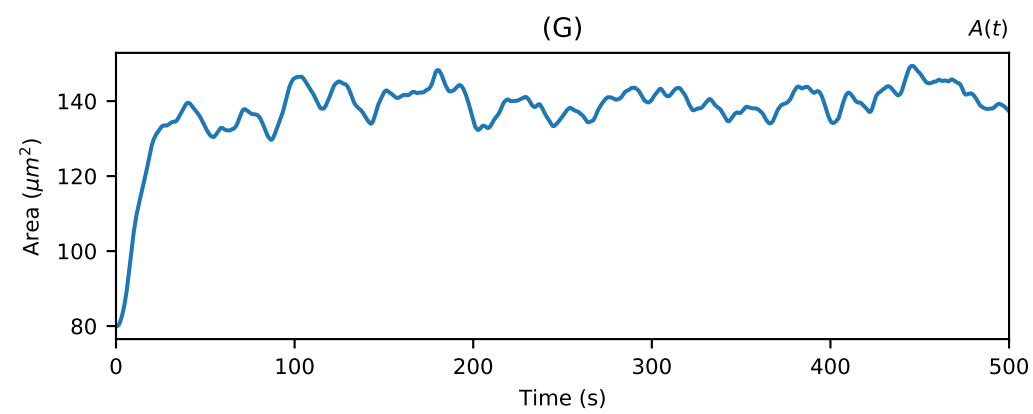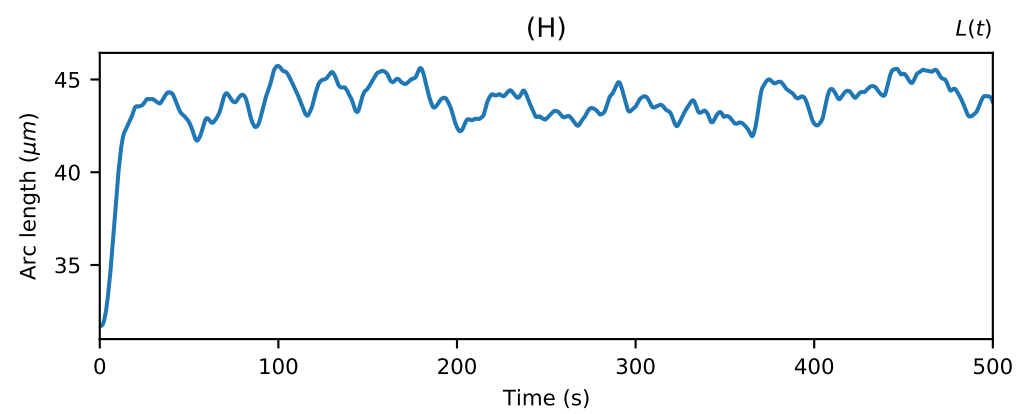

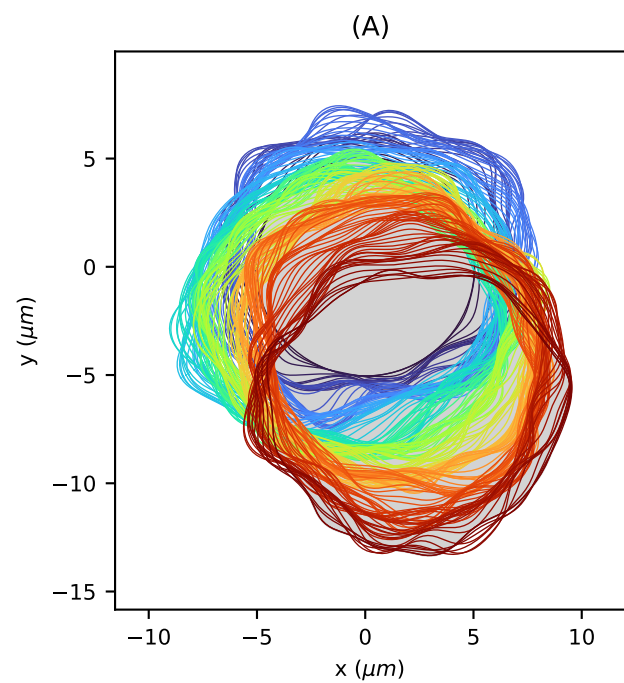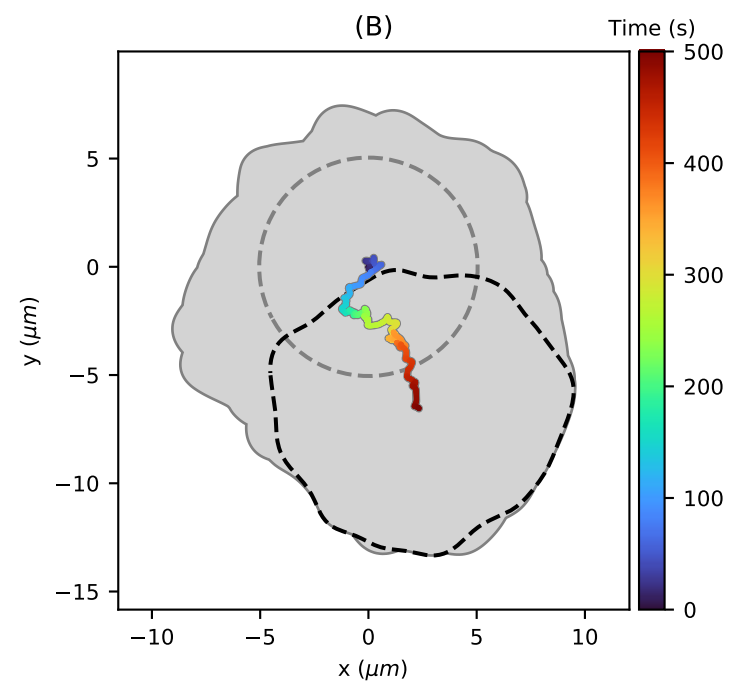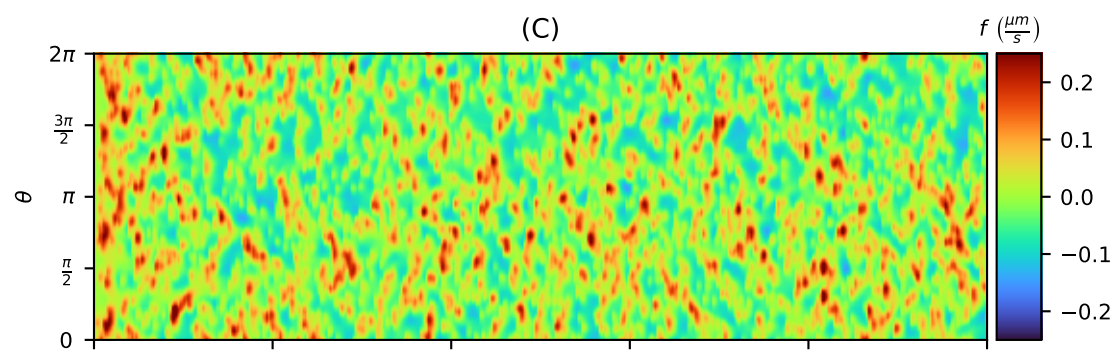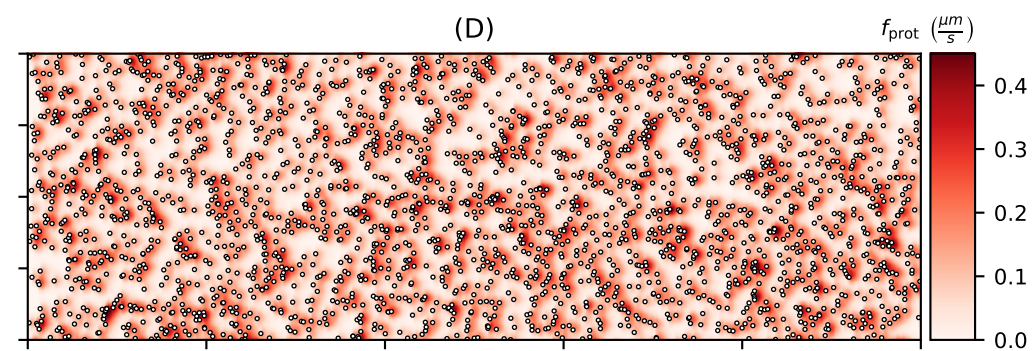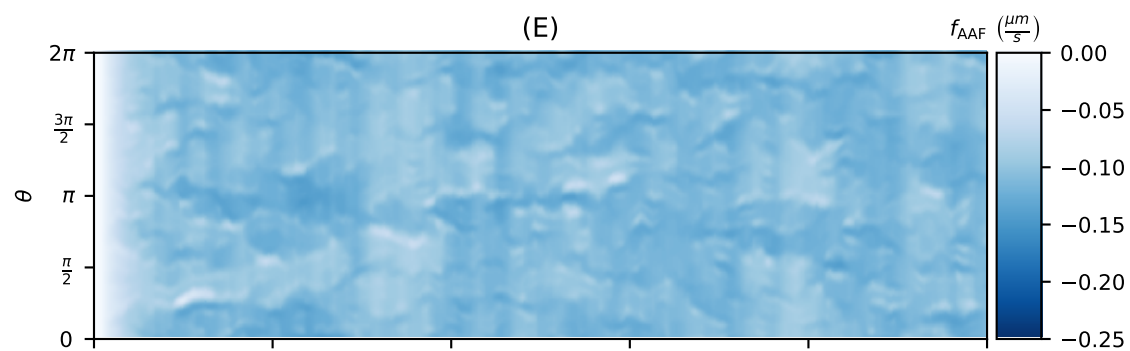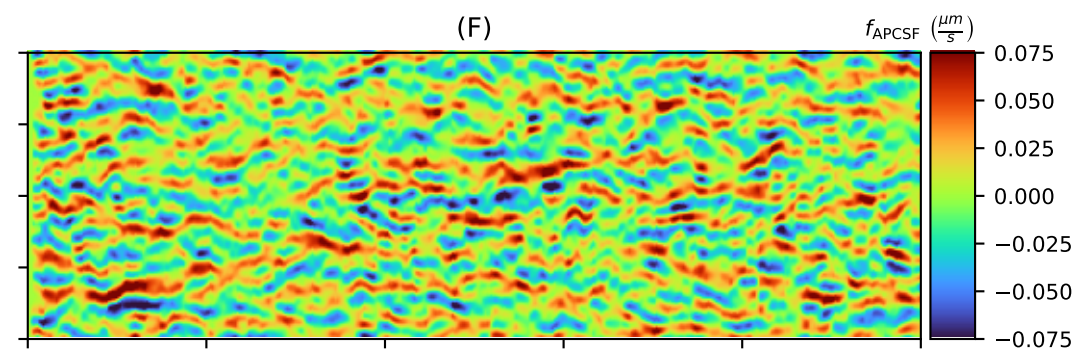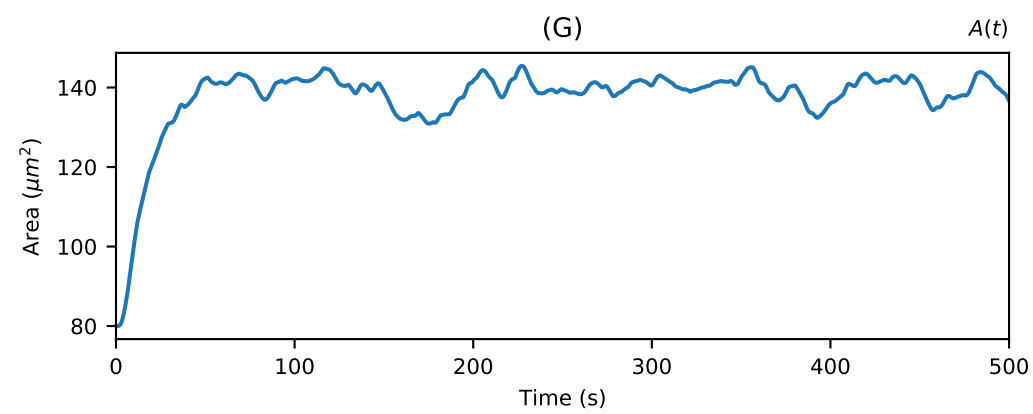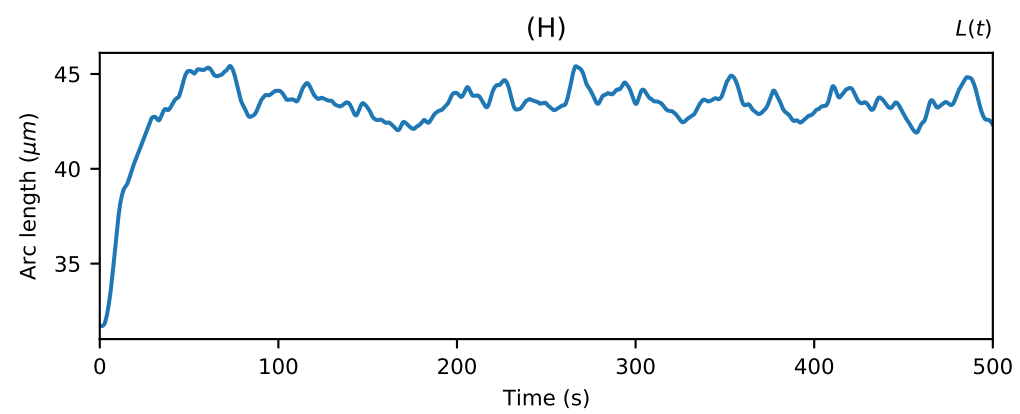

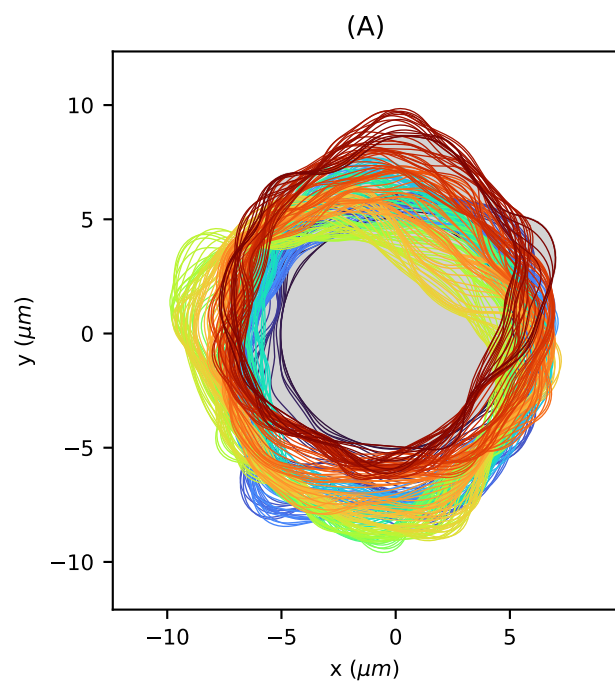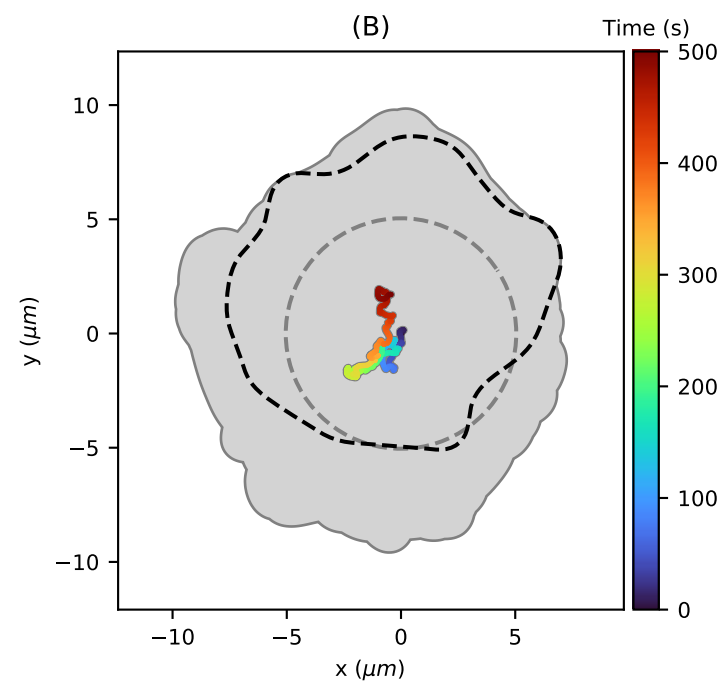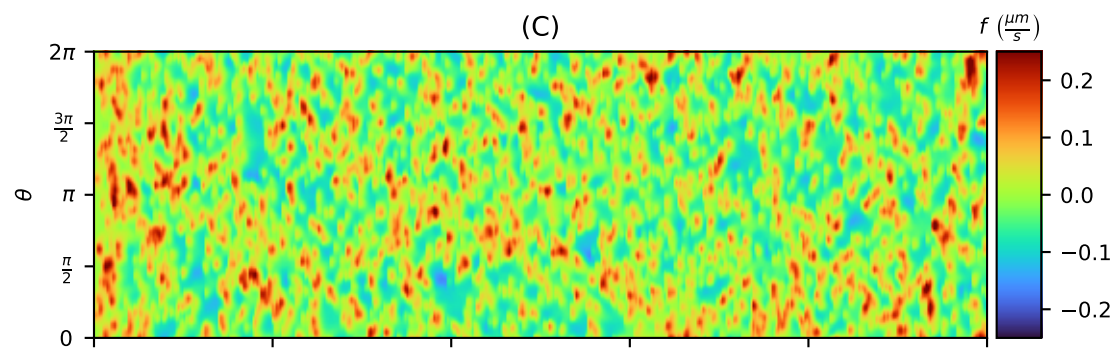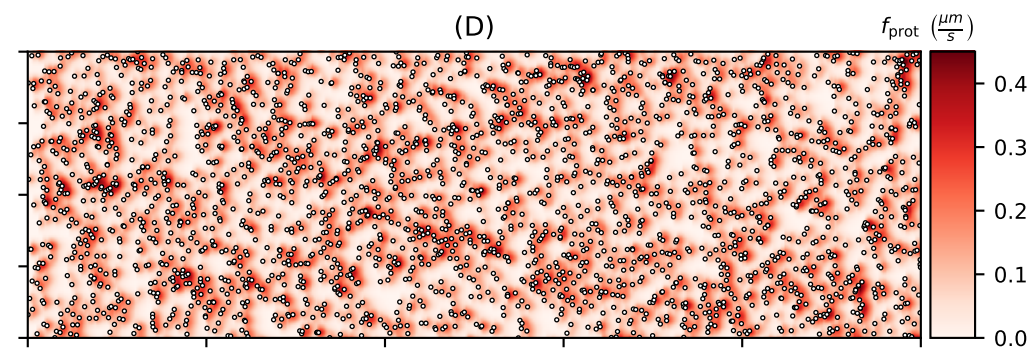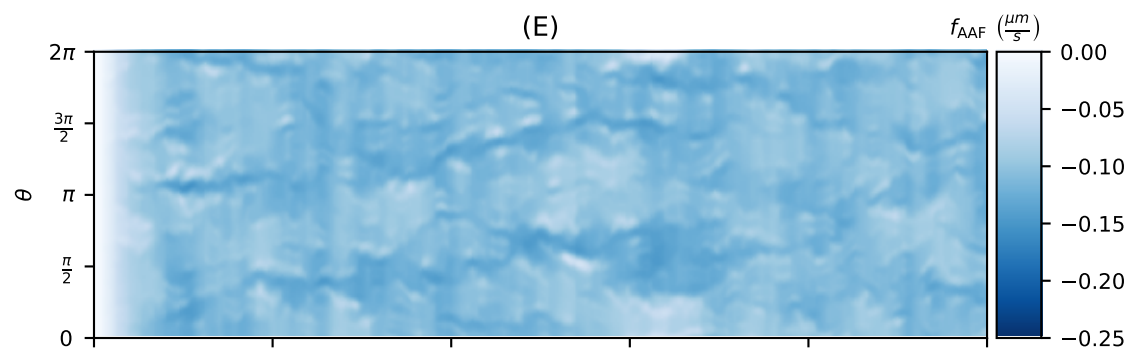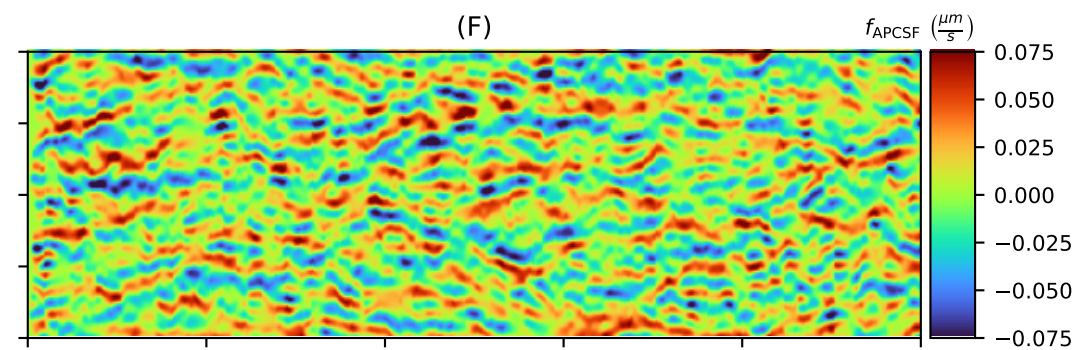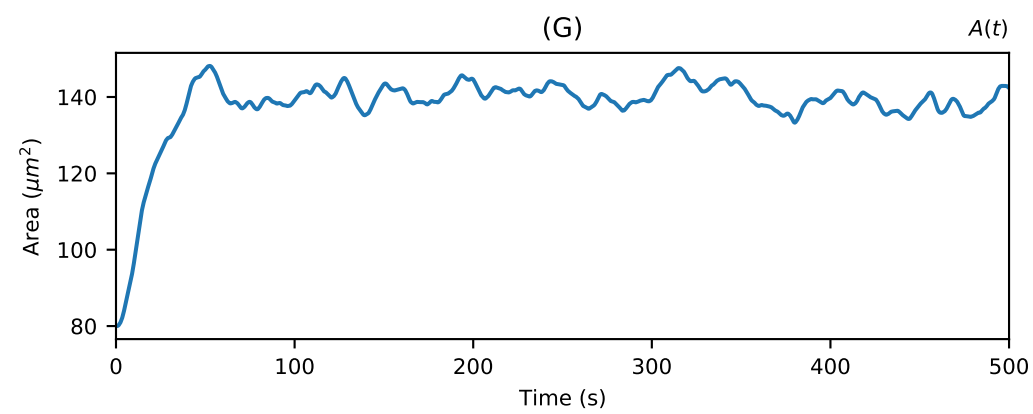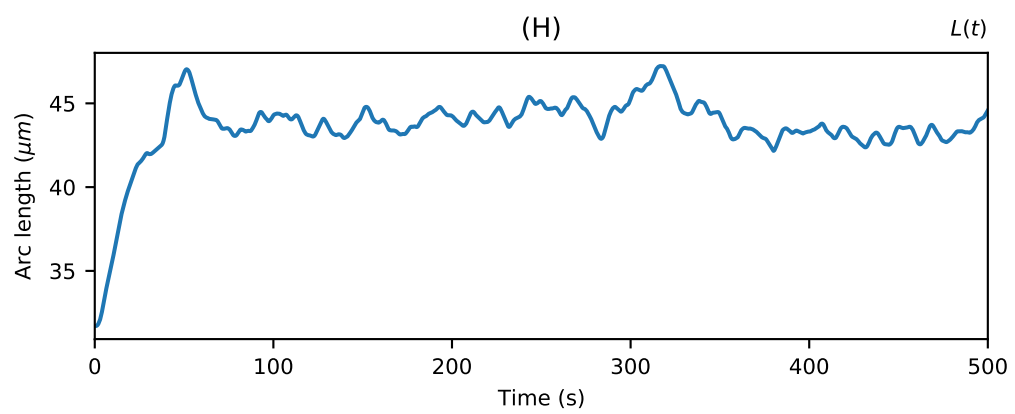

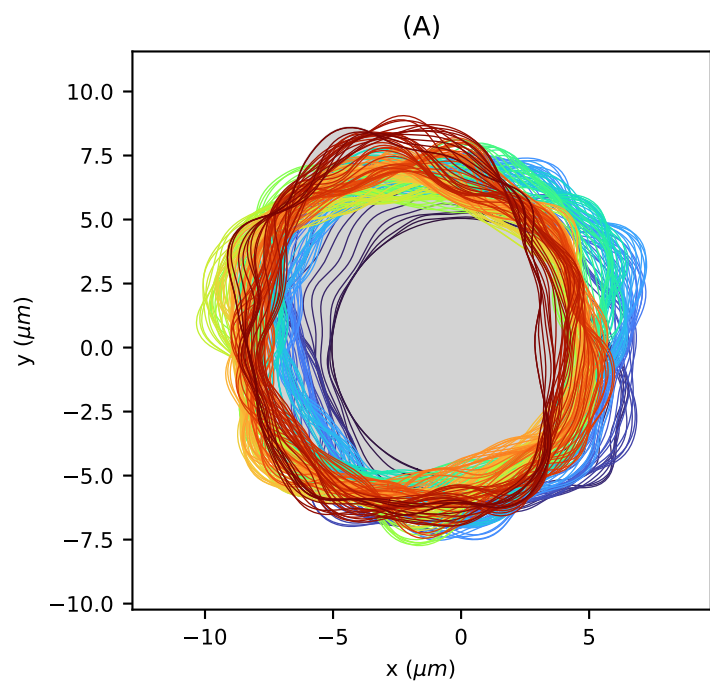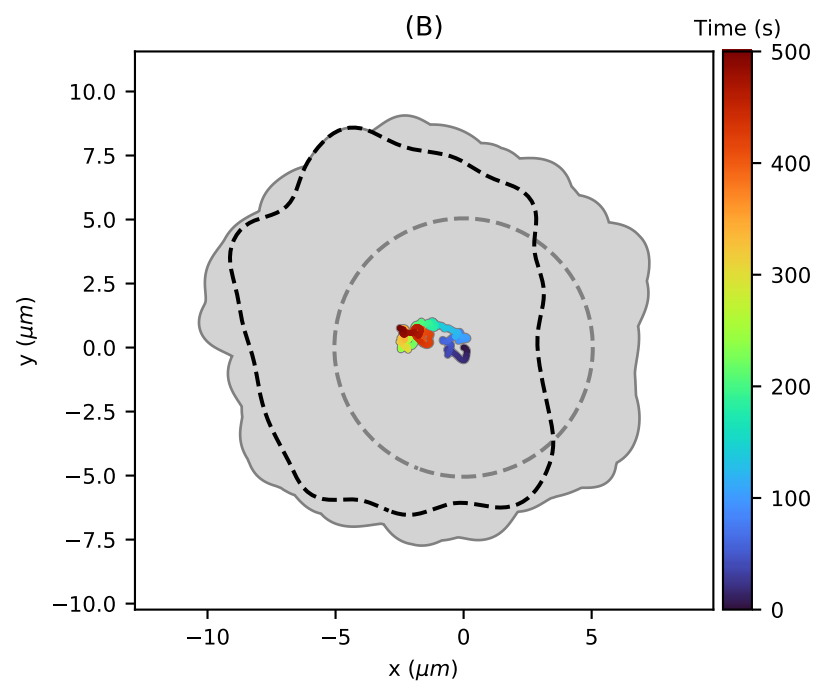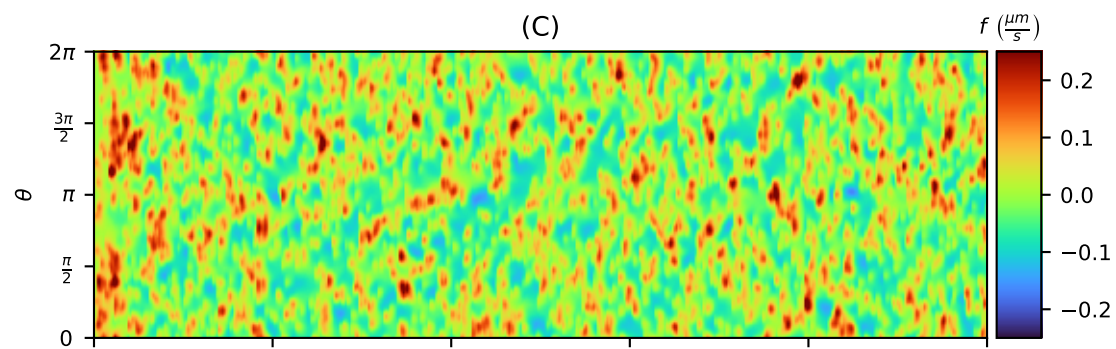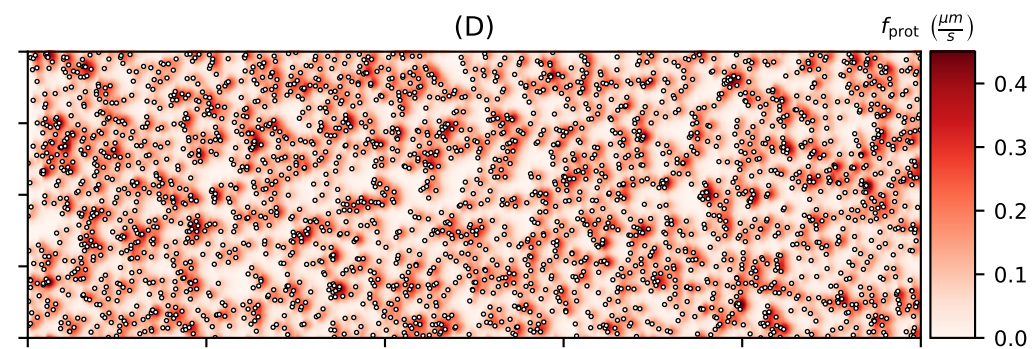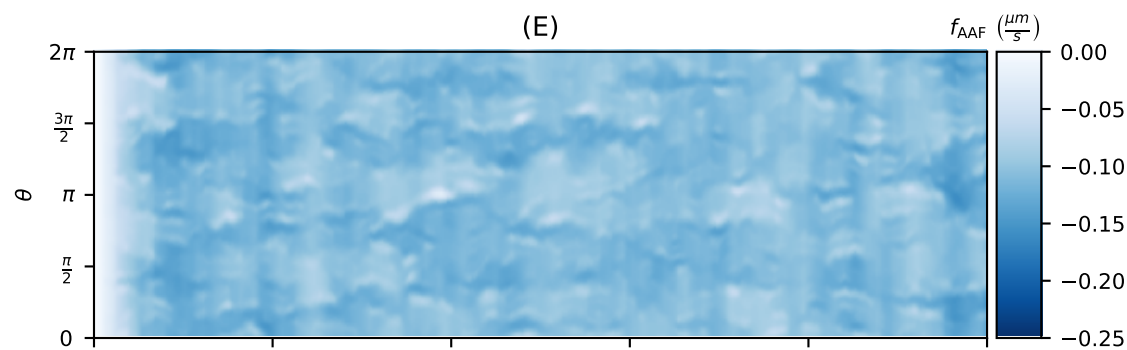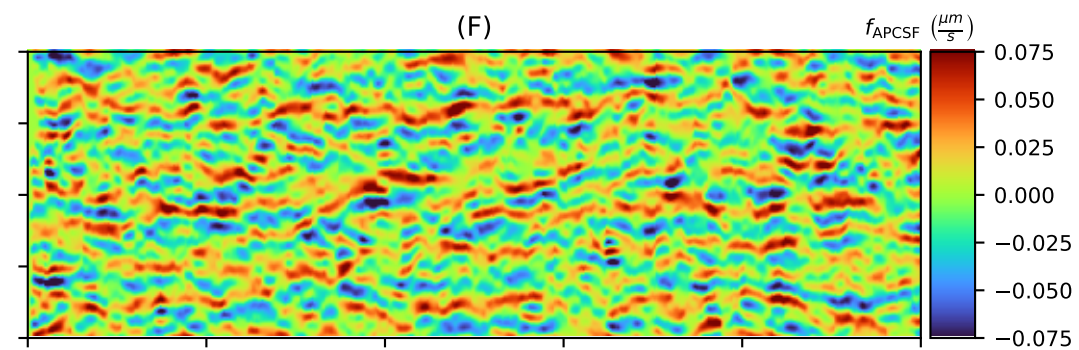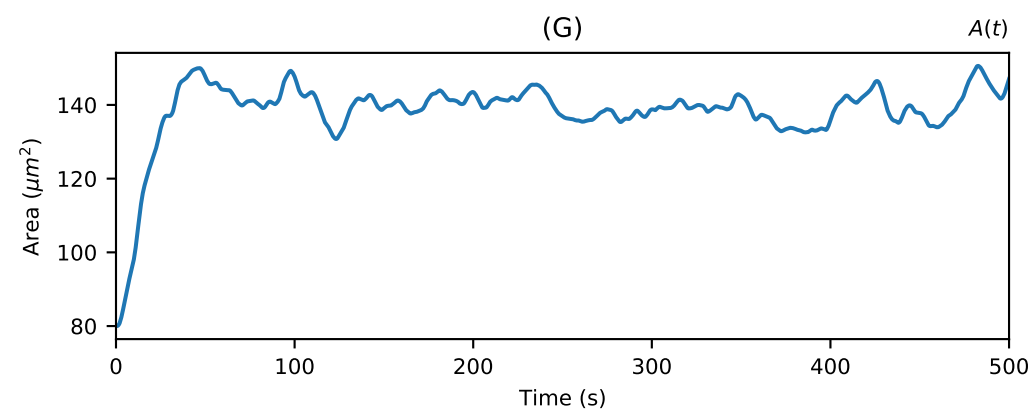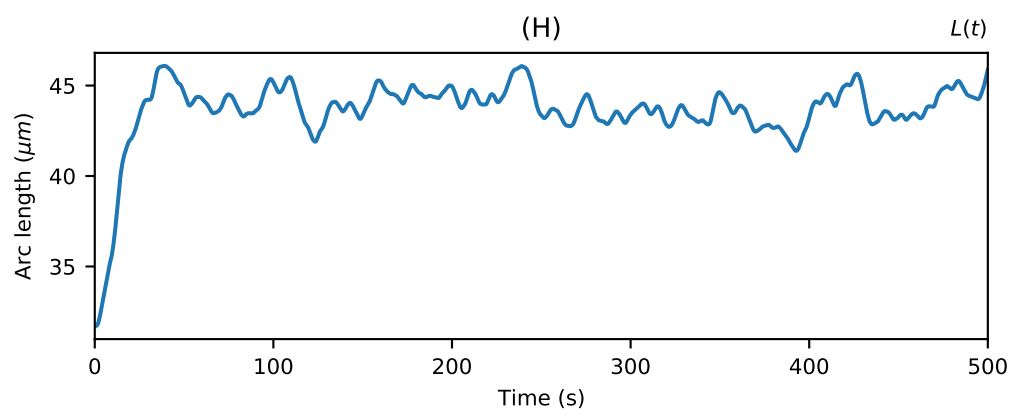

Supplement: S4 Fig — (PDF) [file pone.0297511.s005.pdf]

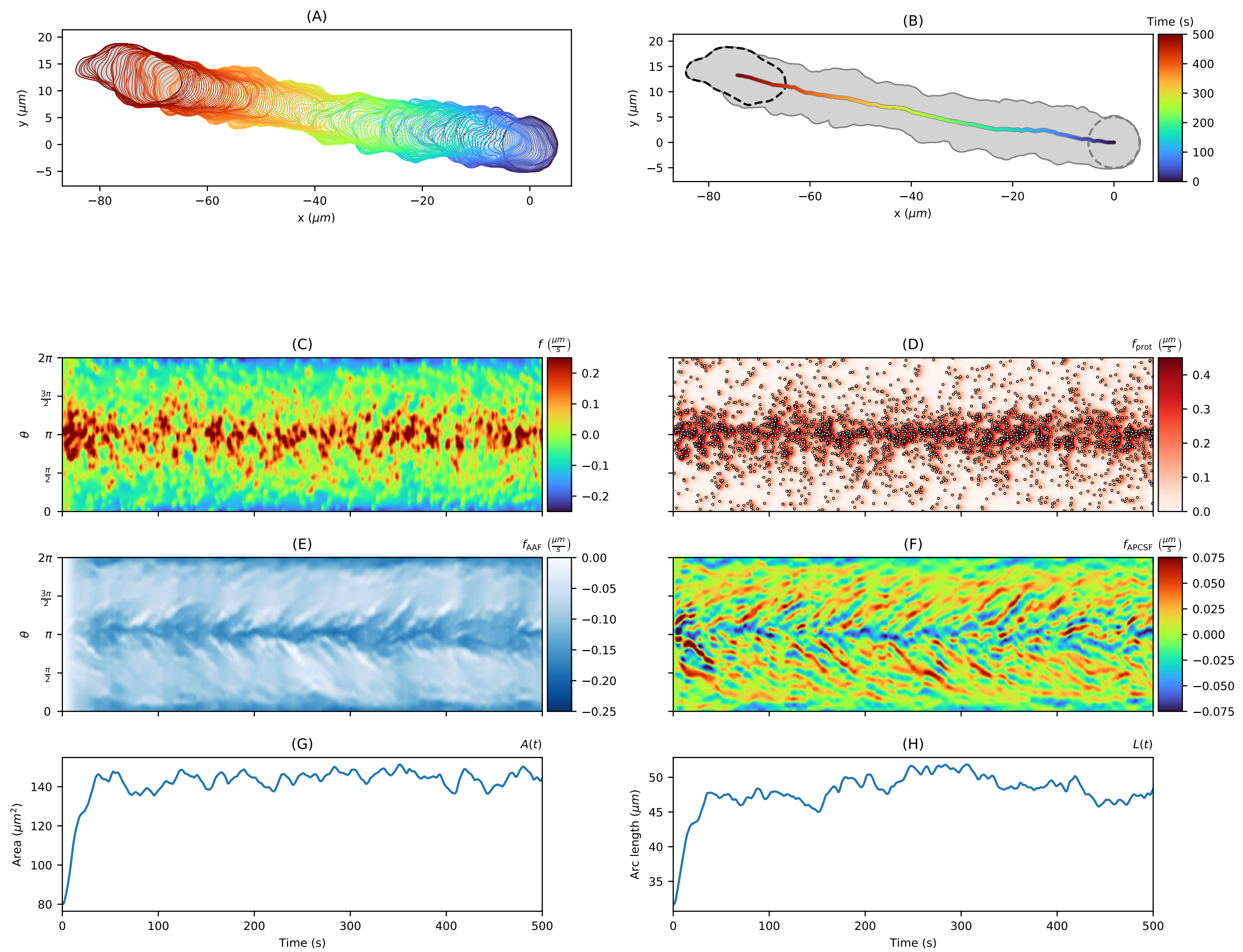

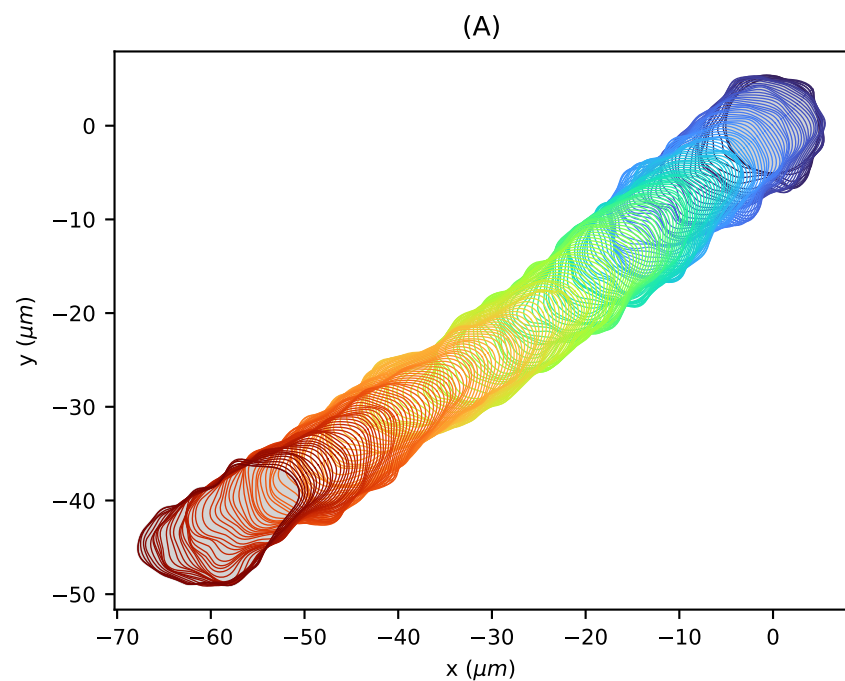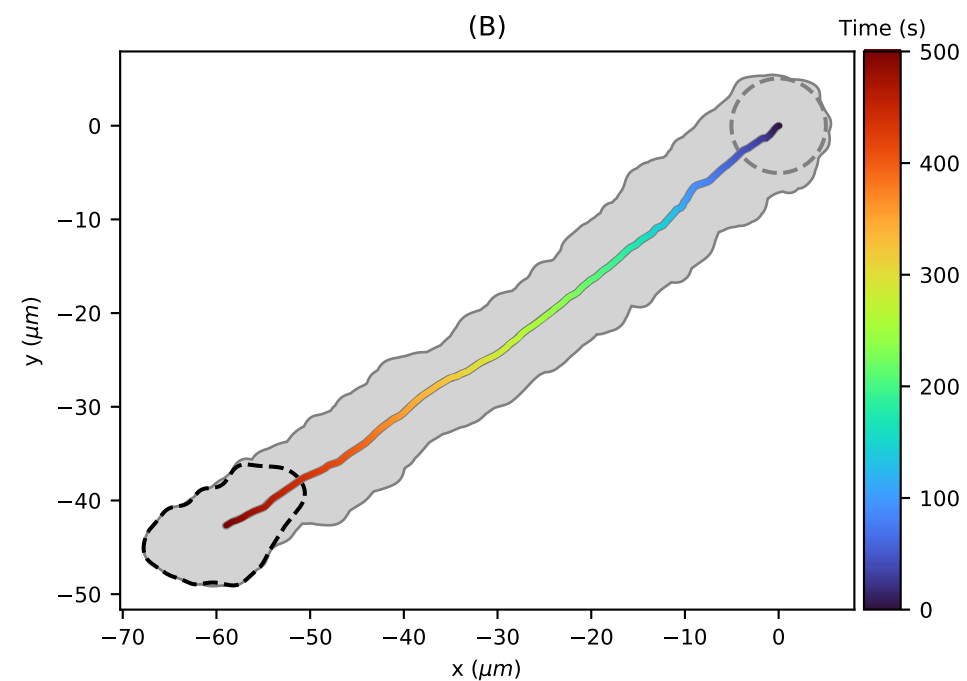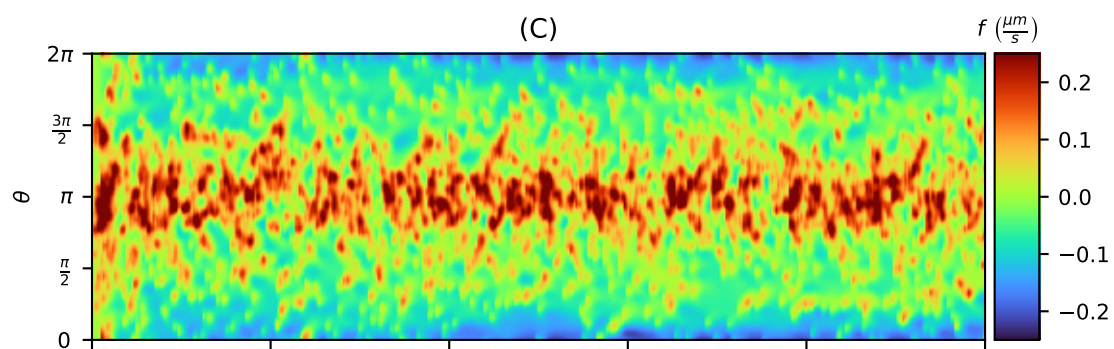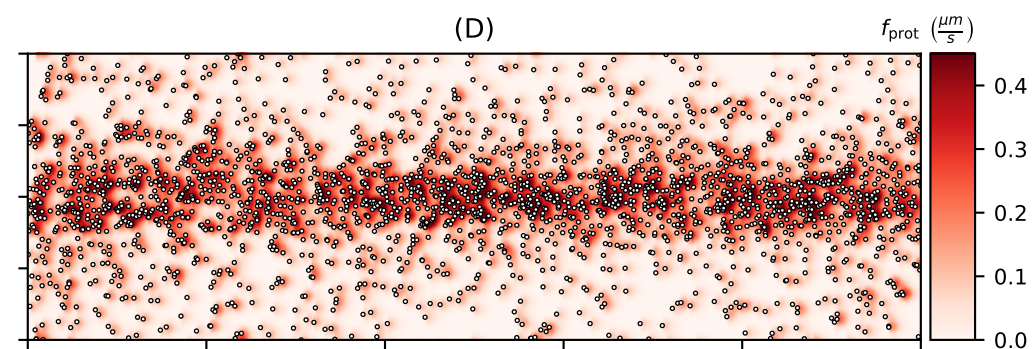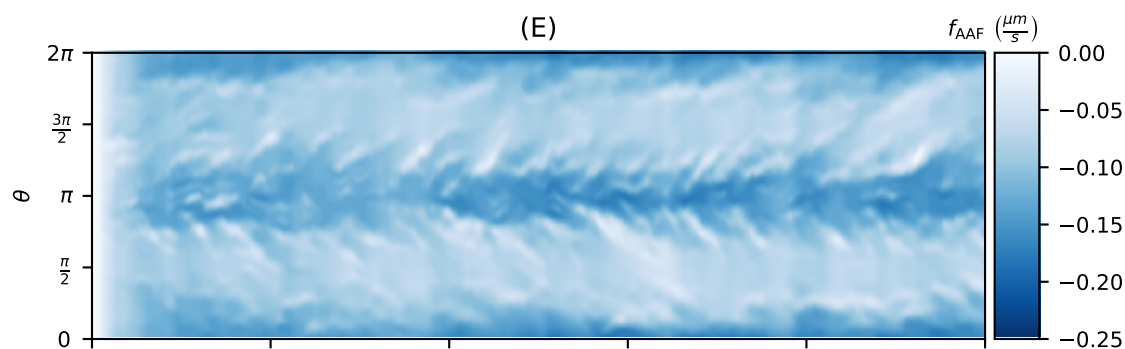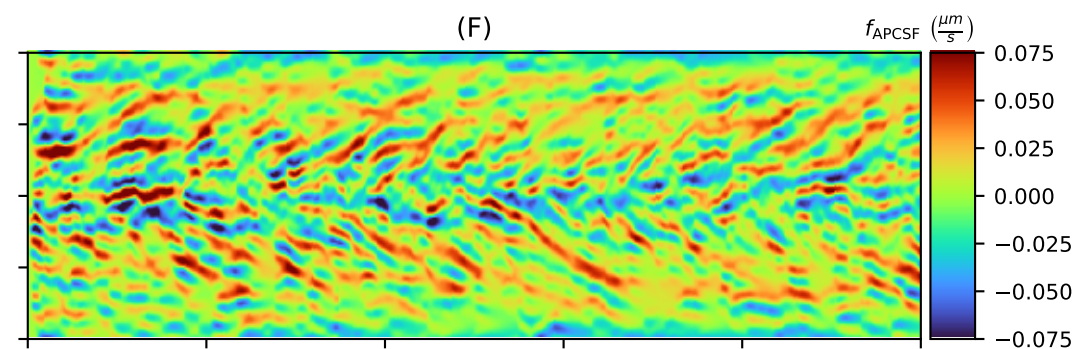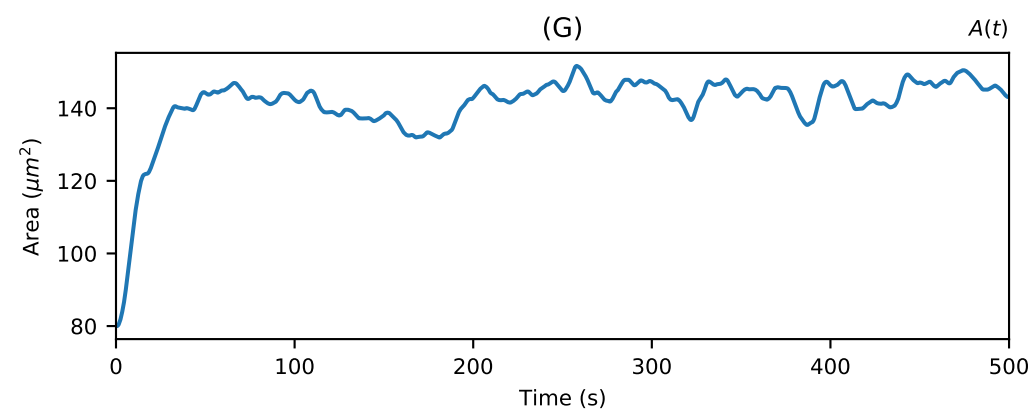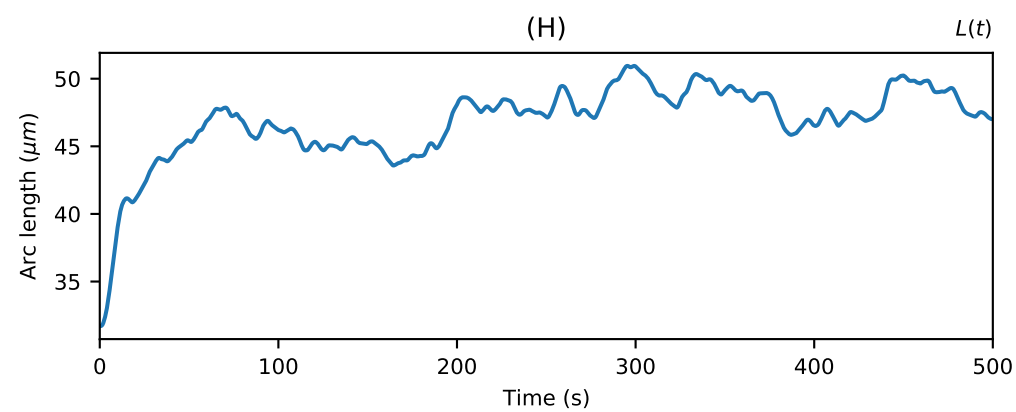

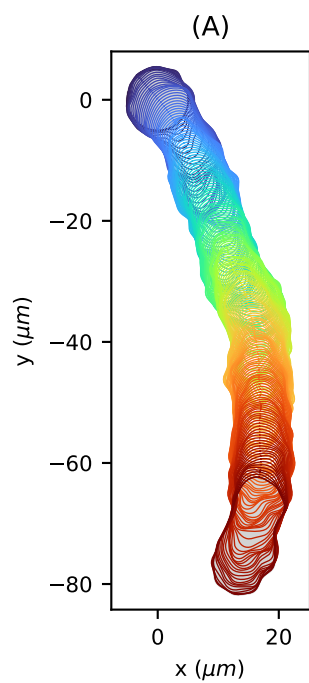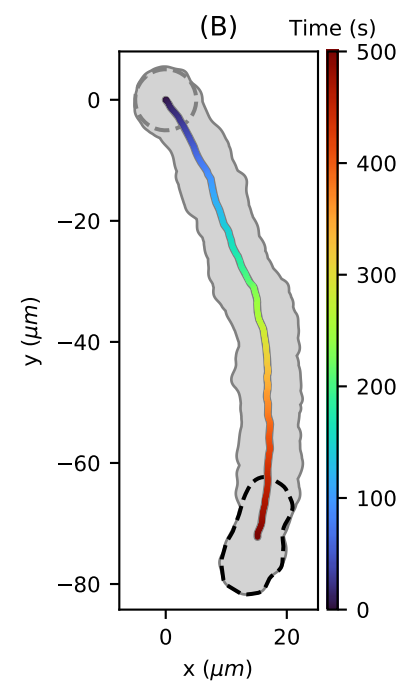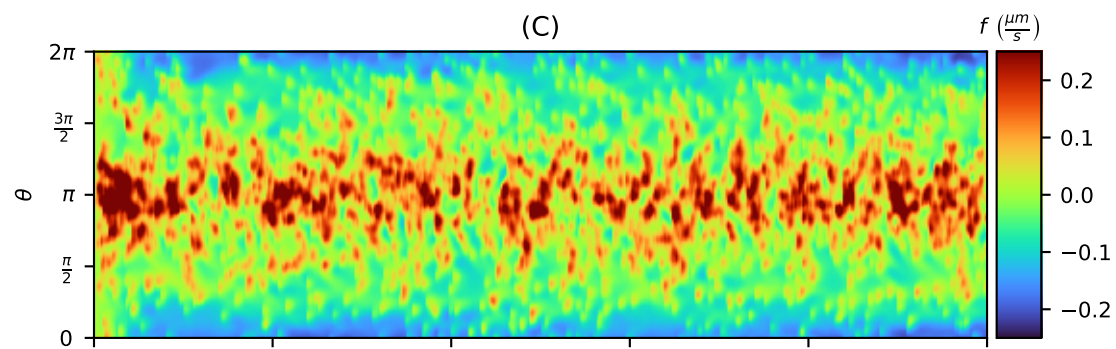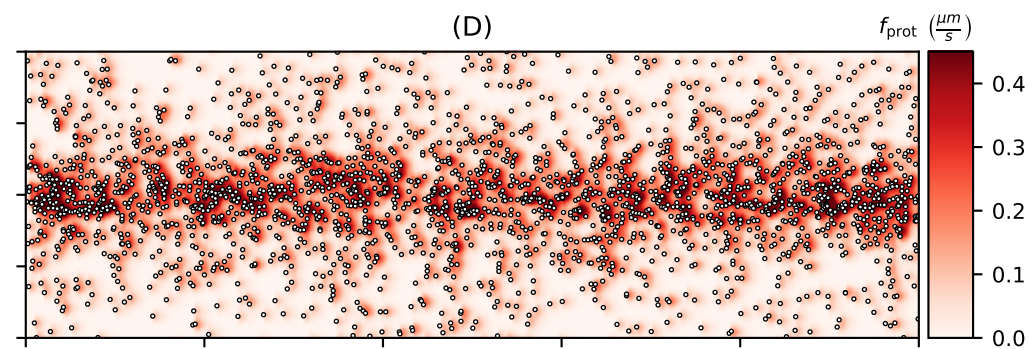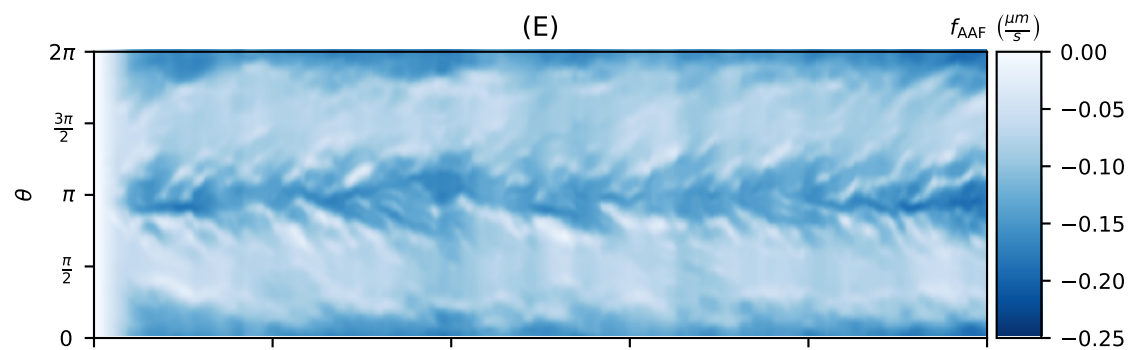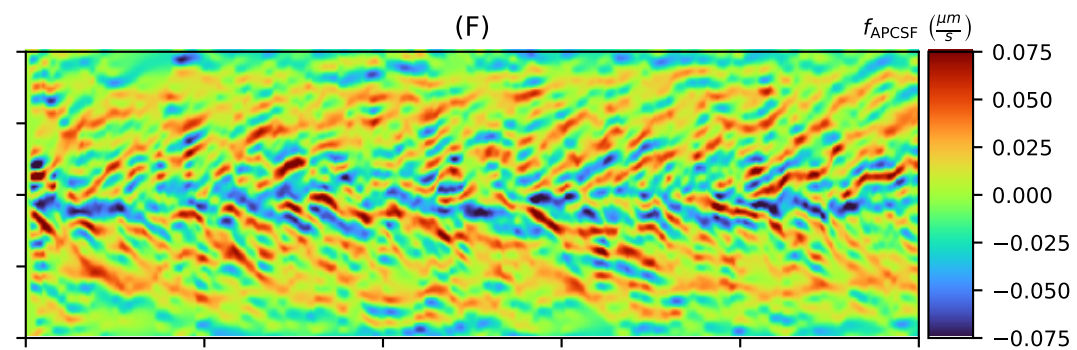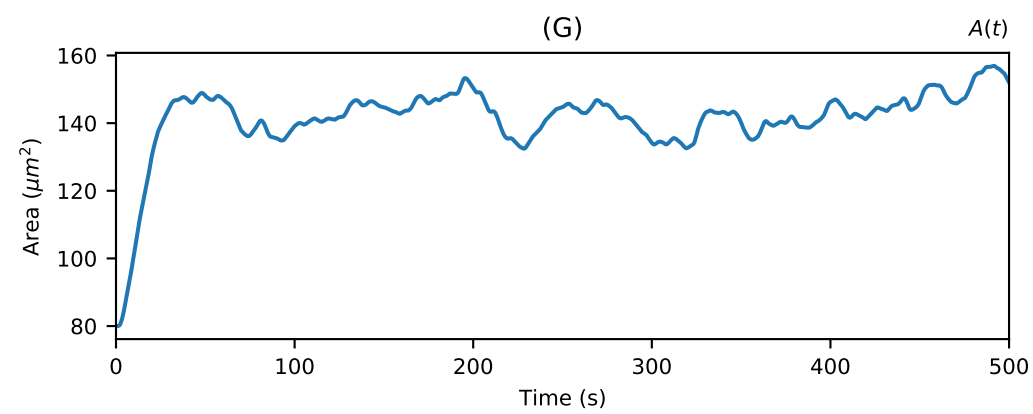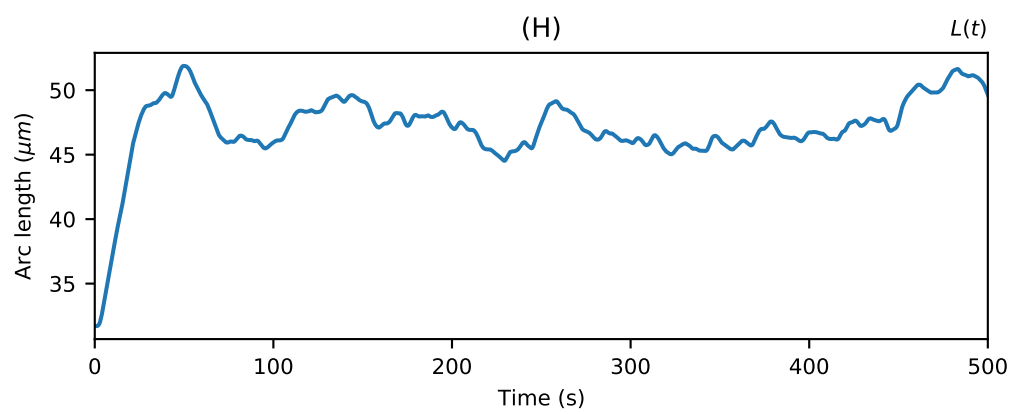

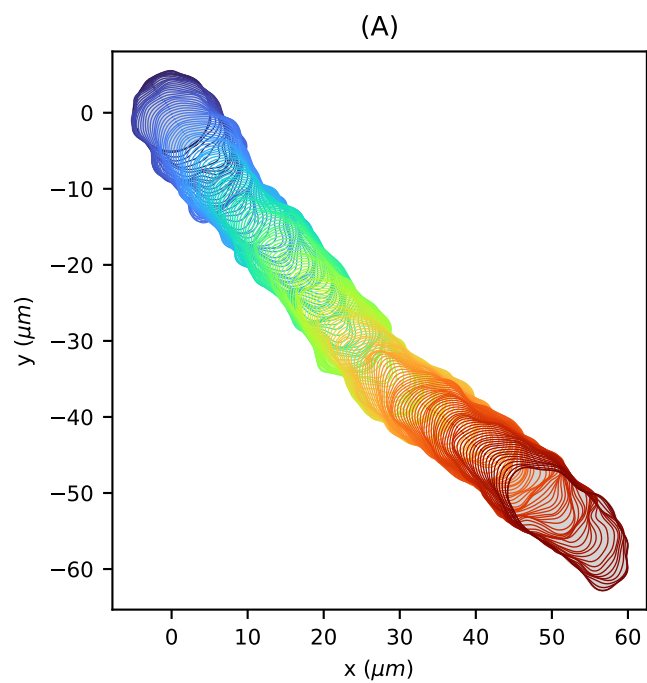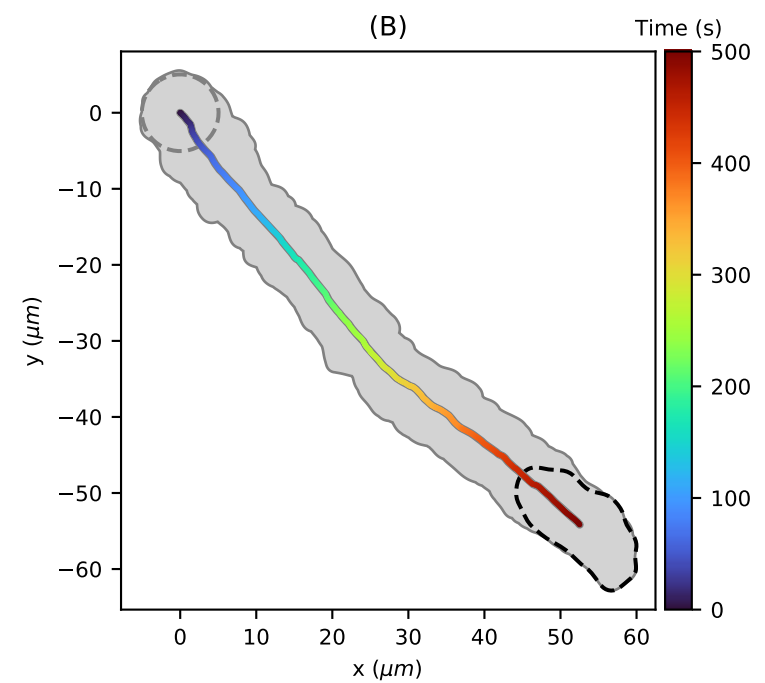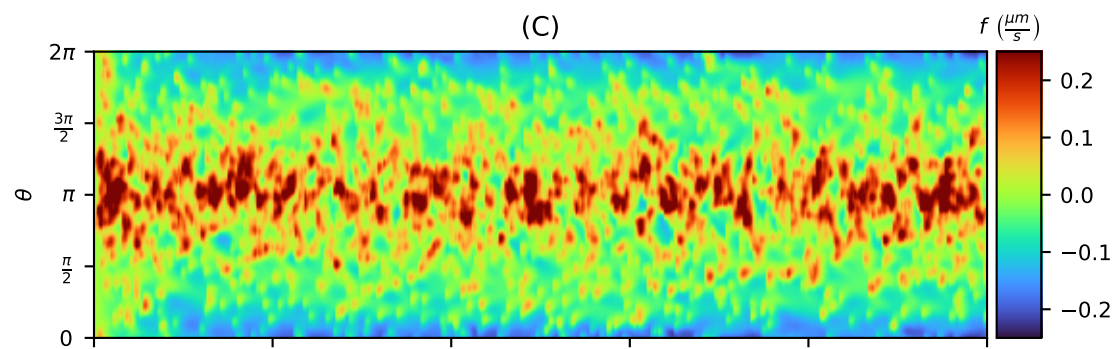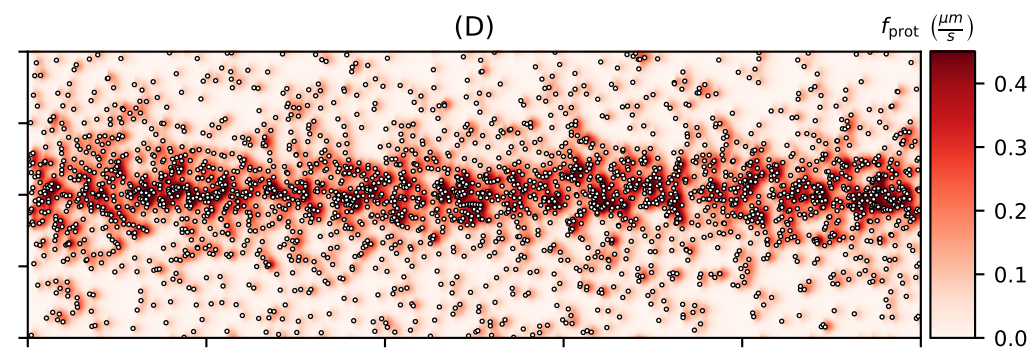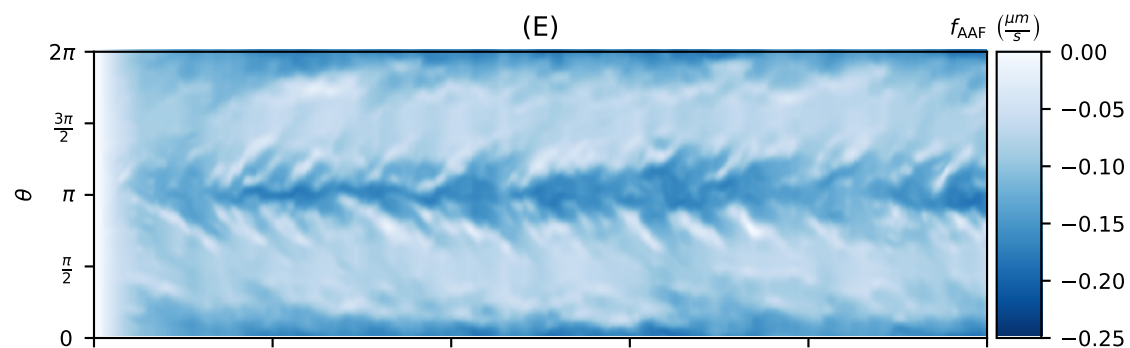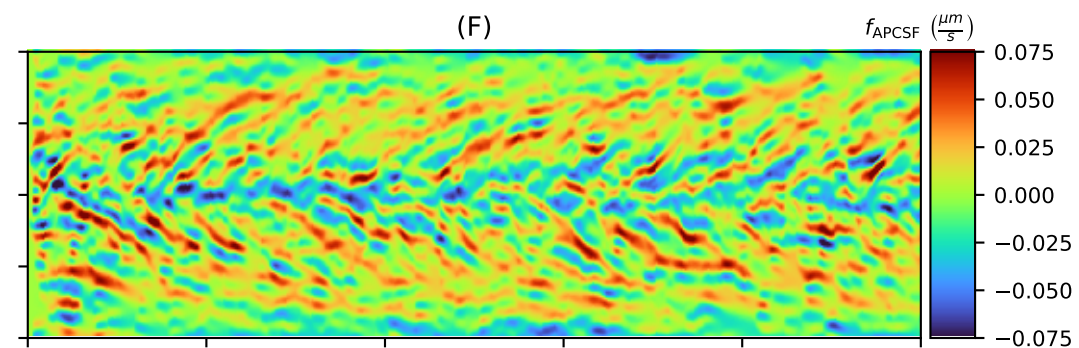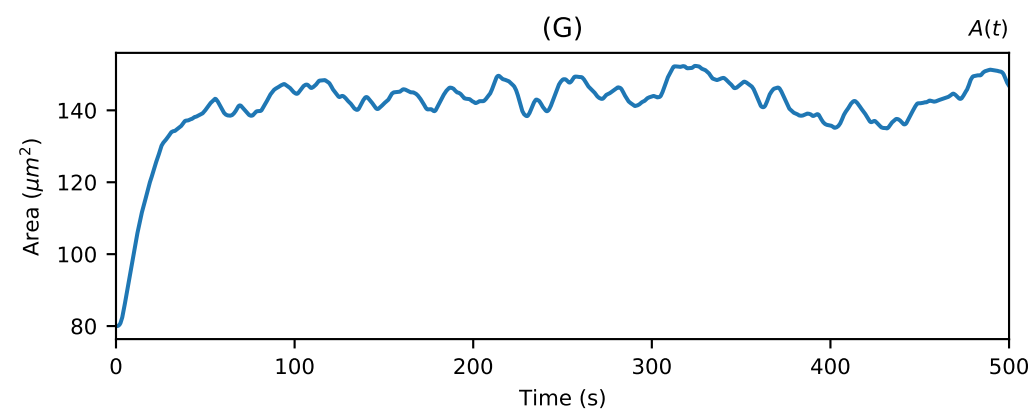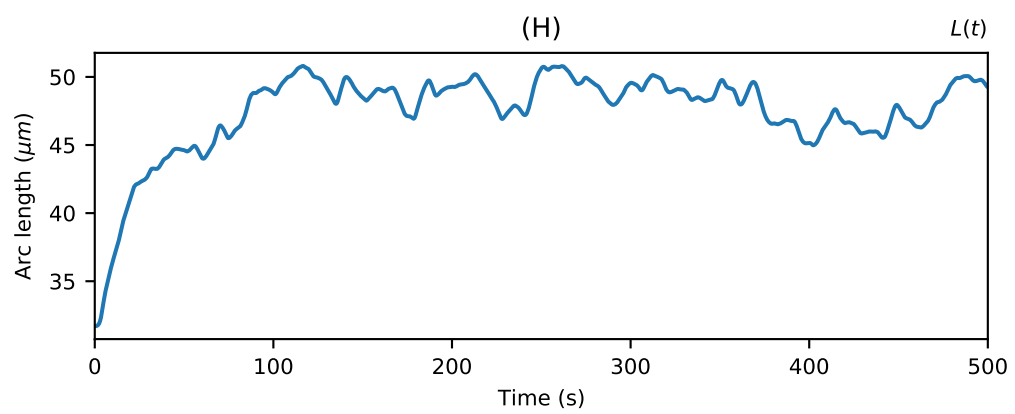

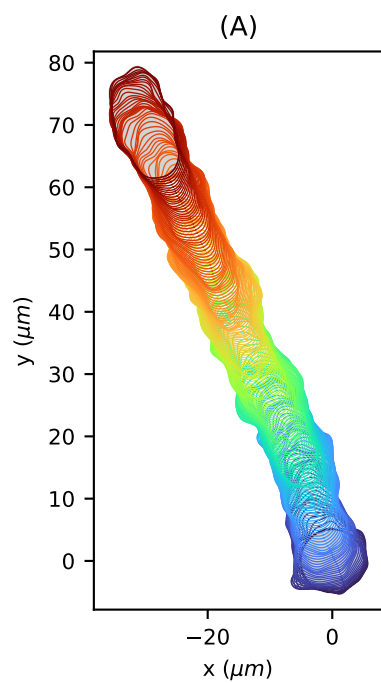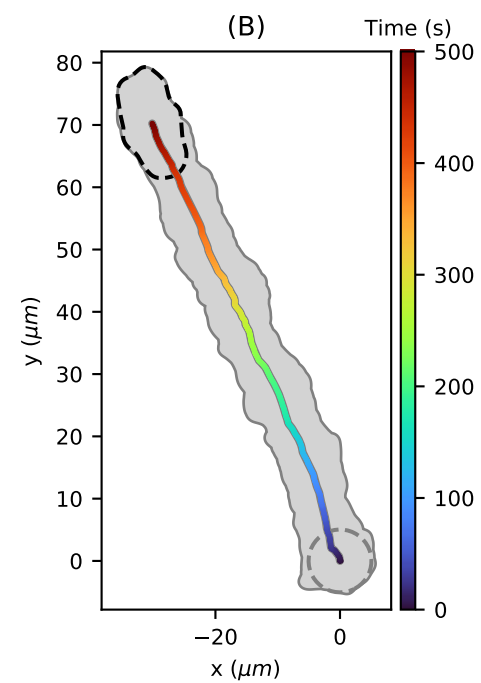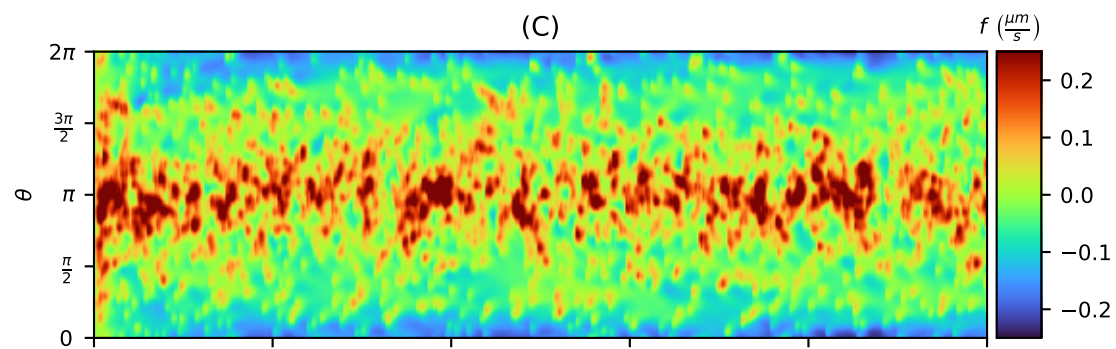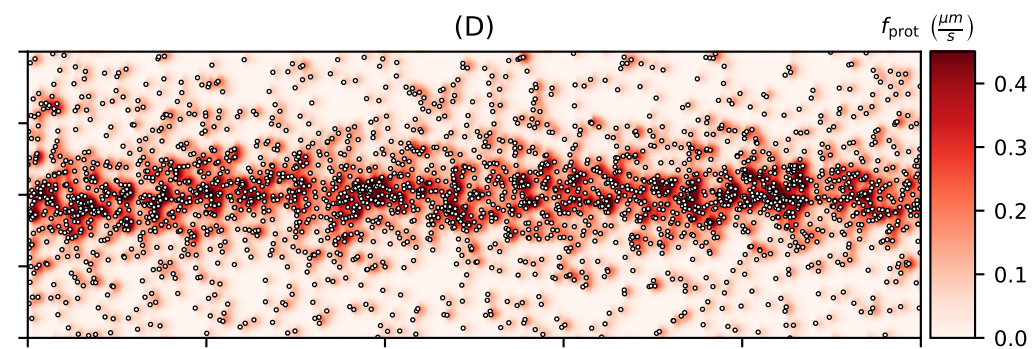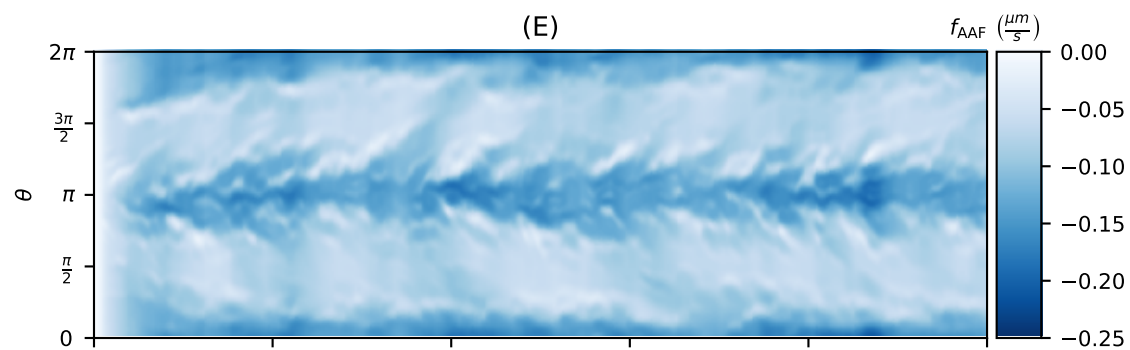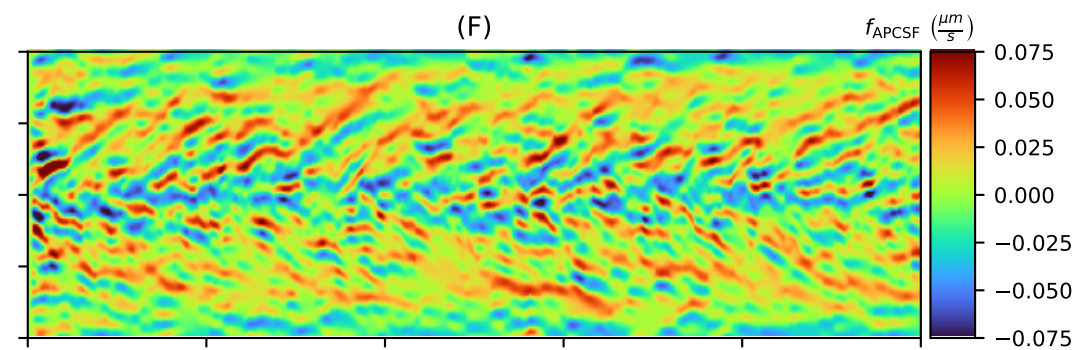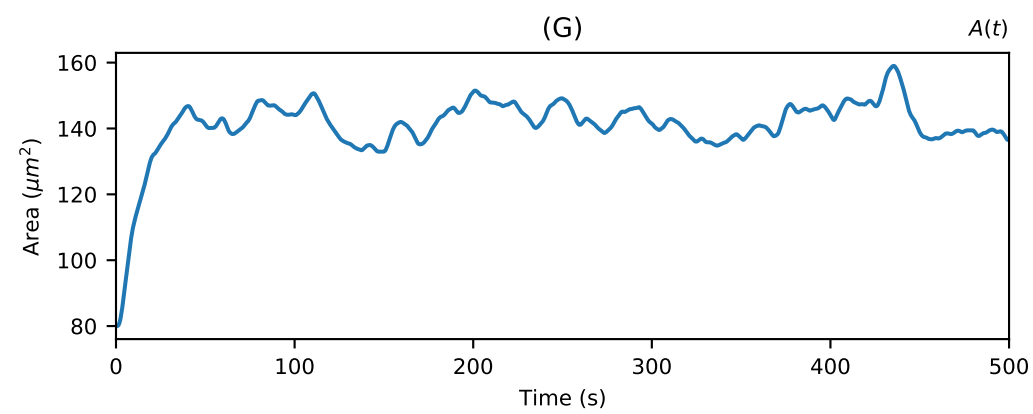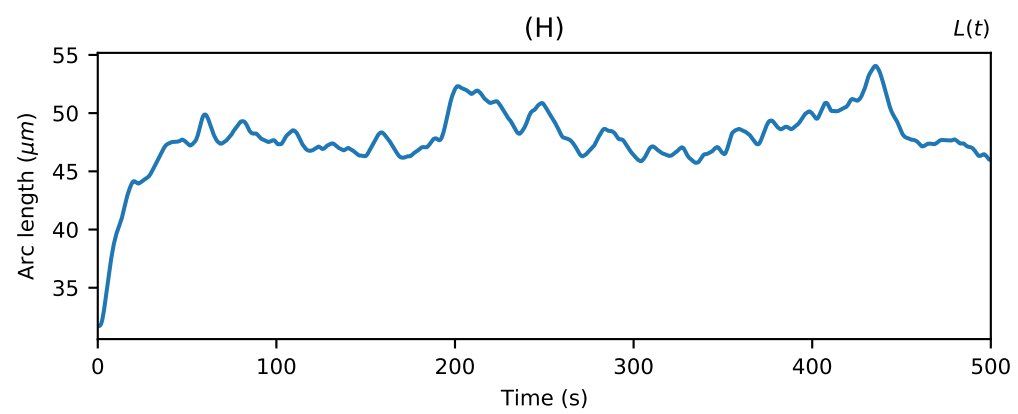

Supplement: S5 Fig — (PDF) [file pone.0297511.s006.pdf]
