# Supplementary material for: Three-component contour dynamics model to simulate and analyze amoeboid cell motility in two dimensions
Source: PLoS One. 2024 Jan 26;19(1):e0297511. doi: 10.1371/journal.pone.0297511 (PMC10817190; doi:10.1371/journal.pone.0297511)
Supplement: S6 Fig — (PDF) [file pone.0297511.s007.pdf]

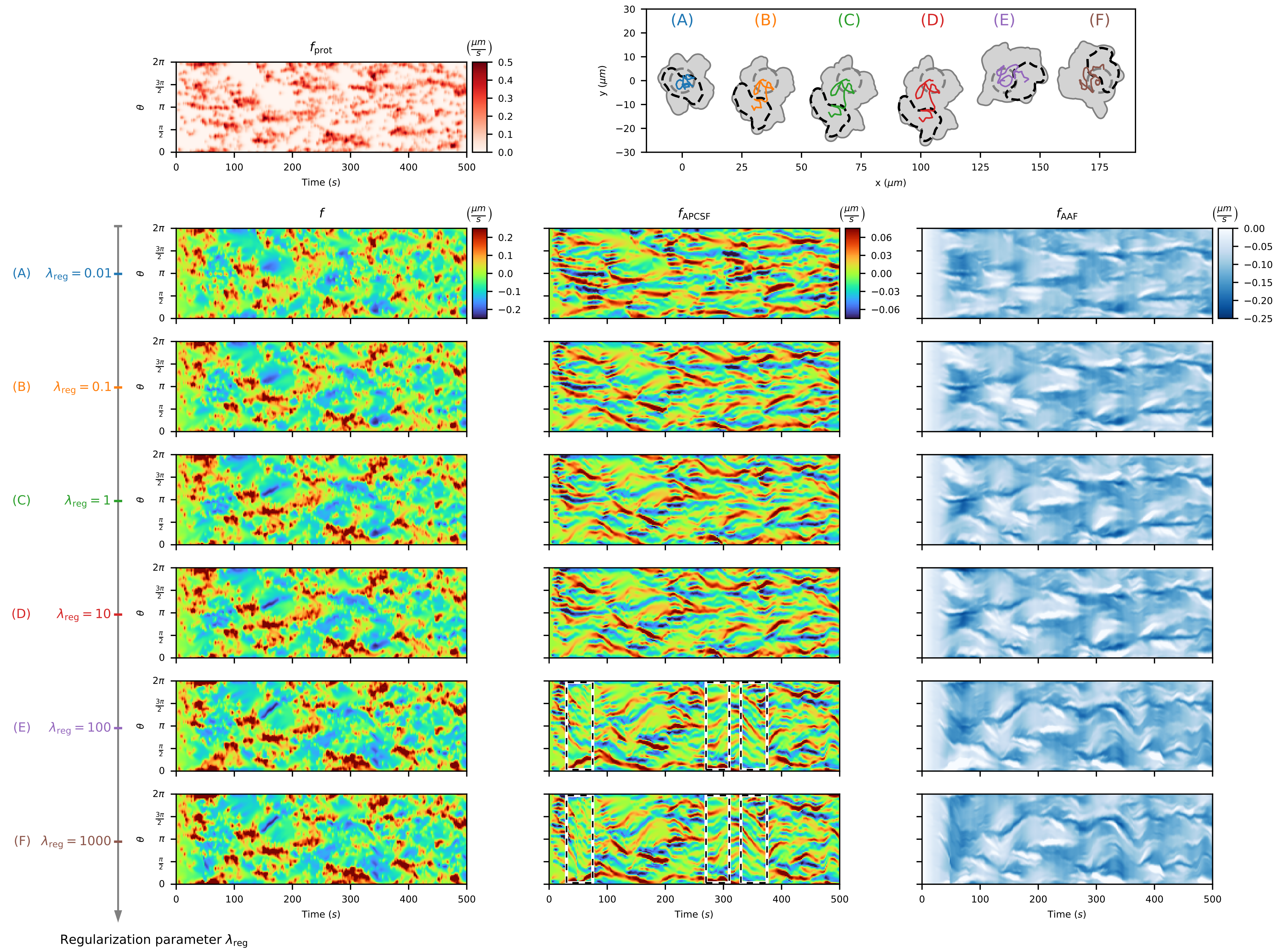

Fig S6. Parameter study with varying regularization parameter λ_{reg} during non-polarized cell motility. For each cell track the same protrusion component is underlying (top left). The center of mass trajectory (colored lines) as well as the covered area of each cell track (gray area) are displayed (top right). Based on different regularization schemes, different kymographs are computed: Local motion (left column), the APCSF component (middle column), and the AAF component (right column). Regions of interest are displayed as black and white dashed boxes indicating rotating elements of the cell contour (last two rows).
