# Supplementary material for: Three-component contour dynamics model to simulate and analyze amoeboid cell motility in two dimensions
Source: PLoS One. 2024 Jan 26;19(1):e0297511. doi: 10.1371/journal.pone.0297511 (PMC10817190; doi:10.1371/journal.pone.0297511)
Supplement: S8 Fig — (PDF) [file pone.0297511.s009.pdf]

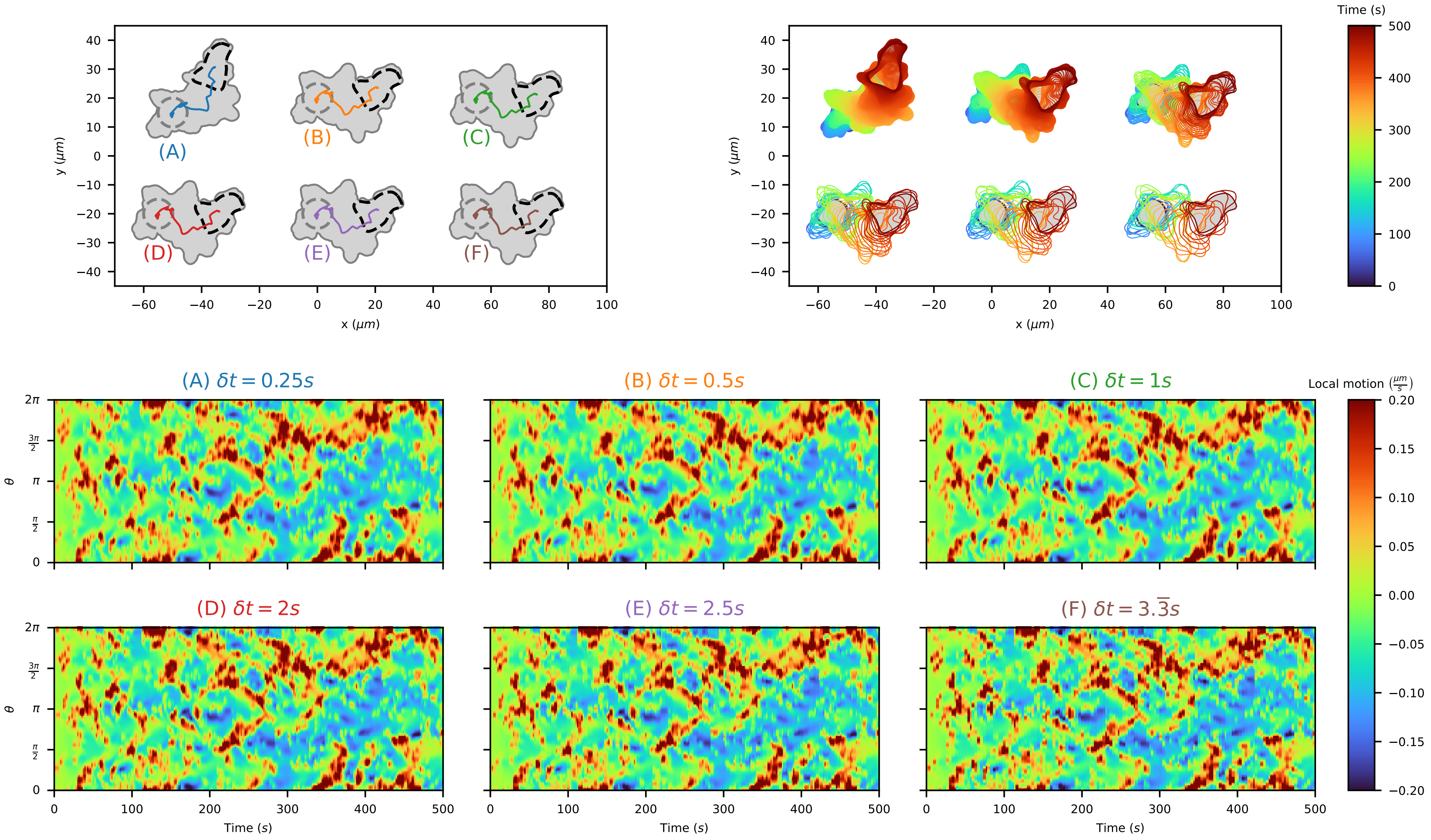

Fig S8. Comparison of local motion kymographs of simulated non-polarized cell tracks for different temporal resolutions: $\delta t \in \{0.25, 0.5, 1, 2, 2.5, 3.\overline{3}\}$. In the top left corner, the center of mass trajectory (colored lines) and the trace of each cell track (gray area) are displayed. In the top right corner, the contour dynamics (colored lines) and the trace of the cell track (gray area) are shown. Below, kymographs of the local motion based on our model are listed for each cell track.
