# Supplementary material for: Three-component contour dynamics model to simulate and analyze amoeboid cell motility in two dimensions
Source: PLoS One. 2024 Jan 26;19(1):e0297511. doi: 10.1371/journal.pone.0297511 (PMC10817190; doi:10.1371/journal.pone.0297511)
Supplement: S10 Fig — (PDF) [file pone.0297511.s011.pdf]

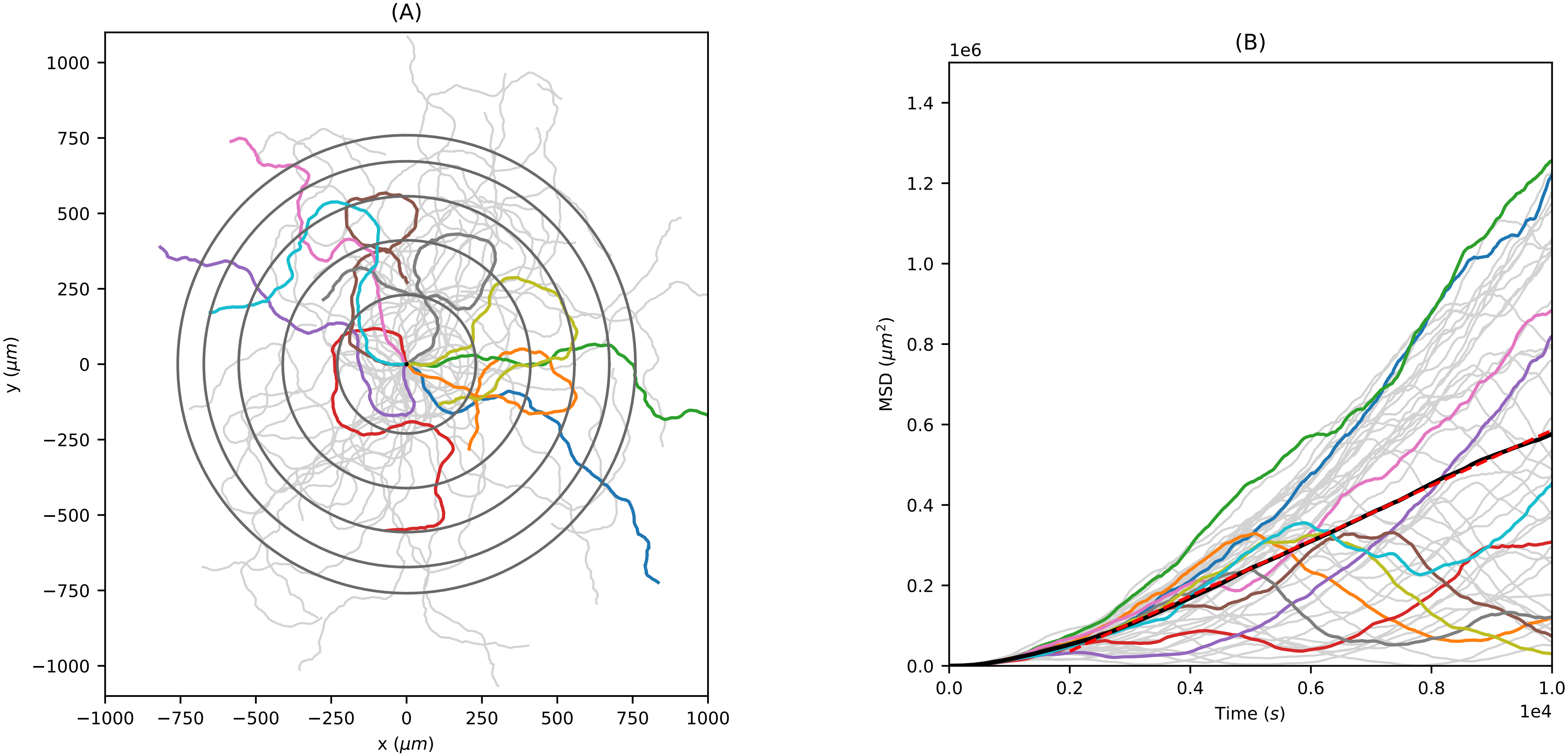

Fig S10. Long-term diffusion analysis of polarized artificial cell tracks based on a Hawkes process. **(A)** Center of mass trajectories of 50 polarized cell tracks over a longer time period $T = 10000s$ (colored and gray lines). The root mean squared displacement (RMSD) is shown for every 2000s (gray circles). **(B)** Corresponding mean squared displacement (MSD, bold black line) with linear fit (dashed red line).
