# Supplementary material for: Three-component contour dynamics model to simulate and analyze amoeboid cell motility in two dimensions
Source: PLoS One. 2024 Jan 26;19(1):e0297511. doi: 10.1371/journal.pone.0297511 (PMC10817190; doi:10.1371/journal.pone.0297511)
Supplement: S11 Fig — (PDF) [file pone.0297511.s012.pdf]

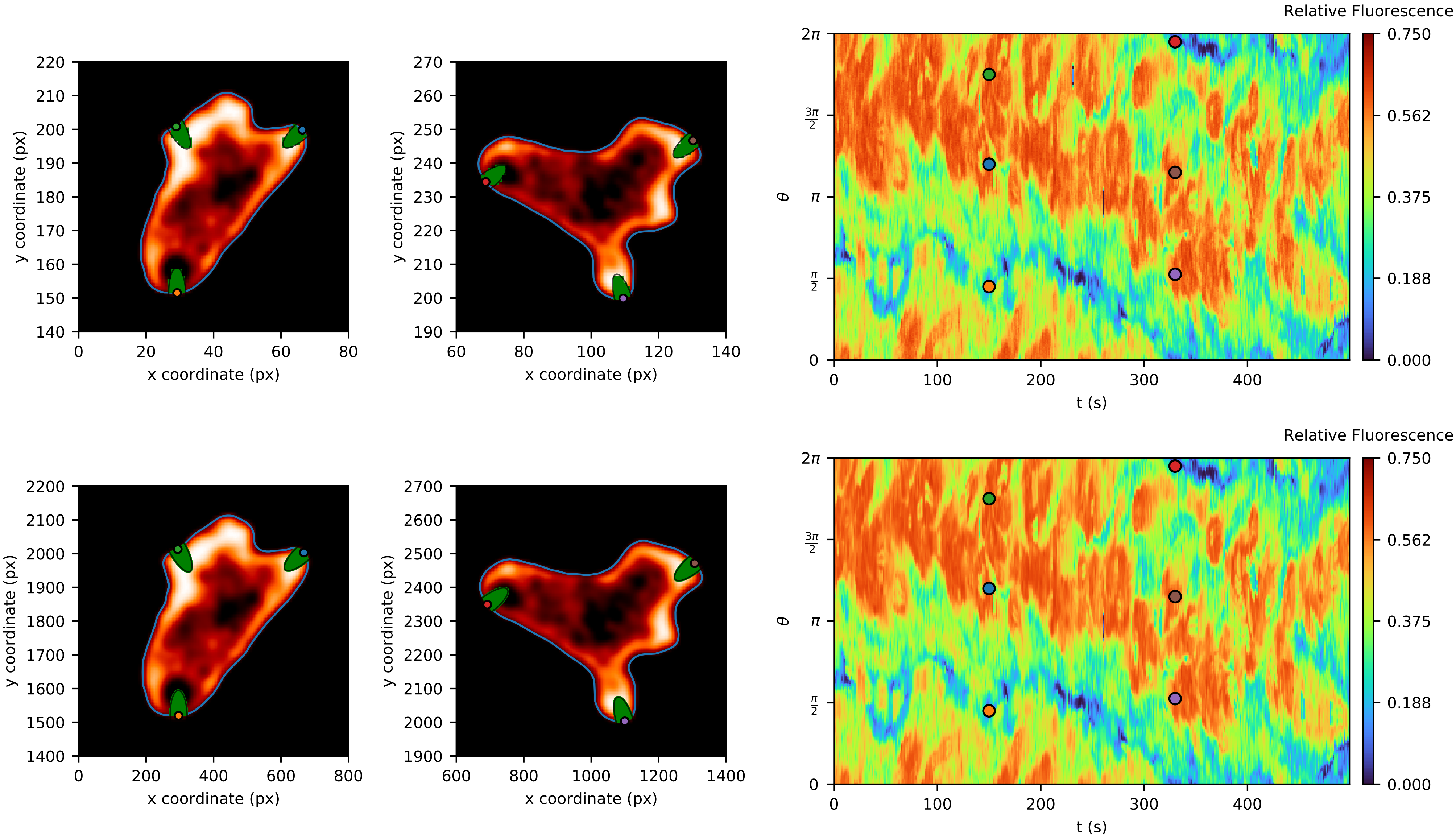

Fig S11. Computation of relative fluorescence intensity for experimental microscopy data and tenfold upsampled data via ellipses along the cell contour. **(Top row)** Two exemplary frames of the original microscopy data from which the segmented contour (blue) is derived. The relative fluorescence intensity averaged over ellipses along the cell contour (green) are then translated into a kymograph (right column). The position of six exemplary virtual markers on the cell contour and in the kymograph are displayed as colored dots. **(Bottom row)** Tenfold upsampled image data with corresponding kymograph which shows almost no deviations from the original kymograph.
