# Supplementary material for: Three-component contour dynamics model to simulate and analyze amoeboid cell motility in two dimensions
Source: PLoS One. 2024 Jan 26;19(1):e0297511. doi: 10.1371/journal.pone.0297511 (PMC10817190; doi:10.1371/journal.pone.0297511)
Supplement: S12 Fig — The model weights were estimated by minimizing sums of squared residuals: S (left column) and S+ (right column), see S1 Text for more details. (PDF) [file pone.0297511.s013.pdf]

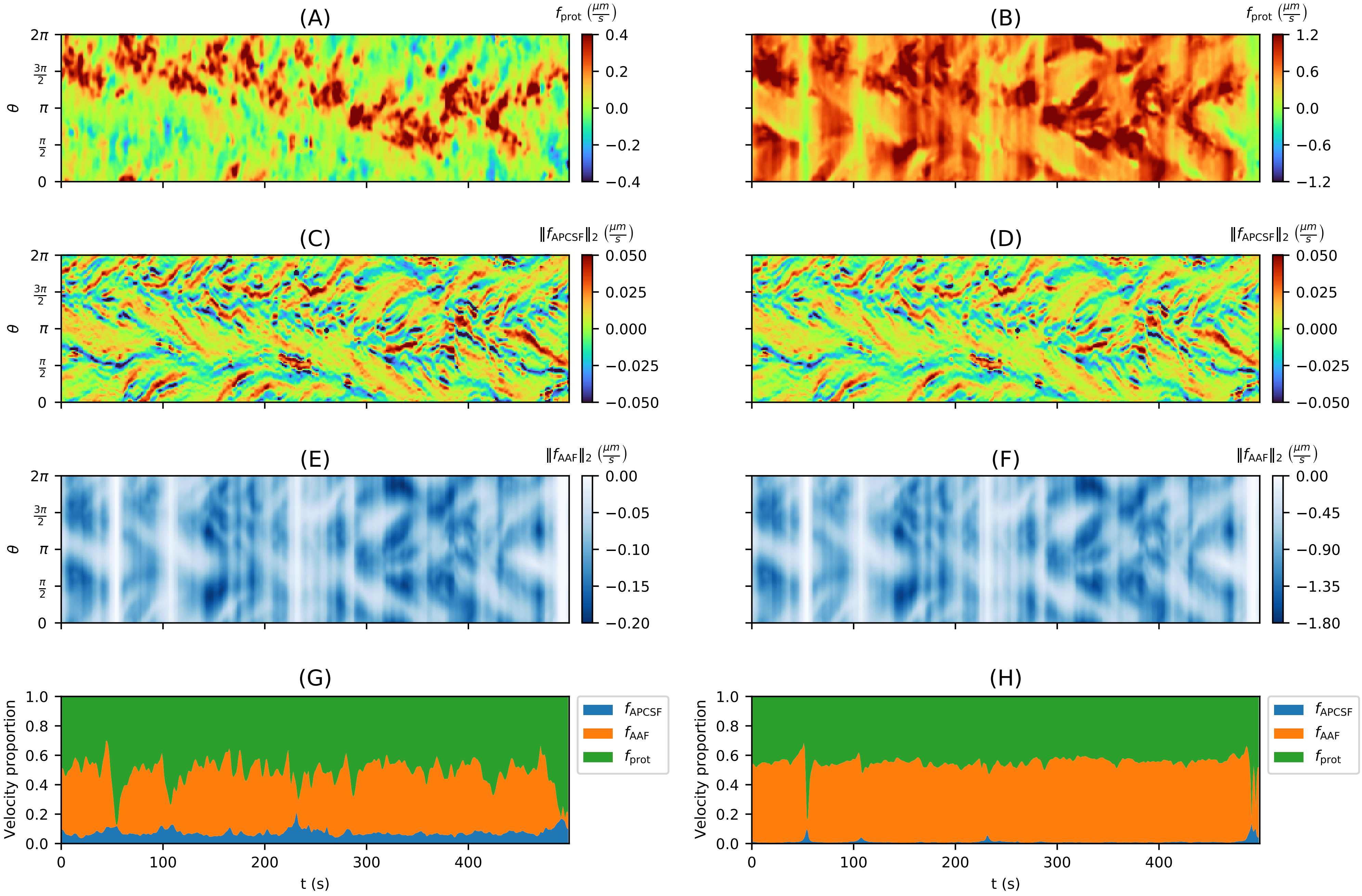

Fig S12. Model components extracted from experimental cell track of Fig 7 for two pairs of model weights (w_{prot}, w_{APCSF}, w_{AAF}): (6.634, 0.057, 3.532) and (17.190, 0.047, 31.076). The model weights were estimated by minimizing sums of squared residuals: S (left column) and S^+ (right column), see S1 Text for more details. While the first approach enforces small corrections of f_{prot}, positive values of f_{prot} are favored in the second approach. The following kymographs are displayed: Protrusion component **(A, B)**, APCSF component **(C, D)**, and AAF component **(E, F)**, as well as their proportion to the overall velocity of the contour dynamics **(G, H)**.
