# Supplementary material for: Three-component contour dynamics model to simulate and analyze amoeboid cell motility in two dimensions
Source: PLoS One. 2024 Jan 26;19(1):e0297511. doi: 10.1371/journal.pone.0297511 (PMC10817190; doi:10.1371/journal.pone.0297511)
Supplement: S13 Fig — (PDF) [file pone.0297511.s014.pdf]

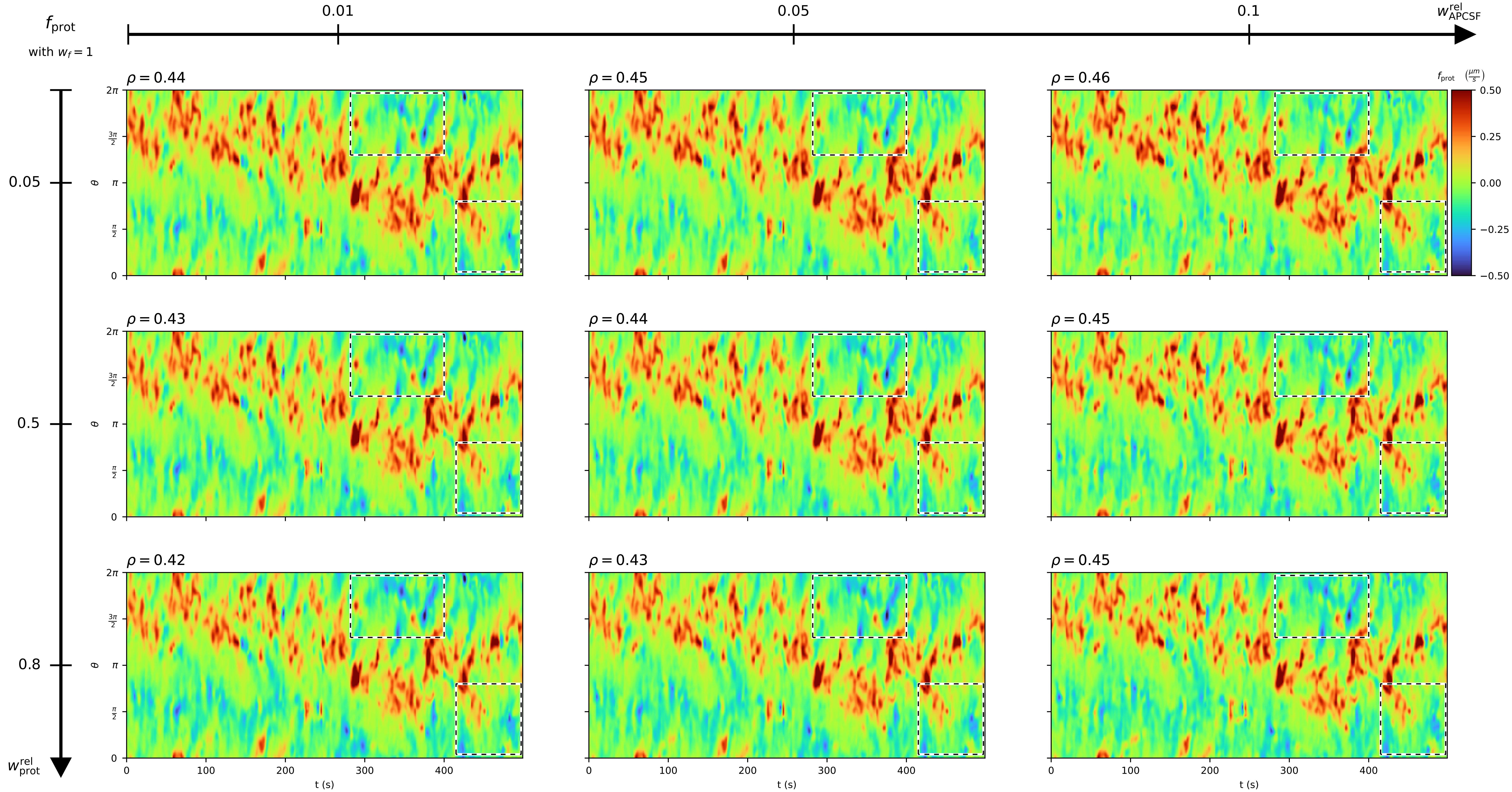

Fig S13. Protrusion component f_{prot} extracted from the experimental cell track of Fig 7 for varying relative weights $w_{\text{prot}}^{\text{rel}} \in \{0.05, 0.5, 0.8\}$ (vertical axis) and $w_{\text{APCSF}}^{\text{rel}} \in \{0.01, 0.05, 0.1\}$ (horizontal axis) as well as varying overall velocity parameter $w_f \in \{1, 5, 10, 20\}$ (page axis). The Pearson correlation coefficient ρ between the protrusion component and the fluorescence intensity kymograph from Fig 7D is displayed above each kymograph. Regions of interest are displayed as black and white dashed boxes.

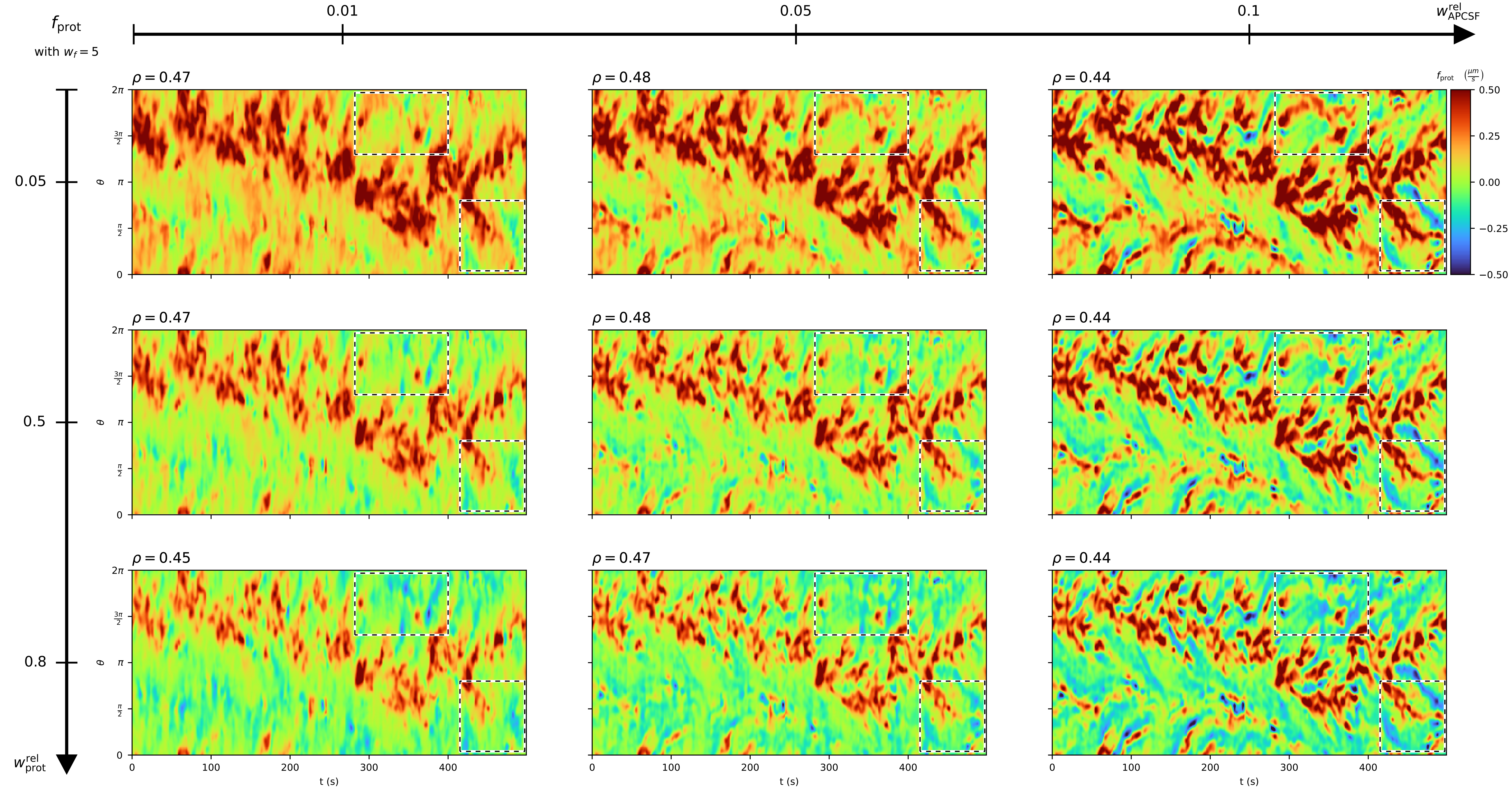

Fig S13. Protrusion component f_{prot} extracted from the experimental cell track of Fig 7 for varying relative weights $w^{\text{rel}}_{\text{prot}} \in \{0.05, 0.5, 0.8\}$ (vertical axis) and $w^{\text{rel}}_{\text{APCSF}} \in \{0.01, 0.05, 0.1\}$ (horizontal axis) as well as varying overall velocity parameter $w_f \in \{1, 5, 10, 20\}$ (page axis). The Pearson correlation coefficient ρ between the protrusion component and the fluorescence intensity kymograph from Fig 7D is displayed above each kymograph. Regions of interest are displayed as black and white dashed boxes.

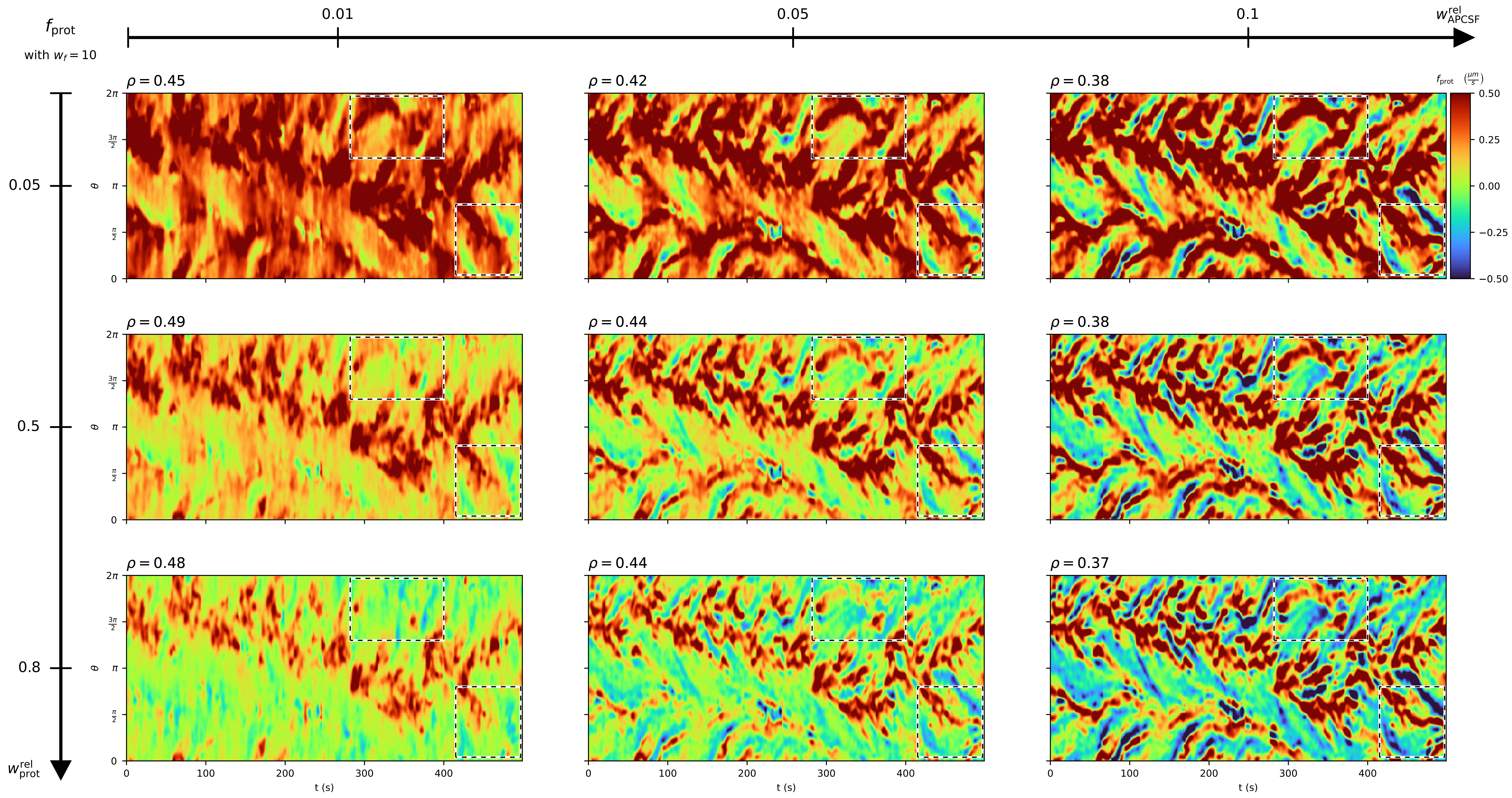

Fig S13. Protrusion component f_{prot} extracted from the experimental cell track of Fig 7 for varying relative weights $w^{\text{rel}}_{\text{prot}} \in \{0.05, 0.5, 0.8\}$ (vertical axis) and $w^{\text{rel}}_{\text{APCSF}} \in \{0.01, 0.05, 0.1\}$ (horizontal axis) as well as varying overall velocity parameter $w_f \in \{1, 5, 10, 20\}$ (page axis). The Pearson correlation coefficient ρ between the protrusion component and the fluorescence intensity kymograph from Fig 7D is displayed above each kymograph. Regions of interest are displayed as black and white dashed boxes.

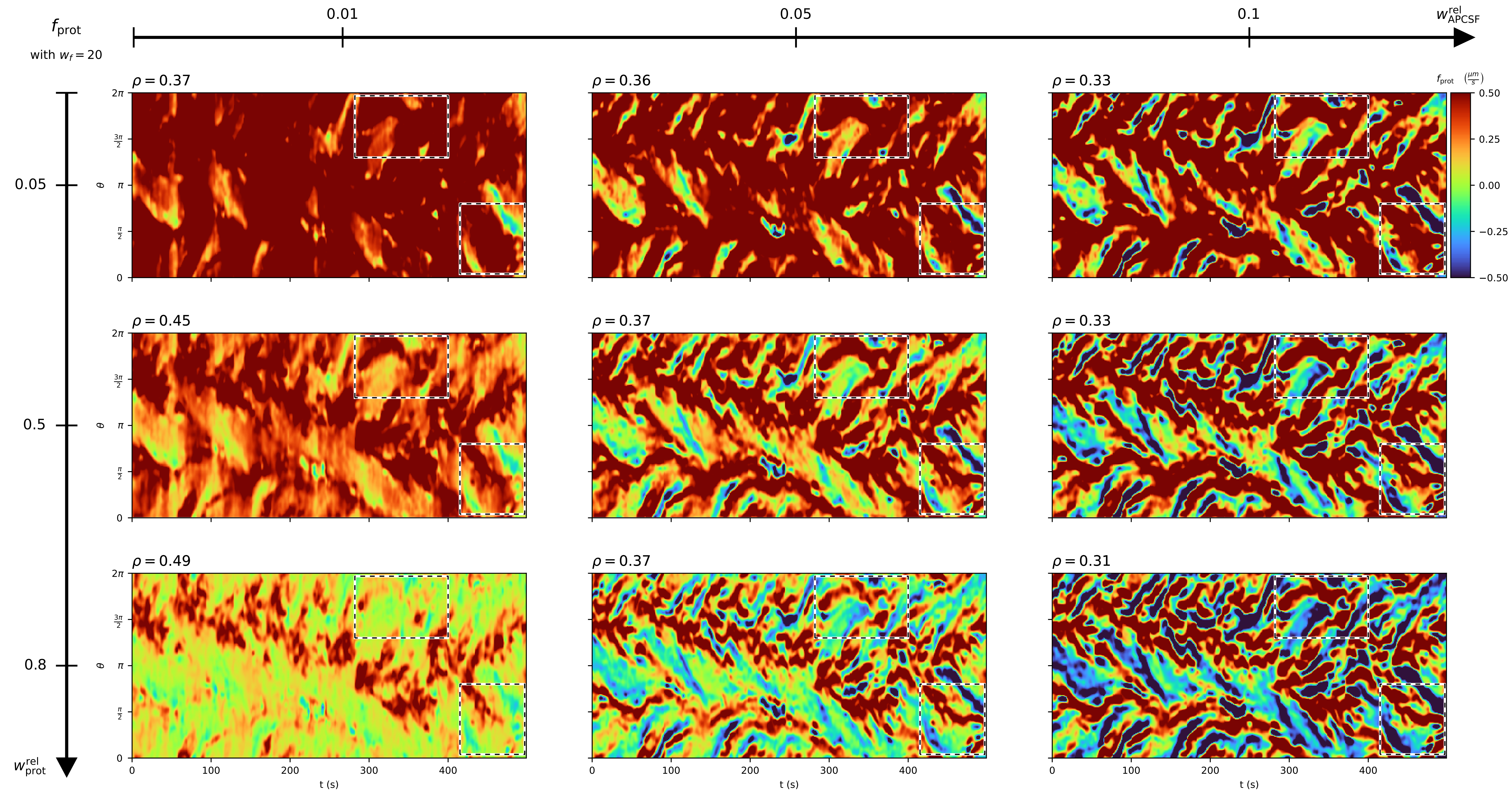

Fig S13. Protrusion component f_{prot} extracted from the experimental cell track of Fig 7 for varying relative weights $w^{rel}_{prot} \in \{0.05, 0.5, 0.8\}$ (vertical axis) and $w^{rel}_{APCSF} \in \{0.01, 0.05, 0.1\}$ (horizontal axis) as well as varying overall velocity parameter $w_f \in \{1, 5, 10, 20\}$ (page axis). The Pearson correlation coefficient ρ between the protrusion component and the fluorescence intensity kymograph from Fig 7D is displayed above each kymograph. Regions of interest are displayed as black and white dashed boxes.
