# Supplementary material for: Three-component contour dynamics model to simulate and analyze amoeboid cell motility in two dimensions
Source: PLoS One. 2024 Jan 26;19(1):e0297511. doi: 10.1371/journal.pone.0297511 (PMC10817190; doi:10.1371/journal.pone.0297511)
Supplement: Attachment — Submitted filename: response_to_reviewers.pdf [file pone.0297511.s020.pdf]

Response to the reviewers' comments

Preface

We highly appreciated the constructive comments and remarks by the reviewers. We have addressed all comments and remarks and revised the manuscript accordingly. In the point-to-point response below, answers to reviewer comments are highlighted in blue and changes to the manuscript in green.

Finally, we would like to thank the reviewers and associated editor for their time and consideration.

Contents

Reviewer #1 (Remarks to the Author): 2
Reviewer #2 (Remarks to the Author): 6

Reviewer #1 (Remarks to the Author):

The manuscript from Schindler et al. presents a model used to generate two dimensional cell morphologies similar to those observed in migrating amoeboid cells (e.g. Dictyostelium cells). The three components alluded to in the title refer to protrusive forces, an area regularization components, and a curvature-like preserving force. Over the last decade or so, a number of models have been put forward to generate trajectories such as those presented in this manuscript (such techniques were recently reviewed in DiNapoli et al., doi.org/10.1002/wsbm.1514 which should probably be cited here). The inclusion of these three components is fairly standard in these other modeling techniques, and it is pretty clear that the authors are aware of these other methods. The published paper that is closest to the present manuscript is Satulovsky, Liu and Wang (doi.org/10.1529/biophysj.107.117002, which should also be cited) which also considered extensions of points along the membrane and also showed how different components could lead to vastly different morphologies.
So, what is new here? There are a few things which argue for publication.

1. First, the technique used is mathematically rigorous as well as being fairly intuitive.
2. The manuscript demonstrates well how each of the three terms contributes to the resulting morphological changes and to ensuing motility patterns. It is well written and has high attention to detail.
3. The model output generates very realistic looking morphologies and cell tracks.
4. Furthermore, and this is probably its best feature, the existence of a python-based toolbox would be a great addition to the research field.

   We thank the reviewer for the positive evaluation of our work and for suggesting additional references. Both references are now cited within the Introduction section.

   Changes to the manuscript (Introduction):

   Usually, the cell contour is parametrized by the contour arc length [1–3], or, alternatively, by polar coordinates relative to the center of mass [4]. A comparison of the different model approaches mentioned above can be found in [5–8].

   Changes to the manuscript (Introduction):

   The second two components are mathematically well-defined geometric flows initiating contour retractions: the area-preserving curve-shortening flow (APCSF) to regularize the shape/arc length of the contour; and a further flow introduced as area adjustment flow (AAF) which expands/shrinks the cell contour with respect to a specified reference area. For more information on the APCSF, see [9–11]. Based on the interplay of the above components, the model defines the formation of protrusions as well as retractions. It is therefore linked to other mechanistic models evolving the cell contour as an elastic object in time and space [4].

Major comments:
There are some concerns and limitations, however.

1. Some of the other existing techniques (FEM, level sets and phase field) can readily handle three-dimensional shapes, and I don't particularly see how the techniques here could be extended to this more realistic scenario. It is true that most of the data that we have about morphology is based on two-dimensional images, and that most publications only provide simulations in two dimensions. However, the latter is usually done for computational reasons, rather than any specific theoretical limitation. Perhaps the title should be adjusted to mention this.

We thank the reviewer for raising this point. A more detailed rationale for the choice of a 2D modeling approach is now presented in the introduction section. We also added "in two dimensions" to the title.

Changes to the manuscript (Title):

Three-component contour dynamics model to simulate and analyze amoeboid cell motility in two dimensions

Changes to the manuscript (Introduction):

Since the majority of cell motility data rely on two-dimensional microscopy images, we have also chosen a 2D modeling approach. The theoretical framework of a 2D model, including the parametrization and propagation of the cell membrane, is much easier to handle and, intuitively, better to understand. Note that an extension of our approach to a 3D model is not straightforward and requires substantial conceptual improvements. A solid theoretical foundation of the 2D case can also serve as a starting point for the extension to 3D. Finally, a 2D model is advantageous due to its computational costs and feasibility.

2. These other existing methods can also deal more readily with biochemical reaction-diffusion models, and can then be used to infer somethings about the biological state of the cell. For example, by altering feedback strengths in excitable models used to generate protrusions, oscillatory and fan-shaped morphologies were generated in Miao et al. (doi: 10.1038/ncb3495). These various feedback loops could then be mapped into specific biochemical components (Miao, doi: 10.15252/msb.20188585). The present model can also generate the fan-shaped morphologies, but it is hard to understand in the context of the biology. So, of what use would it be to an experimentalist. This should be addressed. Incidentally, the original Miao paper that introduced the fan shapes should probably be mentioned when fan-shaped cells are first considered, as this phenotype is probably not well known outside of the Dictyostelium community and so it may not be clear.

We thank the reviewer for this very valuable feedback. We better clarified our long-term goal of a data-driven model to analyze cell motility on an intracellular level by inferring relevant biomarkers such as the F-actin density.

Changes to the manuscript (Introduction):

In our long-term quest for a quantitative, data-driven model of amoeboid motility, we want to use common biomarkers associated with the membrane and the cell cortex as direct inputs in our model and evaluate the predicted contour dynamics with experimental measurements. This way, the model acts as a computational bridge between the driving forces of cell motility, related to the F-actin density, and the resulting translocation of the cell membrane, described by the local motion of the cell contour.

Changes to the manuscript (Discussion):

In the long term, our aim is to add a biochemical component to our model by using the F-actin density as an input of the protrusion component. This way, our model can be potentially used to infer biological insights on an intracellular level, which is, however, beyond the scope of this paper. As a first step in this direction, we compared the inferred protrusion component of experimental cell tracks with the underlying F-actin density, laying the ground for such a data-driven approach.

Furthermore, both suggested references have been added to the article.

Changes to the manuscript (Introduction):

Reaction-diffusion models have been used to describe the self-organized polarization of the cell in the presence of chemoattractants [12–16], and the occurrence of intracellular waves and oscillations [17–21].

Changes to the manuscript (Discussion):

In the following, we demonstrate how our model can be used to simulate realistic cell tracks. Then, we analyze experimental cell tracks (*D. discoideum*) by inferring the model-based protrusion and retraction components for two different locomotion types: the amoeboid and a so-called fan-shaped type, see [20, 22, 23] for more details.

3. As stated by the authors, the focus is only on "lobopodia" – this limitation should probably be stated in the abstract at least.

   We thank the reviewer for this suggestion. In the abstract, it is already mentioned that the model is able to produce qualitatively different contour dynamics. Since the terminology of lobose pseudopods or lobopodia is rather new and less common in other articles, we prefer to keep the abstract as it is.

   However, we changed the misleading formulation of this phrase in the introduction since our model is able to produce protrusion dynamics of different kinds, e.g., lobopodia, lamellipodia as well as others.

   Changes to the manuscript (Introduction):

   The simulated contour dynamics within this work possess protrusions mostly resembling lobopodia, i.e., cylindrical and finger-shaped protrusions, and lamellipodia, i.e., protrusions with a flat and broader structure [24]. However, the model is not limited to these two protrusion types due to the versatile parameter regime to control the size, speed, duration, and number of protrusions.

Minor points:

1. In the discussion around line 200, the issue of self-intersections is considered. Is the model guaranteed not to generate these? That wasn't clear.

   We thank the reviewer for raising this point. The issue of self-intersections and the regularizing effect of the APCSF is now addressed.

   Changes to the manuscript (Methods):

   In comparison, the contour dynamics in panel (C) are much smoother due to the regularizing effect of the APCSF. Due to the stochastic nature of our model, the occurrence of self-intersections during the evolution of the contour can not be ruled out. However, the APCSF eliminates noise-induced self-intersections over time.

   Changes to the manuscript (Methods):

   As a consequence, the APCSF evolves every contour to a circle of the same area, minimizing the contour arc length to $L(t) \xrightarrow{t\to\infty} 2\sqrt{\pi A(0)}$ while maintaining its area. Of note, under the APCSF, a contour with self-intersections is also evolved into a circle. However, in this case, the area of the contour is not well-defined and the area-preserving property of the APCSF should be viewed with caution.

   Changes to the manuscript (Results):

   As mentioned earlier, due to the stochastic nature of our model, noise-induced self-intersections cannot be ruled out explicitly, but get eliminated over time under the influence of the APCSF. However, in all our simulation studies, we did not observe self-intersections at any time.

2. Figure S6. The kymographs in the lambda=1 and lambda=10 cases (left column) seemed identical to me. It could be that this is a mistake, or that they are just very similar.

The kymographs are indeed very similar. This is already mentioned in the results section: “Again, for the intermediate cases $\lambda_{reg} \in [0.1, 10]$, we noticed only very few differences in all shown kymographs. However, because of small changes of the contour mappings at each time step, the overall direction of the cell track can vary over time.”

3. The description (line 706 as well as the Abstract) that contour dynamics are ”hardly distinguishable from experimental data” is maybe a little overstated without some statistical validation. They certainly look realistic, but the statement could be toned down a bit.

   We thank the reviewer for this suggestion. We made adjustments to the respective statements.

   Changes to the manuscript (Abstract):

   The model is used to generate contour data for a variety of qualitatively different, e.g., polarized and non-polarized, cell tracks that visually resemble experimental data very closely.

   Changes to the manuscript (Introduction):

   The second main goal of our model is to simulate a variety of quantitatively different and realistic contour dynamics producing cell tracks that visually resemble experimental data very closely.

   Changes to the manuscript (Discussion):

   First, we have shown that our model is capable of generating a variety of cell tracks with different spatio-temporal patterns. We simulated non-polarized as well as polarized contour dynamics that look realistic and remain stable even for longer time periods.

Reviewer #2 (Remarks to the Author):

The manuscript entitled "Three-component contour dynamics model to simulate and analyze amoeboid cell motility" by D. Schindler et al. presents a mathematical model to study amoeboid cell motility dynamics. The proposed model determines the temporal evolution of the cell membrane by introducing three different model components: membrane protrusions driven by a Hawkes process, and two deterministic terms that regularize membrane arc-length and membrane area. The authors explore the influence of model parameters on cell contour dynamics and quantify the dependence of the chosen parameters on cell migration motility, persistence and mean squared displacements. They also apply the model to analyze existing cell migration experimental data.
A biological cell migrates by exchanging momentum with its microenvironment. However, the authors are not accounting for any mechanical interactions with the extracellular space. The extracellular environment simply does not exist in their model. Therefore, their model results on cell migration speeds, persistence or cell displacements are meaningless. Due to its limitations, the proposed cell migration model is incapable of quantifying cell migration capabilities.

> We agree with the reviewer that our model is a highly simplified representation of a migrating cell, where the interaction with the extracellular environment is not explicitly taken into account. However, we respectfully disagree with the reviewer's claim that this renders our model useless. The effect of the extracellular environment is incorporated indirectly as part of the protrusion component of our model, such that different properties of the environment that affect the morphodynamics of the protrusions correspond to different parameter regimes of the underlying Hawkes process. As the purpose of our model is not to study details of the cell substrate interactions, we prefer this simplification. In this way, we avoid an additional layer of complexity that would be required to explicitly include the mechanical properties of the environment and cell adhesion forces. Note also that this is a common approach in the field, adopted for example by many phase field models of cell migration. To address this concern, we now include this aspect in the Introduction and the Methods section regarding the Hawkes process.
>
> Changes to the manuscript (Introduction):
>
> In this context, we show that the Hawkes process, due to its self-exciting nature, is suitable to produce a cascade of protrusion events to ensure a persistent cell migration. Key motility characteristics, i.e., duration, size, and number of these noise-induced protrusions, can be separately adjusted with different parameters, incorporating the individual cell behavior as well as differences in the extracellular environment.
>
> Changes to the manuscript (Methods):
>
> Based on the underlying choice of parameters, different motility characteristics can be adjusted with our model:
>
> - the general movement speed by w_{prot},
> - the number of protrusions by λ_0 and α,
> - the duration of protrusions by β,
> - the size of protrusions (many small protrusions vs. a single large protrusion) by κ_M,
> - membrane fluctuation vs. creation of pseudopods with a substantial movement of the center of mass by α,
> - non-polarized vs. polarized contour dynamics by r_{pol}.
>
> Of note, the parameter regime of our model incorporates both, the individual cell behavior as well as differences in the extracellular environment.

Also, the effect of the three model components on cell shape dynamics are easy to predict: the Hawkes protrusion term creates membrane protrusions in a stochastic manner, the line tension-like term tends to minimize arc length and the pressure-like term tends to minimize cell surface area. For the reasons mentioned above, the study does not make substantial contributions to the field of cell migration.

> We agree that the three components of our model are chosen in an intuitive and straightforward manner. The underlying mechanisms are common ingredients of many cell motility models, see for example the widely used two-dimensional phase field models that include similar mechanistic components, albeit in a mathematically different framework [25, 26]. We see this as an advantage of our model, as our main goal is to develop a description of reduced complexity, where each model component is intuitively comprehensible and separately adjustable. This ensures a fast and efficient estimation of model parameters that have a clear-cut role in the contour dynamics. In the future, this may help to establish meaningful quantitative classifications of different motility modes and will allow for simulating contour dynamics with desired motility characteristics. We, therefore, see the existence of clearly separated and easily predictable model components as a strength of our model.

References

[1] Bosgraaf L, Van Haastert PJM. Navigation of Chemotactic Cells by Parallel Signaling to Pseudopod Persistence and Orientation. PLoS ONE. 2009;4(8):e6842. doi:10.1371/journal.pone.0006842.

[2] Barnhart EL, Lee KC, Keren K, Mogilner A, Theriot JA. An Adhesion-Dependent Switch between Mechanisms That Determine Motile Cell Shape. PLoS Biology. 2011;9(5):e1001059. doi:10.1371/journal.pbio.1001059.

[3] Driscoll MK, McCann C, Kopace R, Homan T, Fourkas JT, Parent C, et al. Cell Shape Dynamics: From Waves to Migration. PLoS Computational Biology. 2012;8(3):e1002392. doi:10.1371/journal.pcbi.1002392.

[4] Satulovsky J, Lui R, Wang Yl. Exploring the Control Circuit of Cell Migration by Mathematical Modeling. Biophysical Journal. 2008;94(9):3671–3683. doi:10.1529/biophysj.107.117002.

[5] Holmes WR, Edelstein-Keshet L. A Comparison of Computational Models for Eukaryotic Cell Shape and Motility. PLoS Computational Biology. 2012;8(12):e1002793. doi:10.1371/journal.pcbi.1002793.

[6] Danuser G, Allard J, Mogilner A. Mathematical Modeling of Eukaryotic Cell Migration: Insights Beyond Experiments. Annual Review of Cell and Developmental Biology. 2013;29(1):501–528. doi:10.1146/annurev-cellbio-101512-122308.

[7] Buttenschön A, Edelstein-Keshet L. Bridging from single to collective cell migration: A review of models and links to experiments. PLOS Computational Biology. 2020;16(12):e1008411. doi:10.1371/journal.pcbi.1008411.

[8] DiNapoli KT, Robinson DN, Iglesias PA. Tools for computational analysis of moving boundary problems in cellular mechanobiology. WIREs Mechanisms of Disease. 2021;13(4):1–33. doi:10.1002/wsbm.1514.

[9] Gage M. On an area-preserving evolution equation for plane curves. In: DeTurck D, editor. Nonlinear Problems in Geometry. vol. 51 of Contemporary Mathematics. American Mathematical Society; 1986. p. 51–62.

[10] Mäder-Baumdicker E. The area preserving curve shortening flow with Neumann free boundary conditions. Geometric Flows. 2015;1(1):1–57. doi:10.1515/geofl-2015-0004.

[11] Mäder-Baumdicker E. Singularities of the area preserving curve shortening flow with a free boundary condition. Mathematische Annalen. 2018;371(3-4):1429–1448. doi:10.1007/s00208-017-1637-9.

[12] Shibata T, Nishikawa M, Matsuoka S, Ueda M. Modeling the self-organized phosphatidylinositol lipids signaling system in chemotactic cells based on quantitative image analysis. Journal of Cell Science. 2012;125(21):5138–5150. doi:10.1242/jcs.108373.

[13] Shibata T, Nishikawa M, Matsuoka S, Ueda M. Intracellular Encoding of Spatiotemporal Guidance Cues in a Self-Organizing Signaling System for Chemotaxis in Dictyostelium Cells. Biophysical Journal. 2013;105(9):2199–2209. doi:10.1016/j.bpj.2013.09.024.

[14] Taniguchi D, Ishihara S, Oonuki T, Honda-Kitahara M, Kaneko K, Sawai S. Phase geometries of two-dimensional excitable waves govern self-organized morphodynamics of amoeboid cells. Proceedings of the National Academy of Sciences. 2013;110(13):5016–5021. doi:10.1073/pnas.1218025110.

[15] Eidi Z, Mohammad-Rafiee F, Khorrami M, Gholami A. Modelling of Dictyostelium discoideum movement in a linear gradient of chemoattractant. Soft Matter. 2017;13(44):8209–8222. doi:10.1039/C7SM01568B.

[16] Camley BA, Zhao Y, Li B, Levine H, Rappel WJ. Crawling and turning in a minimal reaction-diffusion cell motility model: Coupling cell shape and biochemistry. Physical Review E. 2017;95(1):012401. doi:10.1103/PhysRevE.95.012401.

[17] Beta C, Kruse K. Intracellular Oscillations and Waves. Annual Review of Condensed Matter Physics. 2017;8(1):239–264. doi:10.1146/annurev-conmatphys-031016-025210.

[18] Shenderov AD, Sheetz MP. Inversely correlated cycles in speed and turning in an ameba: an oscillatory model of cell locomotion. Biophysical Journal. 1997;72(5):2382–2389. doi:10.1016/S0006-3495(97)78883-0.

[19] Camley BA, Zhao Y, Li B, Levine H, Rappel WJ. Periodic Migration in a Physical Model of Cells on Micropatterns. Physical Review Letters. 2013;111(15):158102. doi:10.1103/PhysRevLett.111.158102.

[20] Miao Y, Bhattacharya S, Edwards M, Cai H, Inoue T, Iglesias PA, et al. Altering the threshold of an excitable signal transduction network changes cell migratory modes. Nature Cell Biology. 2017;19(4):329–340. doi:10.1038/ncb3495.

[21] Miao Y, Bhattacharya S, Banerjee T, Abubaker-Sharif B, Long Y, Inoue T, et al. Wave patterns organize cellular protrusions and control cortical dynamics. Molecular Systems Biology. 2019;15(3):1–20. doi:10.15252/msb.20188585.

[22] Asano Y, Mizuno T, Kon T, Nagasaki A, Sutoh K, Uyeda TQP. Keratocyte-like locomotion in amiB -null Dictyostelium cells. Cell Motility and the Cytoskeleton. 2004;59(1):17–27. doi:10.1002/cm.20015.

[23] Moldenhawer T, Moreno E, Schindler D, Flemming S, Holschneider M, Huisinga W, et al. Spontaneous transitions between amoeboid and keratocyte-like modes of migration. Front Cell Dev. 2022;10:898351. doi:10.3389/fcell.2022.898351.

[24] Petrie RJ, Yamada KM. At the leading edge of three-dimensional cell migration. Journal of Cell Science. 2012;125(24):5917–5926. doi:10.1242/jcs.093732.

[25] Shao D, Rappel WJ, Levine H. Computational Model for Cell Morphodynamics. Physical Review Letters. 2010;105(10):108104. doi:10.1103/PhysRevLett.105.108104.

[26] Alonso S, Stange M, Beta C. Modeling random crawling, membrane deformation and intracellular polarity of motile amoeboid cells. PLOS ONE. 2018;13(8):e0201977. doi:10.1371/journal.pone.0201977.
