# Supplementary material for: Three-component contour dynamics model to simulate and analyze amoeboid cell motility in two dimensions
Source: PLoS One. 2024 Jan 26;19(1):e0297511. doi: 10.1371/journal.pone.0297511 (PMC10817190; doi:10.1371/journal.pone.0297511)
Supplement: Attachment — Submitted filename: Review Report PONE-D-23-25151.pdf [file pone.0297511.s021.pdf]

This article presents the authors' remarkable modeling efforts toward accounting for amoeba cell motility. It uses a 2D framework focusing on contour dynamics and consisting of stochastic simulations and other deterministic driving mechanisms. It also offers a method to fit on experimental data. This work provides a unique view of this classical problem. The authors also decided to present alternative driving stochastic processes (simple Poisson process, O-U process). This is especially helpful in the article's completeness in contrast to if they chose to present only the final successful story to make it superficially magical. However, I have non-negligible concerns about its readability, as well as some minor issues on the logics of wording. At PLOS One, objective assessment of logic and presentation is valued primarily, rather than subjective impact or popularity, so I hope the authors and editor(s) can take into account the following call-outs. Even though the piece of research is deemed rigorous and sound, It is my humble opinion that a major revision to this article fixing at least the majority of these concerns before the article can be published.

The authors should include an explicit comparison to their previous work ref [82]. Don't assume it's obvious.

I believe the accuracy of the very first few sentences is important. In "a wide variety of biomedical applications such as wound healing, cancer metastasis, and embryonic morphogenesis", all three examples especially cancer metastasis are not "biomedical applications" but rather natural "biomedical processes".

Line 106 — 108: "The theoretical framework of a 2D model, including the parametrization and propagation of the cell membrane, is much easier to handle and, intuitively, better to understand." This sentence should append "than 3D" because otherwise readers cannot know what this is comparing to before reading the next sentence which finally mentions 3D.

Line 148: the mentioned σ_{noise} never appears in any equation

Equation 6: the angled bracket likely means inner product but should be defined clearly.

After line 163, the regularization is introduced, but it's again introduced in line 187. Line 163 also comes with a λ_{reg} that comes from reference to previous work but never appears in any equation in this article. Given that a whole section later beginning at Line 450 is dedicated to this parameter, lack of a more formal introduction is suboptimal. Readers like me can hardly comprehend the preciseness of tuning this parameter knowing only a rough qualitative idea behind λ_{reg}. If the authors think λ_{reg} is not worthwhile a formal introduction because one can simply refer to previous work, then the whole section beginning at Line 450 should be moved to SI.

Figure 1: I don't immediately understand the difference between X_{prot} and f_{prot}. The distinction between the two is only made at Equation 16, but I'm still not sure why X_{prot} as only an inner expression is worth plotting alongside the full f_{prot}.

Similar to the confusion in Figure 1, on line 281—283, the authors made the distinction between "Hawkes intensity process" and "Hawkes process itself", which to me sounds quite confusing. Isn't the intensity dependent on past process history (itself) and dictates future event probability? The wording needs to be more mathematically standardized.

Line 286: what is the meaning of “general” movement speed (please also clarify this term in line 778)? Also, correct me if I’m wrong, but I think this is the first time this symbol of ω_{prot} is discussed. Looking at past referee’s report, it seemed that this line was newly add at a revision, but this makes discussion of ω_{prot} happen before its definition point.

Line 324: “the area adjustment is weaker for contour segments, which are closer to the center of mass”. The comma before “which” should be deleted because of English grammar rules (see https://academicguides.waldenu.edu/writingcenter/grammar/clauses).

Figure 2: it seems to me that Figure 2 is logically similar to Figure 1, since both are testing with one component at a time. The authors needs to explain what is the difference between the two.

Line 338: “Usually, this is done by, …, however”. This is again confusing. The authors need to explicitly point out this work is not doing the “usual” way.

Figure 7: Is the fluorescence imaging in panel B and D the same, i.e. both are imaging F-actin density? Later in Line 593, the text jumps directly to the saturation issue, but this is the first place for me to get the answer to this confusion. The definition of each plot needs to come before discussion of its saturation issue.

Line 604: what is the “main” characteristics? I understand the authors are trying to say Figure 7 panel D is more cluttered than C, but it could be due to color scale, nonlinear relationship between C and D, etc., and not necessarily because C has more details.

Line 605: please delete “a” before “very few”.

Line 606: “We thus conclude that the missing retractive regions are successfully captured by the AAF and the APCSF.” This is not rigorous to conclude AAF and the APCSF are responsible for the negative change based on that Protrusion is mostly positive. It’s better to show summing up AAF and APCSF and protrusion equals the local membrane motion. That is done in S14Fig and Line 614, but at the point of line 606, the logic of expression is not optimal.

Line 634: this is the same problem with λ_{reg}: definition of ω_f and relative weights reside in SI but its quantitative analysis remains in main text. This approach doesn’t make sense to me. It is as if saying to readers: just trust me when I tell you the effect of varying this parameter, and you cannot know why until you go to SI. Overall, it does’t make sense to me to have a detailed paragraph in the main text that relies heavily on SI. Definition of quantities, quantitative analysis, most supporting plots should be together. For example, they could all be in SI, and the authors just mention briefly in main text that they analyzed influence of a priori model weights in SI, and mention the conclusion that the influence is minor.

Line 676: a major advantage of this model is the ability to accommodate a variety of contour dynamics, and even classify them. It’s therefore much better to highlight more than one example of experimental cell tracks in main text, rather than keep referring to Supplemental Figures in main text.

Line 833: I disagree based on the text that the approach is “fully” automated. It is indeed automated, but we have seen the necessity to make a priori selection of model weights. In year 2023, a typical example of “fully” automated computer program is chatGPT. For example, users don’t need to make a priori hyper-parameter choices when using chatGPT.
