# Supplementary material for: Three-component contour dynamics model to simulate and analyze amoeboid cell motility in two dimensions
Source: PLoS One. 2024 Jan 26;19(1):e0297511. doi: 10.1371/journal.pone.0297511 (PMC10817190; doi:10.1371/journal.pone.0297511)
Supplement: Attachment — Submitted filename: response_to_reviewers_plos_one.pdf [file pone.0297511.s022.pdf]

Response to the reviewers' comments

Preface

We highly appreciated the constructive comments and remarks by the reviewers and the editor. We have addressed all comments and remarks and revised the manuscript accordingly. In the point-by-point response below, answers to the reviewers' comments are highlighted in blue and changes to the manuscript in green.

Finally, we would like to thank the reviewers and associated editor for their time and consideration.

Contents

Reviewer #1 (Remarks to the Author): 2
Reviewer #2 (Remarks to the Author): 7

Reviewer #1 (Remarks to the Author):

The authors have presented a model with which they can simulate cell motility with a clear relationship between the parameters and the resulting motility. The model is capable of displaying different motilities, resembling amoeboid and fan shaped motility. Their model can also be used to fit on experimental cell tracks and be used to estimate the underlying parameters for such tracks. From such fits, the model component for protrusion correlates with the fluorescence of actin, which is a nice result, as the actin fluorescence seems indicative of protrusions and are not considered in the fitting process.

> We thank the reviewer for the positive evaluation of our model and the very helpful feedback on our manuscript. In the following, the feedback is addressed point by point.

Major points:

1. The authors have not mentioned the Cellular Potts model as a model for cell motility in their introduction.
   I think the Act model extension (https://doi.org/10.1371/journal.pcbi.1004280) deserves mentioning. Furthermore, I think the methods presented might be interesting to use as a comparison tool between other models, such as the CPM, and/or experimental data.

   Regarding the usage of cellular Potts models to describe amoeboid motility, we added two references to the Introduction section.

   Additionally, extensions of the cellular Potts model have been successfully used to reproduce amoeboid and keratocyte-like movements [1, 2].

   The possibility to use our model as a comparison tool for other existing models is now addressed in the Introduction and Discussion section

   Introduction:

   By inferring the above characteristics, our model can also be used to identify differences between cells, to classify them, and as a general comparison tool for other existing cell motility models. In this manner, we envision our model to be applied to different organisms, experimental setups, and artificial contour dynamics generated by different models, which is, however, beyond the scope of this paper.

   Discussion:

   Due to a fast and straightforward parameter estimation and a fully automatized approach to infer protrusion and retraction characteristics, the model can be used to analyze experimental cell tracks on the individual level, classifying them, and as a general comparison tool for experimental contour data as well as contour dynamics generated by different cell motility models.

2. In equation 11, cell polarization is implemented. As far as I've understood this, the polarization angle is fixed. In what ways could Eq. 11 be adapted to allow for things like repolarization, for instance upon collision with other cells or obstacles?

   In its current form, the polarization angle is indeed fixed. However, due to the usage of a kernel function ($\tilde{k}_{r_{\mathrm{pol}}}(\theta, \theta')$), the polarization can be easily adjusted with respect to other cells, obstacles or external cues. For the sake of simplicity, we have chosen a fixed polarization which allows us to draw realizations of the Hawkes process for the entire time span in advance. This is now better clarified in the manuscript.

Methods:

As background intensity function, we now define

$$\mu(\theta) = \lambda_0 \frac{\tilde{k}_{r_{\text{pol}}}(\theta, \pi)}{\int_0^{2\pi} \tilde{k}_{r_{\text{pol}}}(\theta, \pi)\, d\theta},$$

with background rate $\lambda_0 > 0$ and $\tilde{k}_{r_{\text{pol}}}$ as in Eq 10 with $0 \leq r_{\text{pol}} < 1$. For the non-polarized case $r_{\text{pol}} = 0$, the background intensity simplifies to $\mu = \frac{\lambda_0}{2\pi}$. For $r_{\text{pol}} > 0$, polarization takes place with a local maximum at $\theta = \pi$, and local minima at $\theta = 0$ and $\theta \to 2\pi$. For the sake of simplicity, the polarization is fixed for the entire time span and also with respect to the local position on the contour ($\theta' = \pi$). One could add a time dependence to the background intensity with a changing cell front, i.e., with a polarization angle θ' in Eq 10 varying over time and depending on the proximity of external cues, repelling contours, or obstacles. In this case, however, the Hawkes process needs to be realized sequentially over time while evolving the cell contour. In contrast, in our test case with a locally fixed and time-independent polarization, the Hawkes process can be realized in advance before the contour is propagated.

3. Lines 543-552: I think it is also interesting to report the persistence time alongside the diffusion coefficient, which can easily be found by fitting Fürth's equation. Regarding the direct relation between parameters and motility, I wonder if this model is able to display the universal coupling between speed and persistence (https://doi.org/10.1016/j.cell.2015.01.056), or whether this is fully decoupled in this model? Maybe the authors would like to discuss on this?

   We thank the reviewer for this suggestion. The persistence time and mean instantaneous speed are now included. Based on the computed values for the polarized case and non-polarized case, we deduce that the universal coupling between cell persistence and cell speed is also displayed by our model.

   In addition to the diffusion coefficient, a persistence time P can be computed by fitting the mean squared displacements (MSD) to Fürth's formula (dimension n=2):

$$\text{MSD}(t) = 2nD(t - P(1 - e^{-t/P})),$$

   a standard reference model, where the trajectories are based on an Ornstein-Uhlenbeck process [3, 4]. By doing so, we determined a mean persistence time of 76.8min for the non-polarized case and 113.5min for the polarized case. The mean instantaneous speed of the center of mass is $3.9\mu m$/min for the non-polarized case, whereas it is more than doubled for the polarized case ($8.7\mu m$/min). This observation is in accordance with previous studies where the existence of a universal coupling between cell persistence and cell speed has been shown [4].

Minor points:

1. Fig 2: some parameter values are given, are the remaining as stated in table 1?

   Regarding the contour parametrization, the underlying parameters are now included in the Figure caption. Furthermore, we added a phrase to the corresponding paragraph clarifying that the underlying contour propagation in these examples takes place without any kind of VM remapping/regularizing flows.

   Caption Fig 2:

   Comparison of the three model components: f_{prot}, f_{APCSF}, and, f_{AAF}. (A) Protrusion component driven by a stochastic process (generated sample shown

in top right corner). The reference point $\theta = 0$ is highlighted as blue dot. **(B, C)** APCSF and AAF with final states displayed in corresponding top right corners. **(D-F)** Example contour propagated individually by only one component for a time span of $1s$. Regarding the contour parametrization, the reference area is given by $A_{\text{ref}} = 60\mu m^2$ with GPR hyperparameters $r_{\text{cont}} = 0.4$ and $\sigma_{\text{noise}} = 0.01$.

Methods:

Finally, in panels (D-F), we display test scenarios of simulated contour dynamics each based on a single component only. In panel (D), we observe a steadily growing contour since the f_{prot} is the only active component. Under the APCSF (panel (E)) the contour evolves into a circle by expanding concave parts and shrinking convex parts of the contour. In the case of AAF being the only active component, we observe a shape-preserving shrinkage of the contour. In this case, the reference area ($A_{\text{ref}} = 60\mu m^2$) was set to be smaller than the initial contour area. Alternatively, by choosing a larger reference area, a shape-preserving growth of the contour would occur. The contours in panels (D-F) are propagated in normal direction only without any influence of VM remapping/regularizing flows.

2. Figure 5 For me it was not immediately clear that the colors in panel B and correspond to those in D and E. Also, I think that in the sentence "The same protrusion component is underlying in (B) and (B), respectively." one of the (B) is probably a (C).

   The color matching displayed in this Figure is now better clarified.

   Caption Figure 5:

   Influence of regularization parameter λ_{reg} on simulated cell tracks. (A) The impact of λ_{reg} on the virtual marker distribution described by the standard deviation of the virtual marker distance ratio σ_{VMDR} and its reciprocal. **(B, C)** Non-polarized (left) and polarized (right) cell tracks generated with varying regularization parameter $\lambda_{\text{reg}} \in \{0.01, 0.1, 10, 1000\}$. The same protrusion component is underlying in **(B)** and **(C)**, respectively. **(D, E)** Histograms of the VMDR for varying λ_{reg} corresponding to the non-polarized (left) and polarized (right) cell tracks from panels (B) and (C). **(F, G)** Kymographs of the APCSF component of all non-polarized (left) and polarized (right) cell tracks with regions of interest shown as black and white dashed boxes.

3. Fig 6: It may be good to note that n=50 in the caption of this figure.

   The overall number of simulated cell tracks is now added to the Figure caption.

   Caption Figure 6:

   Diffusion analysis of artificial cell tracks generated from a non-polarized test scenario (top row) and a polarized test scenario (bottom row). **(A, F)** For each scenario, ten out of 50 exemplary cell tracks are shown. **(B, C, G)** Center of mass trajectories (colored lines) with root mean squared displacement (grey circles) at different time points with $\Delta t = 100s$. While panel (B) and (G) are scaled equally, panel (C) is an enlarged excerpt of panel (B). **(D, H)** Corresponding MSD of these trajectories (bold black line) for medium time spans of $T = 500s$. **(E, I)** MSD for longer time spans of $T = 10000s$ with linear fit (dashed red line).

4. Figure 7: Fluorescence intensity of what fluorescent signal is being depicted in panel B?

   We added the fluorescence marker to the Figure caption and the corresponding paragraph.

   Caption Figure 7:

   Inferring protrusion component from *D. discoideum* cell track. (A) Persistently motile cell track for $T = 500s$. **(B)** Microscopy image with fluorescence intensity (mRFP tagged LifeAct as marker for filamentous actin, white to green color scheme) and segmented cell contours (red lines, every tenth shown). **(C)** Local motion kymograph showing expansions (red areas) and retractions (blue areas). **(D)** Relative fluorescence intensity with regions of high and low F-actin density displayed in red and blue, respectively. **(E)** The underlying protrusion component inferred from our model for a given set of model parameters. The resulting propagation of virtual markers from one contour to the next one is depicted as black circles in panel (B). **(F)** The contour area with predefined reference area $A_{\text{ref}} = 91.23\mu m^2$ (dashed gray line). Finally, regions of interest are displayed as black and white dashed boxes.

   Results section:

   This cell track is based on fluorescence microscopy data (mRFP tagged LifeAct, white to green color scheme), from which the cell contours are segmented (colored lines), see panel (B).

5. Line 633: I found the notation of relative weight parameters confusing at first, as they look similar to the parameter r_{cont} and r_{pol}. Consider less similar parameter symbols.

   Instead of the notations r_{prot}, r_{APCSF}, and r_{AAF} for the relative weights, we now use $w^{\text{rel}}_{\text{prot}}$, $w^{\text{rel}}_{\text{APCSF}}$, and $w^{\text{rel}}_{\text{AAF}}$. Based on the suggestion of the other Reviewer, the paragraph regarding the analysis of relative parameter weights was shortened and partly moved to the supporting information (S1 Text).

   Results:

   Impact of *a priori* parameter choices on the analysis of experimental data. The F-actin density near the membrane is often interpreted as a marker of protrusive activity. Thus, we would expect some correlation between the F-actin density and the protrusion component in our model. Since the latter is influenced by the choice of the model weights of the two other model components AAF and APCSF, the question arises to what extent the correlation depends on the *a priori* choices of w_{AAF} and w_{APCSF}.

   In S1 Text, we rewrite the model equations from Eq (16) with respect to relative model weights instead of the absolute weights w_{prot}, w_{APCSF}, and w_{AAF}. An impact analysis of the varying model weights on the inferred protrusion component is also included in S1 Text. The inferred protrusion component kymographs resulting from this impact analysis are shown in S13 Fig. For the different configurations of model weights, we computed the Pearson correlation coefficient between the protrusion component and the Fluorescence intensity of the F-actin density, see S13 Fig.

   Briefly, we observed that the protrusion component inferred by the model is correlated to the underlying F-actin density. While the protrusion component is affected significantly by the parameter choice, the impact on the Pearson correlation coefficient is minor. In the case of the estimated model weights used in Fig 7, a Pearson correlation coefficient of $\rho = 0.47$ was computed, which is close to the Pearson correlation for the best fit ($\rho = 0.49$), see S1 Text and S13 Fig for details

6. Lines 648-671 and Fig S15, please specify which correlation coefficient was used.

   In our studies, the Pearson correlation coefficient was used. This is now clarified in the corresponding paragraph and the caption of the former S15 Fig (now S13 Fig). This paragraph, also containing the analysis by relative parameter weights was shortened and partly moved to the supporting information (S1 Text).

   Lines 648-671:
   In S13 Fig, we display protrusion component kymographs for varying parameters $r_{\text{prot}} \in \{0.05, 0.5, 0.8\}$, $r_{\text{APCSF}} \in \{0.01, 0.05, 0.1\}$, and $w_f \in \{1, 5, 10, 20\}$ of the cell track from Fig 7 and the corresponding Pearson correlation coefficient between these kymographs and the fluorescence intensity/F-actin density.

   For all cases, the resulting Pearson correlation coefficients between the protrusion component and F-actin density fell within a range of $[0.32, 0.49]$.

   Since the Pearson correlation coefficient is invariant under changes in location and scale, we conclude that most of the protrusion kymographs displayed in S13 Fig differ in magnitude primarily.

   Caption Fig S13 (Former S15 Fig):
   The Pearson correlation coefficient ρ between the protrusion component and the fluorescence intensity kymograph from Fig 7D is displayed above each kymograph.

7. The order in which the supplementary figures appear in the main text is not the same as their numbering.

   We revised the manuscript regarding the ascending numbering of supplementary figures. Based on a suggestion of the other reviewer we moved the former S11 Fig and S12 Fig to the main text (now Fig 8 and Fig 9). Hence, the total number of SI Figs changed from 15 to 13.

8. Fig S1: some typos in caption

   We corrected the typos in this caption.

   Caption Figure S1:
   Fig S1. Artificial contour dynamics based on our model with a comparison of the underlying contour propagation and contour mapping. First, the contour is propagated by the model function f evaluated for an equidistant set of grid points on the contour (green dashed trajectories). Secondly, the protrusion component X_{prot} is propagated by a strongly regularized flow to avoid thinning and clustering effects of virtual markers (blue dashed trajectories). This contour mapping can then be used as an underlying coordinate system in kymograph representations.

Reviewer #2 (Remarks to the Author):

This article presents the authors' remarkable modeling efforts toward accounting for amoeba cell motility. It uses a 2D framework focusing on contour dynamics and consisting of stochastic simulations and other deterministic driving mechanisms. It also offers a method to fit on experimental data. This work provides a unique view of this classical problem. The authors also decided to present alternative driving stochastic processes (simple Poisson process, O-U process). This is especially helpful in the article's completeness in contrast to if they chose to present only the final successful story to make it superficially magical. However, I have non-negligible concerns about its readability, as well as some minor issues on the logics of wording. At PLOS One, objective assessment of logic and presentation is valued primarily, rather than subjective impact or popularity, so I hope the authors and editor(s) can take into account the following call-outs. Even though the piece of research is deemed rigorous and sound, It is my humble opinion that a major revision to this article fixing at least the majority of these concerns before the article can be published.

We much appreciated the positive evaluation of our model by the reviewer. Furthermore, we want to thank the reviewer for the very constructive and, especially, helpful feedback regarding the readability of our manuscript. In the following, we address the reviewer's concerns step-by-step:

1. The authors should include an explicit comparison to their previous work ref [82]. Don't assume it's obvious.

    We revised the first paragraph of the Methods section to clarify the differences between our previous work from [5] and our present model.

    Furthermore, we revised a paragraph in the Discussion section to highlight the differences between the current model and a previous modeling approach based on phase-field equations [6, 7].

    Introduction (Section "General notations and underlying coordinate system"): The notation and theoretical framework used in this work have been established in [5]. Primarily, this framework is used as an analysis tool of amoeboid cell motility enabling us (1) to obtain smooth contour representations from stacks of segmented microscopy images, (2) to track quantities of interest along reference points (virtual markers) between successive contours to generate so-called kymograph plots, and (3) to identify protrusion and retraction events in an automated way. The framework was applied to artificial contour dynamics as well as cell tracks of different organisms and experimental setups. The first two aspects mentioned above, i.e., the process of generating smooth contour representations and the mapping of consecutive contours in time and space, are now used as part of our novel computational model to simulate contour dynamics and to analyze experimental data by inferring key motility characteristics based on this model. For a better understanding of our model, the framework presented in [5] is summarized in this section.

    Discussion:

    In a previous approach, we used phase-field equations to model amoeboid cell motility [6, 7]. This phase-field model also consists of three terms: surface tension, volume conservation, and active tension which correspond to the three components of this work, i.e., the APCSF, the AAF, and the protrusion component, respectively. The phase-field model describes the changes of a two-dimensional biochemical component, reacting and diffusing inside the cell, and leading to a propagation of the entire cell. In contrast, the present work focuses on the propagation of a one-dimensional object only, namely the cell contour, further reducing the complexity of the model. Furthermore, the present model does not rely on

the underlying concentration of biomarkers, e.g., the F-actin density, but can be used to infer information about the protrusions of experimental cell tracks. Due to the area-regularizing effect of the AAF, the cell area is preserved within a specific range, which is similarly done by the volume conservation terms in different phase-field models [6, 8, 9]. In contrast to our previous phase-field model, where new protrusions were generated by an Ornstein-Uhlenbeck process driven by Gaussian white noise, the focus of this work is mainly on the Hawkes process due to its self-exciting nature. Finally, our phase-field model was used to study more complex processes such as cell division or the interaction and movement of multiple cells [10, 11].

2. I believe the accuracy of the very first few sentences is important. In "a wide variety of biomedical applications such as wound healing, cancer metastasis, and embryonic morphogenesis", all three examples especially cancer metastasis are not "biomedical applications" but rather natural "biomedical processes".

   We updated the Abstract with respect to this suggestion.

   Abstract:

   Amoeboid cell motility is relevant in a wide variety of biomedical processes such as wound healing, cancer metastasis, and embryonic morphogenesis.

3. Line 106 — 108: "The theoretical framework of a 2D model, including the parametrization and propagation of the cell membrane, is much easier to handle and, intuitively, better to understand." This sentence should append "than 3D" because otherwise readers cannot know what this is comparing to before reading the next sentence which finally mentions 3D.

   We updated the phrase accordingly.

   Introduction:

   The theoretical framework of a 2D model, including the parametrization and propagation of the cell membrane, is much easier to handle and, intuitively, better to understand than in 3D.

4. Line 148: the mentioned σ_{noise} never appears in any equation

   We revised the paragraph regarding the Gaussian process regression. The formula including the hyperparameter σ_{noise} is now included in the manuscript.

   Methods:

   In our case, we used a Poisson kernel as underlying covariance function

$$k_r(\theta, \theta') = \frac{1 - r^2}{1 - 2r\ \cos(\theta - \theta') + r^2}, \quad \theta, \theta' \in [0, 2\pi),\ r \in [0, 1). \tag{1}$$

   Briefly, the choice of r_{cont} in $k_{r_{\text{cont}}}(\cdot, \cdot)$ affects the rigidity and stiffness of the resulting contour. Furthermore, an additional noise parameter $\sigma_{\text{noise}} > 0$ specifies the deviation between the initial segmentation points and the regression function obtained from the GPR with covariance structure:

$$\text{Cov}(\theta, \theta') = k_{r_{\text{cont}}}(\theta, \theta') + \sigma^2_{\text{noise}}\delta(\theta - \theta'),$$

   with $\delta(\cdot)$ denoting the Dirac delta function.

5. Equation 6: the angled bracket likely means inner product but should be defined clearly.

The brackets' meaning is now clarified after the first appearance.

Methods:

The dynamics of the contour itself are obtained by propagating an initially equidistant set of contour points for short time periods and with respect to the normal vector field

$$\left\langle \frac{\partial \Phi(t,\theta)}{\partial t}, \vec{n}(t,\theta) \right\rangle = f(t,\theta), \tag{2}$$

at time $t \in [0, T]$, normalized arc length coordinate $\theta \in [0, 2\pi)$, and $\langle \cdot, \cdot \rangle$ denoting the inner product.

6. After line 163, the regularization is introduced, but it's again introduced in line 187. Line 163 also comes with a λ_{reg} that comes from reference to previous work but never appears in any equation in this article. Given that a whole section later beginning at Line 450 is dedicated to this parameter, lack of a more formal introduction is suboptimal. Readers like me can hardly comprehend the preciseness of tuning this parameter knowing only a rough qualitative idea behind λ_{reg}. If the authors think λ_{reg} is not worthwhile a formal introduction because one can simply refer to previous work, then the whole section beginning at Line 450 should be moved to SI.

   We agree with the reviewer that the regularization parameter λ_{reg} should be formally introduced as part of an equation. We, therefore, included an equation describing the influence of λ_{reg} on the resulting virtual marker mapping. Furthermore, we moved the introduction of the regularization parameter λ_{reg} (former line 163) to the latter paragraph (former line 187) to avoid confusion during the reading.

   Methods:

   In this discrete setting, by using regularized flows as in [5], a mapping $\phi_{k,\lambda_{\text{reg}}}$ between consecutive contours can be determined by solving the following optimization problem:

$$\phi_{k,\lambda_{\text{reg}}} = \operatorname{argmin}_{\phi_k} F_k[\phi_k] + \lambda_{\text{reg}} U_k[\phi_k], \quad \lambda_{\text{reg}} \geq 0,$$

   with cost functions $F_k[\phi_k]$ and $U_k[\phi_k]$ defined by:

$$F_k[\phi_k] \simeq \frac{1}{N\delta t^2} \sum_{i=0}^{N-1} \left\| \Phi_{k+1}(\theta_{k+1,i}) - \Phi_k(\theta_{k,i}) \right\|^2,$$

$$U_k[\phi_k] \simeq N \sum_{i=0}^{N-1} \left| \theta_{k+1,i+1} - \theta_{k+1,i} \right|^2.$$

   The first term F_k is described by the sum over the lengths of all virtual marker trajectories from one contour to the consecutive contour, whereas the second term U_k takes the sum over coordinate differences of neighboring virtual markers on the consecutive contour. Depending on a regularization parameter $\lambda_{\text{reg}} \geq 0$, this family of contour flows includes the two extreme cases: either enforcing shortest VM trajectories between contours ($\lambda_{\text{reg}} = 0$) or preserving equal distances between neighboring VMs for every time step ($\lambda_{\text{reg}} \gg 0$). For the continuous formulation of these regularized flows, see [5].

7. Figure 1: I don't immediately understand the difference between X_{prot} and f_{prot}. The distinction between the two is only made at Equation 16, but I'm still not sure why X_{prot} as only an inner expression is worth plotting alongside the full f_{prot}.

It is true that f_{prot} does not differ significantly from the underlying stochastic process X_{prot}. However, by introducing X_{prot}, we want to emphasize the possibility of inserting different kinds of stochastic processes (Hawkes process, Poisson process, Ornstein-Uhlenbeck process) into our model. Furthermore, by plotting the underlying stochastic process, we want to highlight that the stochastic process is directly translated into the forward movement of the contour. Nevertheless, we included additional phrases clarifying the difference between these two quantities and the possibility to insert different kinds of stochastic processes into our model.

Methods:

In Fig 1, simulated contour dynamics are shown based on: the protrusion component f_{prot} only, f_{prot} combined with f_{APCSF}, f_{prot} combined with f_{AAF}, and a combination of all three components. In the first row (panel A), four different samples drawn from the same stochastic process X_{prot} are displayed. The magnitude of this stochastic process is then directly translated into the protrusion component f_{prot}, see panel (B). The first two cases (panels (B) and C)) lead to significantly growing contours since the area adjustment is absent.

Methods:

In this section, we formulate our contour dynamics model based on the three components: a protrusion component based on a stochastic process X_{prot}, e.g., a Hawkes process defined as in Eq 13, the APCSF from Eq 7, and the AAF from Eq 8.

Methods:

Based on the first equation, the protrusion component f_{prot} is mainly affected by X_{prot} for which we can insert different stochastic processes, e.g., a Hawkes process, an Ornstein-Uhlenbeck process, or a Poisson point process.

Methods:

First, in Fig 1, we have shown the interplay of the three model components and why all three components are required to produce stable and realistic contour dynamics. Now, separately generated contour dynamics, each being based on one model component only, are shown in Fig 2. In panel (A), the protrusion component is displayed with a generated sample of the underlying stochastic process X_{prot} in the top right square. In this example, X_{prot} is defined as correlated noise on a unit circle and is mapped onto the contour with respect to the contour arc length and a reference point $\theta = 0$ (blue dot).

8. Similar to the confusion in Figure 1, on line 281—283, the authors made the distinction between "Hawkes intensity process" and "Hawkes process itself", which to me sounds quite confusing. Isn't the intensity dependent on past process history (itself) and dictates future event probability? The wording needs to be more mathematically standardized.

We changed the wording as suggested.

Methods:

This component is based on a stochastic process, e.g., a Hawkes process or an Ornstein-Uhlenbeck process, and should be strictly positive since it describes the formation of protrusions only.

Methods (subsection title):

Intensity of a Hawkes process as protrusion component

Methods (former line 281):

For the sake of simplicity, we choose $c_s = 1s$ and the same kernel function as above, i.e., $\tilde{g}(t,\theta) \equiv g(t,\theta)$. Hence, we can rewrite the protrusion process X_{prot} in terms of the intensity $\lambda(t,\theta)$ of a Hawkes process realization, defined as in Eq (9):

$$X_{\text{prot}}(t,\theta) = \frac{c_s}{\text{VMDR}(t,\theta)} \left(\lambda(t,\theta) - \mu(\theta)\right),$$

to highlight that X_{prot} directly resembles the intensity of the Hawkes process and not the underlying realization of point events or the Hawkes process itself.

9. Line 286: what is the meaning of "general" movement speed (please also clarify this term in line 778)? Also, correct me if I'm wrong, but I think this is the first time this symbol of w_{prot} is discussed. Looking at past referee's report, it seemed that this line was newly add at a revision, but this makes discussion of w_{prot} happen before its definition point.

We removed the expression "general movement speed" in Line 286 and Line 778. Instead, we included a new phrase in this paragraph to point out that the velocity of contour changes can be adjusted with the three model weights.

Discussion (former line 286):

Based on the underlying choice of parameters, different motility characteristics can be adjusted with our model:

- the number of protrusions by λ_0 and α,
- the duration of protrusions by β,
- the size of protrusions (many small protrusions vs. a single large protrusion) by κ_M,
- membrane fluctuation vs. creation of pseudopods with a substantial movement of the center of mass by α,
- non-polarized vs. polarized contour dynamics by r_{pol}.

Furthermore, the velocity of contour changes during the creation of protrusions and retractions can be adjusted with additional model weights which are introduced in the following section. Of note, the parameter regime of our model incorporates both, the individual cell behavior as well as differences in the extracellular environment.

Discussion (former line 778):

Here, our model differs substantially due to its dependency on three components only and a low number of parameters: 4 model parameters and 4–5 additional parameters regarding the stochastic process. Desired motility characteristics such as the number, duration, and size of protrusions, the velocity of the evolving contour, or the cell polarization can be easily achieved based on an appropriate specification of the underlying parameter regime.

10. Line 324: "the area adjustment is weaker for contour segments, which are closer to the center of mass". The comma before "which" should be deleted because of English grammar rules (see https://academicguides.waldenu.edu/writingcenter/grammar/clauses).

We updated the phrase accordingly.

11. Figure 2: it seems to me that Figure 2 is logically similar to Figure 1, since both are testing with one component at a time. The authors needs to explain what is the difference between the two.

The difference between both figures is now better clarified. The first figure describes the interplay of the model components when used in different configurations, e.g., $f_{\text{prot}} + f_{\text{APCSF}}$ or $f_{\text{prot}} + f_{\text{AAF}}$. In contrast, contour dynamics that are generated with one model component only and the underlying mechanics are shown in Figure 2.

Methods:

First, in Fig 1, we have shown the interplay of the three model components and why all three components are required to produce stable and realistic contour dynamics. Now, separately generated contour dynamics, each being based on one model component only, are shown in Fig 2. In panel (A), the protrusion component is displayed with a generated sample of the underlying stochastic process X_{prot} in the top right square. In this example, X_{prot} is defined as correlated noise on a unit circle and is mapped onto the contour with respect to the contour arc length and a reference point $\theta = 0$ (blue dot).

12. Line 338: "Usually, this is done by, ..., however". This is again confusing. The authors need to explicitly point out this work is not doing the "usual" way.

    We revised the paragraph accordingly to highlight the novelty of our approach.

    Methods (former line 338):

    Besides simulating contour dynamics, our model is used to infer the different model components $f_{\text{prot}}, f_{\text{APCSF}}, f_{\text{AAF}}$ of experimental cell tracks to analyze and characterize cell tracks on the individual level. Often, the analysis of experimental contour dynamics is based on the cell's local motion, i.e., the velocity of the contour. However, the local motion comprises characteristics of the entire contour dynamics: protrusions, retractions, as well as minor membrane fluctuations. In our approach, by inferring the different model components, the protrusions and retractions can be quantified separately from each other. Furthermore, by estimating the corresponding component weights, different motility types, i.e., the amoeboid type and the fan-shaped type, can be classified.

13. Figure 7: Is the fluorescence imaging in panel B and D the same, i.e. both are imaging F-actin density? Later in Line 593, the text jumps directly to the saturation issue, but this is the first place for me to get the answer to this confusion. The definition of each plot needs to come before discussion of its saturation issue.

    We revised the Figure caption and the corresponding paragraph such that the connection of panels (B) and (D), i.e., the fluorescence microscopy image and the resulting fluorescence intensity kymograph, is now better clarified. Regarding a suggestion by the other Reviewer, we also added information about the biomarker signal (mRFP tagged LifeAct) to the paragraph and Figure caption.

    Results:

    In Fig 7A, contour dynamics of *D. discoideum* are displayed for a time period of $T = 500s$ and a frame rate of $\delta t = 1s$. This cell track is based on fluorescence microscopy data (mRFP tagged LifeAct, white to green color scheme), from which the cell contours are segmented (colored lines), see panel (B). As mentioned, we have chosen the 1st percentile of the entire area time series as underlying reference area, i.e., $A_{\text{ref}} = 91.23\mu m^2$. By following the approach from S1 Text, we estimated the following model weights: $w_{\text{prot}} = 6.634\,\mu m^2/s$, $w_{\text{APCSF}} = 0.057\,\mu m^2/s$, and $w_{\text{AAF}} = 3.532\,\mu m/s$. In comparison to parameter values estimated for other cell tracks, we observed that w_{prot} and w_{AAF} are relatively large while w_{APCSF} took a medium value. This observation coincides with the following cell track characteristics: a fast and persistent movement, an area time series within a smaller range

$90\mu m^2 < A < 120\mu m^2$ (see panel (F)), and contour dynamics showing an intermediately regularized curvature. The accuracy of our computational approach is demonstrated in panel (B), with virtual markers (black circles) being effectively propagated onto consecutive contours. In the following, we compare the inferred protrusion component (panel (E)) to common biomarkers: the local motion of the membrane (panel (C)) and the F-actin density/fluorescence intensity close to the membrane (panel (D)) based on the fluorescence microscopy data as shown in panel (B).

Caption Figure 7:

Inferring protrusion component from *D. discoideum* cell track. (A) Persistently motile cell track for $T = 500s$. **(B)** Microscopy image with fluorescence intensity (mRFP tagged LifeAct as marker for filamentous actin, white to green color scheme) and segmented cell contours (red lines, every tenth shown). **(C)** Local motion kymograph showing expansions (red areas) and retractions (blue areas). **(D)** Relative fluorescence intensity as in panel (B) with regions of high and low F-actin density displayed in red and blue, respectively. **(E)** The underlying protrusion component inferred from our model for a given set of model parameters. The resulting propagation of virtual markers from one contour to the next one is depicted as black circles in panel (B). **(F)** The contour area with predefined reference area $A_{\text{ref}} = 91.23\mu m^2$ (dashed gray line). Finally, regions of interest are displayed as black and white dashed boxes.

14. Line 604: what is the "main" characteristics? I understand the authors are trying to say Figure 7 panel D is more cluttered than C, but it could be due to color scale, nonlinear relationship between C and D, etc., and not necessarily because C has more details.

    We revised this paragraph and removed the expression "main characteristics". Instead, we argue that the saturation issue mentioned before is likely the reason that the fluorescence intensity kymograph is fuzzier/more cluttered than the local motion kymograph. In this context, we changed the range of the fluorescence intensity kymograph from 0.6 to 0.7 to avoid "hidden" peaks/extrema beyond the range of the kymograph plot.

    Results:

    In Fig 7, kymographs of all three quantities are displayed: the local motion (panel (C)), the fluorescence intensity (panel (D)), and the inferred protrusion component (panel (E)). We expect that a kymograph of a successfully inferred protrusion component would be strictly positive and only indicate regions where protrusions occurred. The remaining retractions are then covered by the other two components of our model: the AAF and the APCSF. In the local motion kymograph, local protrusions (red regions) and retractions (blue regions) are nicely shown. Due to a saturating fluorescence signal at high F-actin levels, we expect fewer details in the resulting fluorescence intensity kymograph for higher intensity levels. Indeed, the corresponding kymograph in panel (D) seems to be smoother than the local motion kymograph in panel (C), especially, for higher relative fluorescence intensity levels at around 0.6. The protrusion component kymograph in panel (E) mainly displays protrusive areas (red regions) with only very few negative regions (blue). We thus conclude that the missing retractive regions are successfully captured by the AAF and the APCSF.

Figure 7:

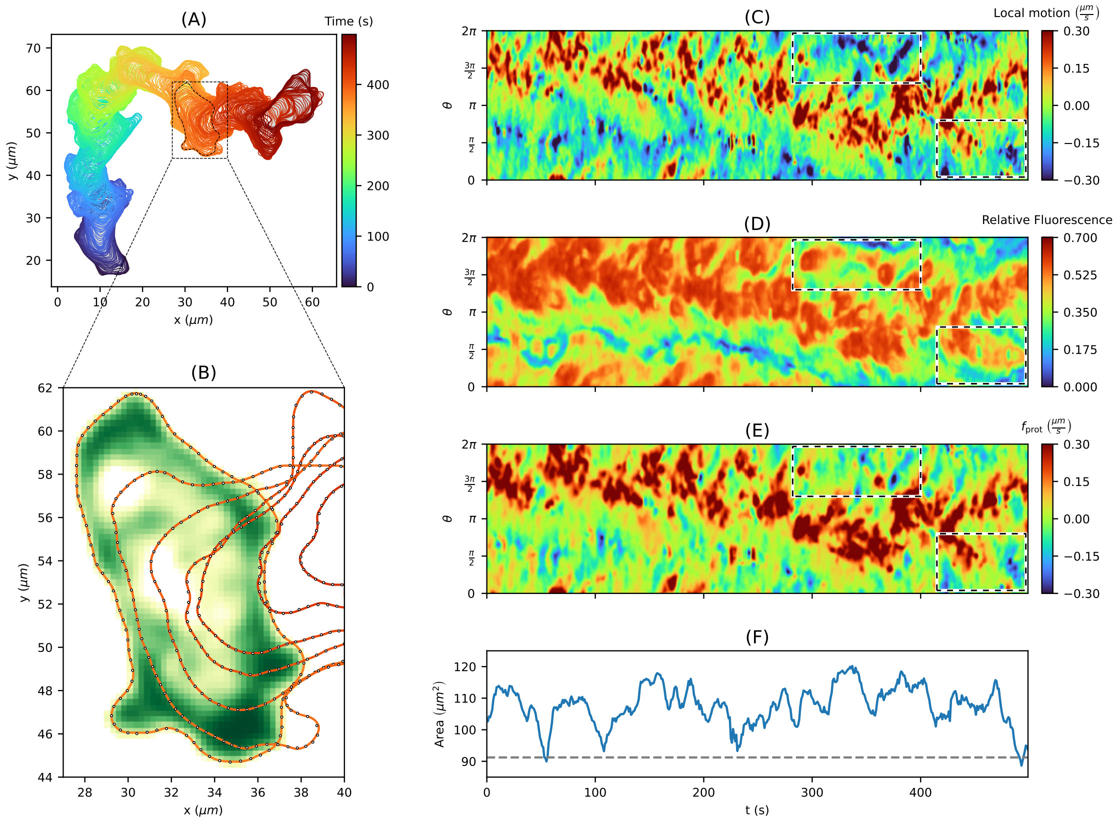

Fig 7. Inferring protrusion component from *D. discoideum* cell track.
Caption as above in point 13 (**Link to Caption**).

15. Line 605: please delete "a" before "very few".

    The phrase was changed as suggested.

16. Line 606: "We thus conclude that the missing retractive regions are successfully captured by the AAF and the APCSF." This is not rigorous to conclude AAF and the APCSF are responsible for the negative change based on that Protrusion is mostly positive. It's better to show summing up AAF and APCSF and protrusion equals the local membrane motion. That is done in S14Fig and Line 614, but at the point of line 606, the logic of expression is not optimal.

    We removed this statement (former Line 606) and revised the paragraph where the former S14 Fig (now S12 Fig) is introduced (former Line 614). Based on the different portions of the model components to the overall dynamics, the statement that the negative regions of the local motion are captured by the AAF and the APCSF is now mathematically founded and more rigorous.

    Results:

    In S12 Fig, the protrusion component from Fig 7 is displayed as well as the remaining components f_{APCSF} and f_{AAF}. As the underlying set of model weights, we used the same estimates mentioned above. The contributions of all three model components to the overall dynamics are shown for each time step/contour. In this context, we observed that the APCSF accounts for approximately 10% of

the overall dynamics. In contrast, the AAF and the protrusion component are more prominent accounting for approximately 40% and 50%, respectively. This may indicate that the APCSF mainly resolves a smaller number of strongly curved contour segments, whereas slower contour retractions at the rear of the cell are controlled by the AAF. As mentioned earlier, the protrusion component kymograph mainly consists of positive areas (red regions). Since the local motion comprises the summarized information of all three model components and due to the different portions of the AAF and APCSF to the overall dynamics (40% vs. 10%), we conclude that the retractions (blue regions) in the local motion kymograph in Fig 7 are primarily captured by the AAF and to a lesser extent by the APCSF.

17. Line 634: this is the same problem with λ_{reg}: definition of w_f and relative weights reside in SI but its quantitative analysis remains in main text. This approach doesn't make sense to me. It is as if saying to readers: just trust me when I tell you the effect of varying this parameter, and you cannot know why until you go to SI. Overall, it does't make sense to me to have a detailed paragraph in the main text that relies heavily on SI. Definition of quantities, quantitative analysis, most supporting plots should be together. For example, they could all be in SI, and the authors just mention briefly in main text that they analyzed influence of a priori model weights in SI, and mention the conclusion that the influence is minor.

    As suggested, we moved this section to the supporting information (S1 Text). A shortened version containing the main results of the model weights' influence remains in the main article.

    Results:

    Impact of *a priori* parameter choices on the analysis of experimental data. The F-actin density near the membrane is often interpreted as a marker of protrusive activity. Thus, we would expect some correlation between the F-actin density and the protrusion component in our model. Since the latter is influenced by the choice of the model weights of the two other model components AAF and APCSF, the question arises to what extent the correlation depends on the *a priori* choices of w_{AAF} and w_{APCSF}.

    In S1 Text, we rewrite the model equations from Eq (16) with respect to relative model weights instead of the absolute weights w_{prot}, w_{APCSF}, and w_{AAF}. An impact analysis of the varying model weights on the inferred protrusion component is also included in S1 Text. The inferred protrusion component kymographs resulting from this impact analysis are shown in S13 Fig. For the different configurations of model weights, we computed the Pearson correlation coefficient between the protrusion component and the Fluorescence intensity of the F-actin density, see S13 Fig.

    Briefly, we observed that the protrusion component inferred by the model is correlated to the underlying F-actin density. While the protrusion component is affected significantly by the parameter choice, the impact on the Pearson correlation coefficient is minor. In the case of the estimated model weights used in Fig 7, a Pearson correlation coefficient of $\rho = 0.47$ was computed, which is close to the Pearson correlation for the best fit ($\rho = 0.49$), see S1 Text and S13 Fig for details

18. Line 676: a major advantage of this model is the ability to accommodate a variety of contour dynamics, and even classify them. It's therefore much better to highlight more than one example of experimental cell tracks in main text, rather than keep referring to Supplemental Figures in main text.

As suggested, the supporting figures (former S11 Fig and S12 Fig) are now shown in the main text. We hope that the advantage of the model is now better presented.

Results:

In Fig 8, three tracks with an amoeboid motion as in Fig 7 are presented.

Figure 8:

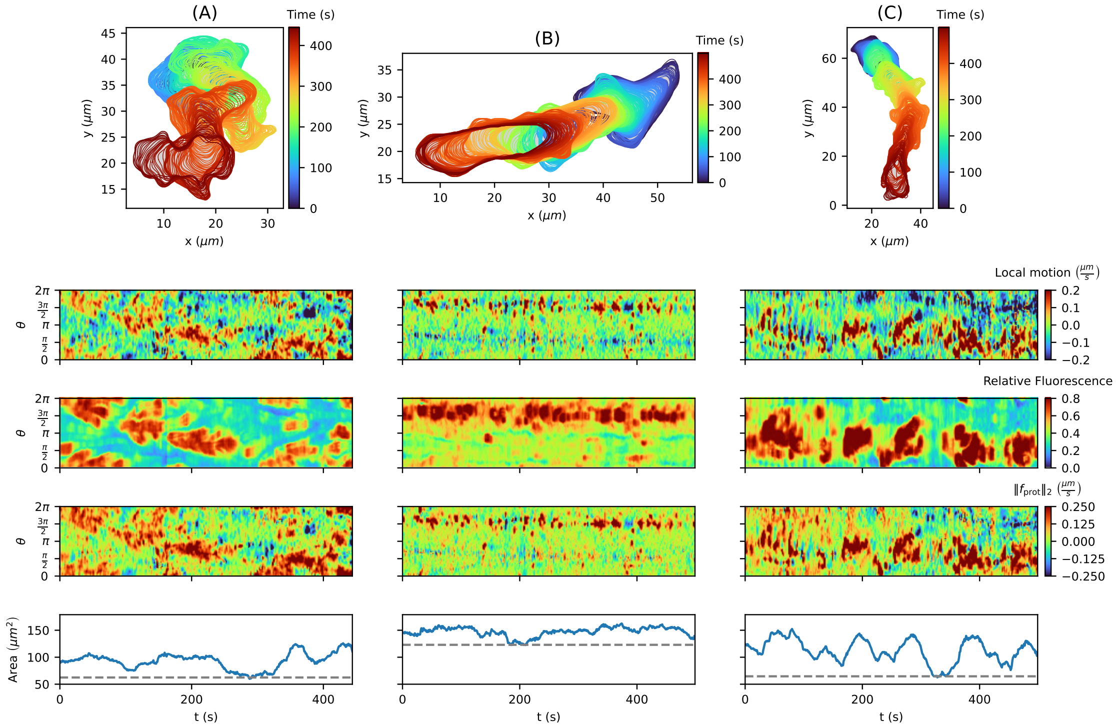

Fig 8. Collection of three *D. discoideum* tracks driven by the standard amoeboid type of cell motion with corresponding kymographs: Local motion, relative fluorescence intensity, the underlying protrusion component inferred from our model, and the contour area. For each cell track, the 1st percentile of the individual area time series (last row) was chosen as the reference area (gray dashed line) in our model: 62.43, 122.99, and 64.61 μm^2, respectively. The model weights (w_{prot}, w_{APCSF}, w_{AAF}) were estimated individually for each cell track: (4.513, 0.023, 0.902), (4.513, 0.048, 1.429), and (6.505, 0.070, 0.676), respectively from **(A)** to **(C)**.

Results:

In the case of the fan-shaped cells in Fig 9, we see clear blue stripes in the local motion kymograph at the rear of the cell, which corresponds to a smaller F-actin density in the fluorescence intensity kymograph.

Figure 9:

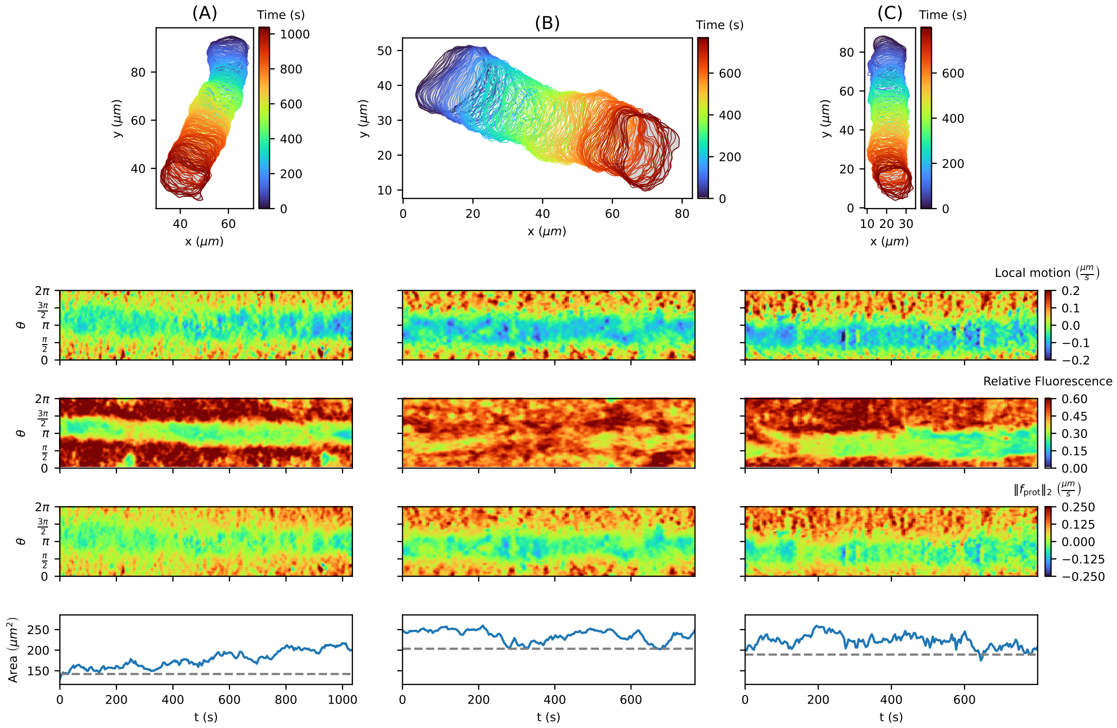

Fig 9. Collection of three *D. discoideum* tracks driven by a fan-shaped type of cell motion with corresponding kymographs: Local motion, relative fluorescence intensity, the underlying protrusion component inferred from our model, and the contour area. For each cell track, the 1st percentile of the individual area time series (last row) was chosen as the reference area (gray dashed line) in our model: 142.15, 203.45, and 189.24 μm^2, respectively. The model weights (w_{prot}, w_{APCSF}, w_{AAF}) were estimated individually for each cell track: (3.121, 0.000, 0.911), (4.485, 0.000, 1.844), and (4.428, 0.000, 1.760), respectively from **(A)** to **(C)**.

19. Line 833: I disagree based on the text that the approach is "fully" automated. It is indeed automated, but we have seen the necessity to make a priori selection of model weights. In year 2023, a typical example of "fully" automated computer program is chatGPT. For example, users don't need to make a priori hyper-parameter choices when using chatGPT.

    As suggested, we removed "fully" in this phrase.

    Discussion section:

    Due to a fast and straightforward parameter estimation and an automatized approach to infer protrusion and retraction characteristics, . . .

References

[1] Niculescu I, Textor J, de Boer RJ. Crawling and Gliding: A Computational Model for Shape-Driven Cell Migration. PLoS Computational Biology. 2015;11(10):e1004280. doi:10.1371/journal.pcbi.1004280.

[2] Wortel IMN, Niculescu I, Kolijn PM, Gov NS, de Boer RJ, Textor J. Local actin dynamics couple speed and persistence in a cellular Potts model of cell migration. Biophysical Journal. 2021;120(13):2609–2622. doi:10.1016/j.bpj.2021.04.036.

[3] Selmeczi D, Mosler S, Hagedorn PH, Larsen NB, Flyvbjerg H. Cell Motility as Persistent Random Motion: Theories from Experiments. Biophysical Journal. 2005;89(2):912–931. doi:10.1529/biophysj.105.061150.

[4] Maiuri P, Rupprecht JF, Wieser S, Ruprecht V, Bénichou O, Carpi N, et al. Actin flows mediate a universal coupling between cell speed and cell persistence. Cell. 2015;161(2):374–386. doi:10.1016/j.cell.2015.01.056.

[5] Schindler D, Moldenhawer T, Stange M, Lepro V, Beta C, Holschneider M, et al. Analysis of protrusion dynamics in amoeboid cell motility by means of regularized contour flows. PLoS Computational Biology. 2021;17(8):e1009268. doi:10.1371/journal.pcbi.1009268.

[6] Alonso S, Stange M, Beta C. Modeling random crawling, membrane deformation and intracellular polarity of motile amoeboid cells. PLOS ONE. 2018;13(8):e0201977. doi:10.1371/journal.pone.0201977.

[7] Moreno E, Flemming S, Font F, Holschneider M, Beta C, Alonso S. Modeling cell crawling strategies with a bistable model: From amoeboid to fan-shaped cell motion. Physica D: Nonlinear Phenomena. 2020;412:132591. doi:10.1016/j.physd.2020.132591.

[8] Löber J, Ziebert F, Aranson IS. Collisions of deformable cells lead to collective migration. Scientific Reports. 2015;5(1):9172. doi:10.1038/srep09172.

[9] Dreher A, Aranson IS, Kruse K. Spiral actin-polymerization waves can generate amoeboidal cell crawling. New Journal of Physics. 2014;16(5):055007. doi:10.1088/1367-2630/16/5/055007.

[10] Flemming S, Font F, Alonso S, Beta C. How cortical waves drive fission of motile cells. Proceedings of the National Academy of Sciences. 2020;117(12):6330–6338. doi:10.1073/pnas.1912428117.

[11] Moreno E, Großmann R, Beta C, Alonso S. From Single to Collective Motion of Social Amoebae: A Computational Study of Interacting Cells. Frontiers in Physics. 2022;9(February):1–17. doi:10.3389/fphy.2021.750187.
